# Inductive effect reversal drives efficient photocharging of catalytic sites in polymeric catalysts for continuous-flow H_2_O_2_ synthesis

**DOI:** 10.1093/nsr/nwag284

**Published:** 2026-05-14

**Authors:** Yang Zhang, Ning Cao, Fengting He, Yuzhao Wu, Xili Shang, Chaocheng Zhao, Shaobin Wang, Jinqiang Zhang

**Affiliations:** College of Chemical Engineering and Materials, Shandong University of Aeronautics, Binzhou 256600, China; College of Chemical Engineering and Materials, Shandong University of Aeronautics, Binzhou 256600, China; State Key Laboratory of Petroleum Pollution Control, China University of Petroleum (East China), Qingdao 266580, China; Institute for Smart Materials & Engineering, Jinan University, Jinan 250022, China; School of Chemical Engineering, The University of Adelaide, Adelaide, SA 5005, Australia; College of Chemical Engineering and Materials, Shandong University of Aeronautics, Binzhou 256600, China; State Key Laboratory of Petroleum Pollution Control, China University of Petroleum (East China), Qingdao 266580, China; School of Chemical Engineering, The University of Adelaide, Adelaide, SA 5005, Australia; School of Chemical Engineering, The University of Adelaide, Adelaide, SA 5005, Australia; School of Molecular Sciences, The University of Western Australia, Perth, WA 6009, Australia

**Keywords:** photocatalysts, crystalline carbon nitride, inductive effect, ether linkage, H_2_O_2_ photosynthesis

## Abstract

Polymeric photocatalysts show great promise for solar-driven H_2_O_2_ production, yet their structural-electronic correlations and scalability remain insufficiently elucidated. Herein, we reveal the pivotal role of the inductive effect in polymeric semiconductors. Cyano groups polarize the *π*-conjugated network in crystalline carbon nitride (CCN), generating a negative inductive effect that enhances charge dynamics and O_2_ adsorption but limits electron availability for oxygen reduction. Introducing ether linkages into ordered CCN chains (OCCN) reverses this effect, further facilitating charge separation and lowering the barrier for *OOH intermediate formation. Notably, the resulting positive inductive effect enables efficient photocharging of cyano groups with hot electrons. Consequently, OCCN delivers a remarkable H_2_O_2_ yield of 13 136.4 µmol g^−1^ h^−1^ in water, even higher performance in seawater, and sustains 110.4 µmol h^−1^ generation in continuous flow. These findings establish inductive effect engineering as a powerful approach for advancing polymeric photocatalysts toward scalable solar-to-chemical conversion.

## Teaser

This study elucidates and engineers the inductive effect in polymeric photocatalysts, enabling efficient coupling between reactive sites and electronic hotspots, thereby markedly enhancing sustainable H_2_O_2_ production.

## INTRODUCTION

**Figure 1.**
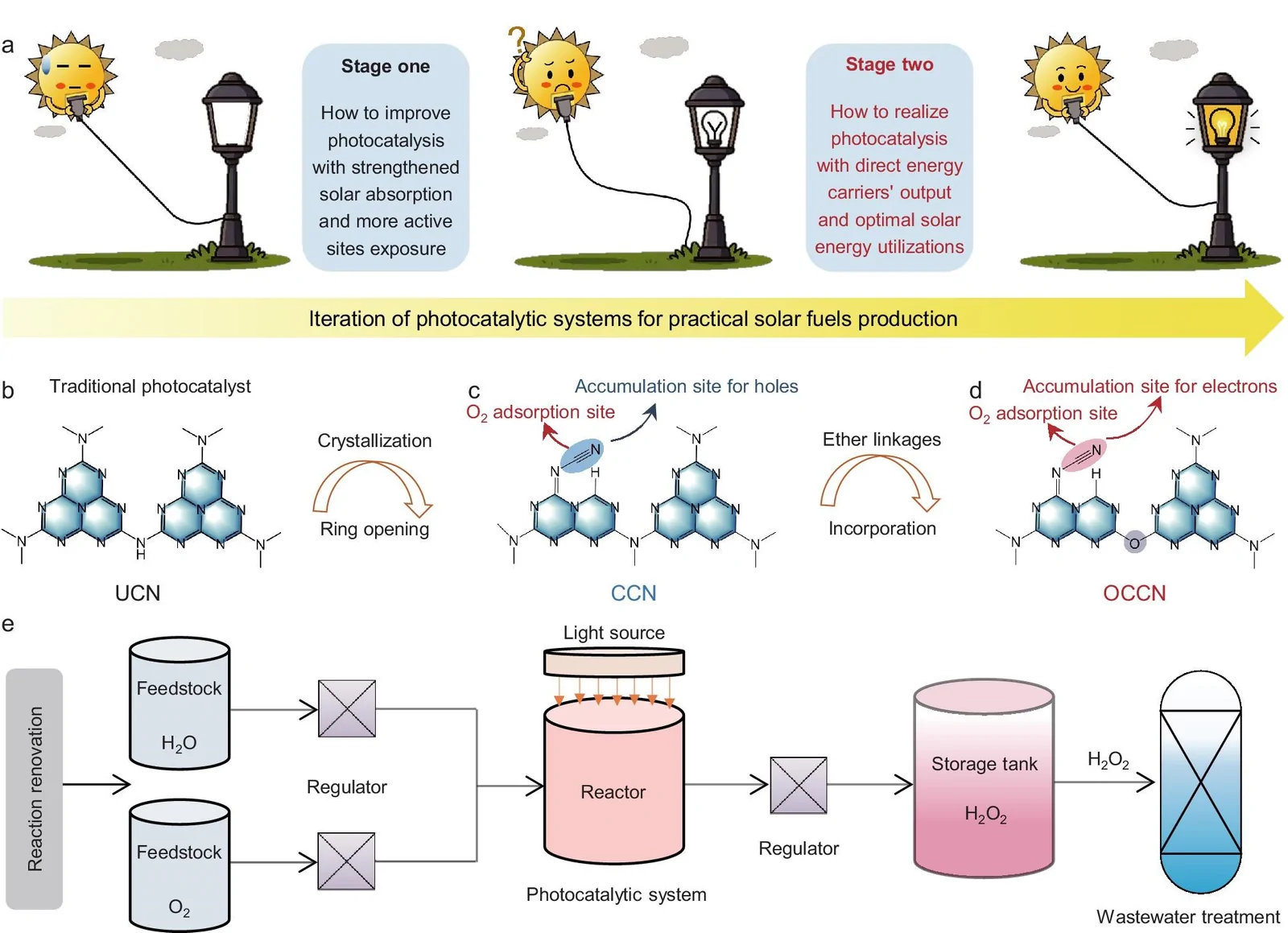
(a) Iterative processes of photocatalytic process toward scalable solar fuels production. Photocatalyst iteration including (b) UCN, (c) CCN, and (d) OCCN in this work. (e) System iteration by establishing continuous photocatalytic setup in this work for large scale H_2_O_2_ production.

Hydrogen peroxide (H_2_O_2_), a versatile and environmentally benign oxidant, is widely applied in medical disinfection, chemical synthesis, and environmental remediation [1,2]. However, its industrial production is predominantly reliant on the anthraquinone process, an energy-intensive, multistep route involving costly palladium-based catalysts and hazardous organic solvents, raising concerns regarding sustainability and safety [3]. By contrast, artificial photosynthesis of H_2_O_2_ via photocatalytic oxygen reduction has emerged as an appealing alternative, offering advantages such as lower energy input, improved environmental compatibility, and enhanced operational safety [4–6]. Over the past decade, tremendous efforts have been dedicated to advancing the efficiency of photocatalytic H_2_O_2_ synthesis. For instance, Yang *et al.* achieved a remarkable apparent quantum efficiency (AQE) of 30% at 420 nm by dynamically regulating Cu single-atom sites [7]. Feng *et al.* further constructed atomic-level Ru-Cu bifunctional units that enable ultrafast directional transfer of photogenerated carriers and significantly suppress electron-hole recombination, leading to a substantial enhancement in H_2_O_2_ production rates [8]. Additionally, precise regulation of local proton coverage via a multisite coupling strategy has been demonstrated as an effective approach to optimize the formation pathway of the key *OOH intermediate and mitigate the reaction energy barrier [9]. Nevertheless, the field remains limited by the scarcity of highly efficient photocatalysts capable of simultaneously maximizing photon utilization and selectively reducing O_2_ to H_2_O_2_ on a scalable level (Fig. 1a) [10].

Among various candidates, polymeric carbon nitride (CN), a metal-free photocatalyst, has garnered increasing attention owing to its visible-light responsiveness, suitable band alignment, and environmental benignity [11,12]. However, conventional amorphous CN (UCN), composed of high-symmetry heptazine units (Fig. 1b), suffers from inefficient charge transport, severe electron–hole recombination, and a low density of active sites, all of which result in limited H_2_O_2_ yield [13]. In contrast, crystalline carbon nitride (CCN) features an ordered molecular structure and extended *π*-delocalized electronic states, which enhance light absorption and charge separation, thereby markedly improving photocatalytic performance. In our previous work, a CCN-based photocatalyst achieved a record-high solar-to-chemical conversion efficiency of 2.3% for H_2_O_2_ photosynthesis, significantly outperforming state-of-the-art systems and exceeding the natural photosynthetic efficiency of plants (<0.1%) [14]. Nonetheless, further performance enhancement in CCN-based H_2_O_2_ photosynthesis and scalability requires a deeper mechanistic understanding of its structure–activity relationship.

Inductive effect is a fundamental electronic phenomenon in polymeric semiconductors, arising from the presence of electron-withdrawing (−*I*) or electron-donating (+*I*) groups along the polymer backbone. This effect critically influences their electronic structures, charge transport properties, and surface reaction pathways in photocatalysis. In pristine CN, the inductive effect is relatively weak due to the high symmetry and periodicity of the heptazine units. However, CCN synthesized via molten salt methods exhibits structural distortions that expose terminal cyano groups, which impose a strong −*I* effect. This substantially redistributes electron density, enhances charge separation, and modifies the band structure [15,16]. Although these changes benefit photocatalytic activity, the precise role of the inductive effect in CCN remains poorly understood. Therefore, unveiling and rational engineering of this effect is crucial to fully unlocking the photocatalytic potential of CCN, yet it remains largely unexplored.

In this study, we systematically investigated the role of cyano groups in CCN for H_2_O_2_ photosynthesis. We found that while these groups significantly promoted charge carrier dynamics and surface reaction kinetics, they also led to undesirable electron accumulation. This misalignment between electron-rich regions and oxygen reduction sites ultimately hindered the formation of H_2_O_2_ (Fig. 1c). To address this, we introduced electronegative oxygen atoms into the CCN lattice to incorporate ether linkages into ordered CCN chains (OCCN). This structural modification reinforced the built-in electric field (BIEF), facilitating more efficient charge separation and transport. More importantly, it reversed the local inductive effect, thereby aligning the O_2_ reduction sites with photogenerated electron accumulation sites at the cyano groups (Fig. 1d). The resulting charge redistribution also lowered the thermodynamic barrier for *OOH formation at cyano sites, a key intermediate in the two-electron reduction pathway for H_2_O_2_ generation. Consequently, OCCN achieved an outstanding H_2_O_2_ production rate of 13136.4 μmol g^−1^ h^−1^, which was 66.0 times higher than that of UCN and 2.8 times higher than CCN. OCCN also demonstrated a high AQE of 28.2% at 420 nm and even greater H_2_O_2_-generation in natural seawater, highlighting its potential for real-world applications. Notably, OCCN maintained exceptional stability and durability, achieving a sustained H_2_O_2_ production rate of 110.4 μmol h^−1^ in a continuous 24-h flow system (Fig. 1e), showcasing its strong potential for scalable solar fuel production. These results highlight the pivotal role of inductive effect modulation in polymeric photocatalysts, offering new design principles for achieving scalable and sustainable solar fuel production.

## RESULTS AND DISCUSSION

### Identification of cyano group and its roles in CCN

**Figure 2.**
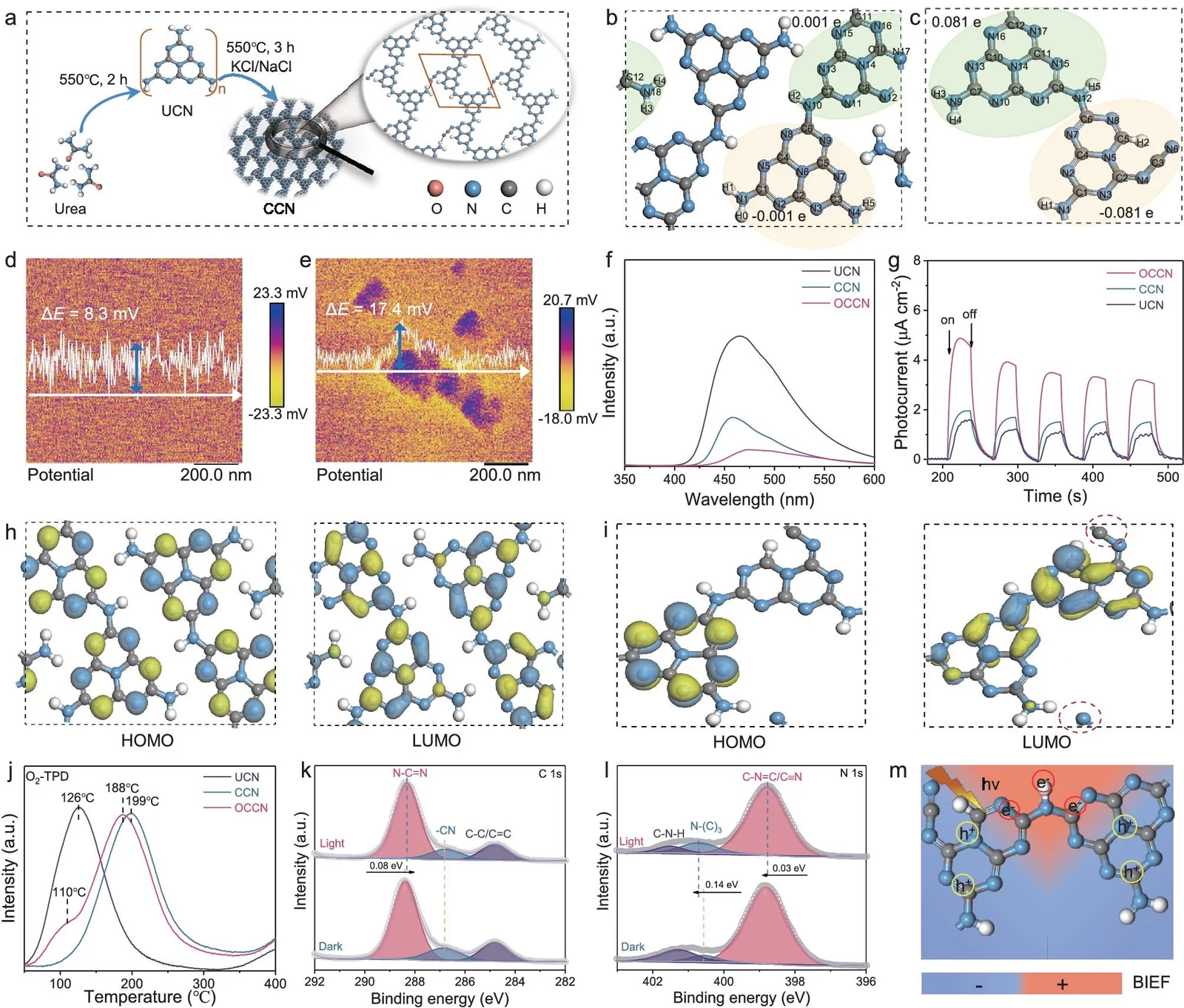
(a) Synthetic route of CCN. Mulliken charge distribution of (b) UCN and (c) CCN. KPFM images of (d) UCN and (e) CCN. (f) PL spectra and (g) transient photocurrent responses of prepared samples. HOMO and LUMO distribution of (h) UCN and (i) CCN. (j) O_2_-TPD profiles of prepared samples. *In-situ* light irradiation XPS spectra for (k) C 1s and (l) N 1s of CCN. (m) Mechanism of BIEF to promote the separation of photogenerated carriers on CCN.

In this work, UCN was obtained via the conventional thermal polycondensation of urea at 550°C for 2 h, while CCN was synthesized by further calcining UCN in a molten salt medium, both serving as model photocatalysts to probe the mechanistic role of inductive effect in H_2_O_2_ photosynthesis (Fig. 2a) [17]. Microstructural analysis using scanning electron microscopy and transmission electron microscopy revealed that UCN exhibited a bulk morphology composed of amorphous nanosheets (Figs S1a and S2a), whereas CCN displayed a well-defined crystalline structure with periodic stripe spacing of 1.01 nm (Figs S1b and S2b), consistent with the (100) crystal plane observed in powder X-ray diffraction (PXRD) patterns (Fig. S3). Fourier-transform infrared (FT-IR) spectroscopy (Fig. S4) confirmed that the fundamental chemical features of UCN, including surface −NH*_x_* groups (3600–3100 cm^−1^), C–N heterocycles (1700–1200 cm^−1^), and heptazine rings (C_3_N_3_) (810 cm^−1^), were largely preserved after molten salt calcination [18,19]. Notably, a distinct peak at 2180 cm^−1^ emerged in the CCN spectrum, corresponding to the asymmetric stretching vibration of cyano groups (−C≡N), indicating partial ring-opening of heptazine units during the crystallization process [20].

To elucidate the chemical structure of the CCN unit cell, solid-state ^13^C nuclear magnetic resonance (NMR) spectroscopy measurement was conducted. Compared to UCN, which exhibits two prominent peaks at 164.9 and 156.9 ppm corresponding to the C(1) atom (terminal N_2_C–NH*_x_*) and C(2) atom (N=C–N_2_), respectively (Fig. S5a), the spectrum of CCN displays new peaks at 121.9, 168.4 and 170.9 ppm [21]. These signals are assigned to the C(3) atom (N–C≡N), C(4) and C(5) atoms, respectively, confirming the ring-opening of heptazine units and thereby defining the molecular structure of the CCN unit cell (Fig. S5b) [13,22].

X-ray photoelectron spectroscopy (XPS) further investigated the elemental composition and chemical environment of CCN. In C 1s spectra of UCN, three characteristic peaks appeared at 284.8 eV (adventitious carbon), 286.1 eV (amino groups), and 288.0 eV (sp²-hybridized carbon) (Fig. S6a) [23]. For CCN, an additional peak emerged at ∼286.9 eV, attributed to newly formed cyano groups (−C≡N), in agreement with FT-IR and NMR findings [24]. N 1s spectra of UCN can be deconvoluted into three components at ∼398.5 eV (sp^2^ hybridized nitrogen, C–N=C), ∼400.1 eV (tertiary nitrogen, N–(C)_3_), and ∼401.2 eV (terminal amino, C–N–H) (Fig. S6b) [25]. Notably, all corresponding peaks in CCN shifted toward higher binding energies, indicating in-plane charge redistribution and reduced electron density in the preserved heptazine units, resulting from the inductive effect of the newly introduced cyano groups [26]. In addition, K and Na signals were detected in the XPS survey spectra of CCN and OCCN, suggesting the incorporation of K^+^ and Na^+^ ions into the CN framework (Fig. S7). More importantly, the atomic proportion of oxygen in OCCN was remarkably higher than that in CCN, while the content of residual metal ions in the OCCN framework was lower than that in CCN (Table S1).

To further quantify the inductive effect, charge distribution calculations were performed based on the proposed chemical structures (Fig. S5). In UCN, the highly symmetric unit cell resulted in Mulliken charge values of −0.001 e and 0.001 e for adjacent heptazine units, suggesting a negligible inductive effect. In contrast, in CCN, the cyano group, owing to its strong electronegativity, draws the bonding electron cloud toward itself, resulting in a charge value of −0.081 e for the disrupted heptazine unit and 0.081 e for the opposing unit. This clearly demonstrates a notable negative inductive effect (Fig. 2b, c and Table S2).

The influence of this inductive effect on the photoelectrical and catalytic properties of the polymeric semiconductors was systematically investigated. Electrostatic potential distributions revealed a significantly enhanced potential gradient across the CCN intralayer, attributed to the negative inductive effect. This enhancement strengthens the planar BIEF, which in turn provides a greater driving force for the separation and transport of photoexcited charge carriers (Fig. S8). The increased surface polarization potential (Δ*E*) at the nanoscale was experimentally confirmed by Kelvin probe force microscopy (KPFM), which showed that CCN exhibited a Δ*E* of 17.4 mV, more than twice that of UCN (8.3 mV) (Fig. 2d and e). In contrast, the morphology of UCN is barely distinguishable in the KPFM images, which is mainly attributed to its intrinsically weak surface potential [27]. A consistent trend was observed in the Zeta potential measurements, where the Zeta potentials of UCN and CCN were −18.2 and −23.3 mV, respectively, meaning a stronger electrostatic driving force along CCN for the migration of photogenerated electron–hole pairs (Fig. S9).

Indeed, successive characterizations verified the enhanced photoexcited charge dynamics in CCN. Compared to UCN, CCN showed a stronger electron paramagnetic resonance (EPR) signal (Fig. S10), a quenched fluorescence peak in the photoluminescence (PL) spectrum (Fig. 2f), a smaller arc radius in electrochemical impedance spectroscopy (EIS) (Fig. S11), and an intensified transient photocurrent response (Fig. 2g). These results suggest that the unique structure of CCN enables more efficient hot carrier generation and improved charge separation. Orbital analysis provided additional evidence: the originally symmetric highest occupied molecular orbitals (HOMO) and lowest unoccupied molecular orbitals (LUMO) in UCN became significantly spatially separated in CCN, which facilitates charge migration and suppresses electron–hole recombination (Fig. 2h and i). It is noteworthy that for CCN, the LUMO is predominantly localized on the central aromatic nuclei, whereas the contribution from the cyano groups is negligible. This suggests that cyano groups do not serve as effective electron-accepting sites for photogenerated electrons (Fig. 2i).

In addition, the crystalline structure and exposed cyano group of CCN contributed to its superior light-harvesting properties, with stronger UV and visible light absorption and a markedly red-shifted absorption edge compared to UCN (Fig. S12). Besides, O_2_ temperature-programmed desorption (O_2_-TPD) was used to assess oxygen adsorption, which is crucial for H_2_O_2_ photosynthesis (Fig. 2j). Results indicated that the negative inductive effect of CCN caused by exposure of cyano groups intensified the adsorption strength of oxygen, as revealed by the emergence of chemisorption of O_2_ on CCN at 199°C compared to the physisorption of O_2_ on UCN at a lower temperature of 126°C, thereby significantly improving mass transfer during the photocatalytic oxygen reduction process.

Then the charge accumulation behavior in CCN was revealed using *in-situ* irradiation XPS. C 1s spectra showed that the peak position of N–C=N shifted slightly to lower binding energy by 0.08 eV after irradiation (Fig. 2k). In contrast, in the N 1s spectra (Fig. 2l), the peaks of C–N=C/C≡N and N–(C)_3_ shifted to higher binding energy. These shifts suggest that photogenerated electrons accumulate on the sp^2^-hybridized C atoms, while holes mainly localize on the tertiary nitrogen atoms (Fig. 2m). This charge separation inhibits the oxygen reduction reaction at cyano groups. Therefore, the asymmetric chemical structure of CCN induces a strong negative inductive effect, which enhances the BIEF and significantly improves charge separation and O_2_ adsorption at disrupted heptazine rings. However, this same structural feature appears to hinder the accumulation of hot electrons (i.e. nonthermalized photoexcited electrons with excess energy above the conduction band edge) at these sites, leading to a spatial mismatch between active sites and photogenerated electrons, thereby limiting the efficiency of redox reactions.

### Reversing inductive effect in OCCN

**Figure 3.**
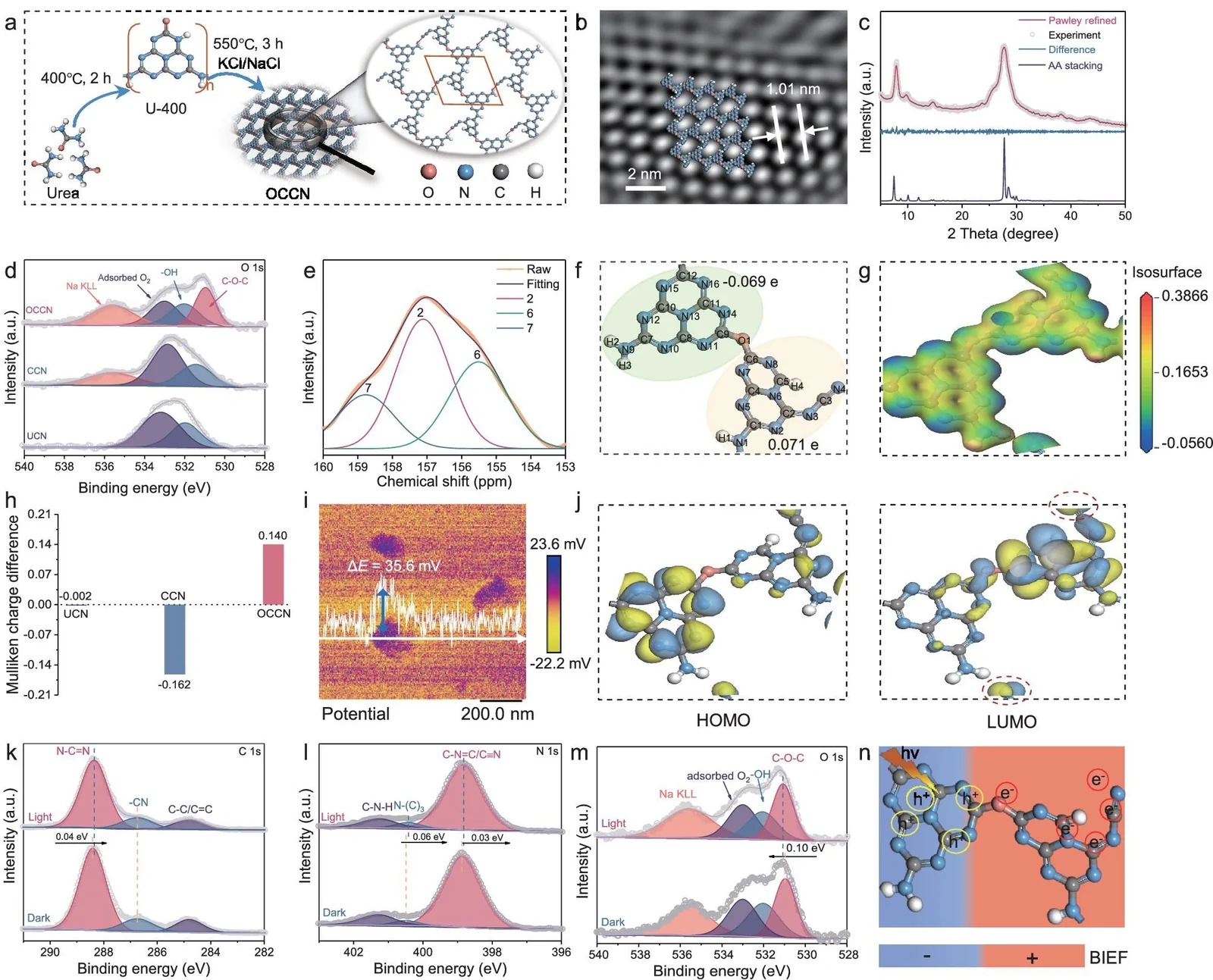
(a) Synthetic route of the OCCN. (b) Fourier-filtered image of the selected white square area in Fig. S19. (c) PXRD patterns of OCCN. (d) XPS spectra for O 1s of OCCN. (e) Magnified view of ^13^C NMR spectra for OCCN. (f) Mulliken charge distribution of OCCN. (g) Electrostatic potential distribution of OCCN. (h) Mulliken charge difference of UCN, CCN, and OCCN. (i) KPFM image of OCCN. (j) HOMO and LUMO distribution of OCCN. *In-situ* light irradiation XPS spectra of (k) C 1s, (l) N 1s, and (m) O 1s of OCCN. (n) Mechanism of BIEF to promote the separation of photogenerated carriers on OCCN.

Based on the above analyses, both the advantages and drawbacks of the negative inductive effect within CCN have been identified, ultimately leading to suboptimal solar conversion efficiency. To optimize this effect, ether linkages were introduced into the polymer chain of CCN, resulting in a modified polymer referred to as OCCN. Specifically, an oligomer (U400) was first synthesized via thermal polycondensation of urea in air at 400°C. The presence of carbonyl oxygen in U400 was confirmed by FT-IR and near-edge X-ray absorption fine structure spectroscopy (Figs S13–S15). This oligomer was subsequently polymerized in a molten salt medium, which facilitated deamination and condensation reactions to yield OCCN (Fig. 3a).

Microstructure observation of OCCN revealed well-dispersed nanoparticles with an average size of about 91.1 nm, exhibiting clear lattice fringes indicative of high crystallinity (Figs S16 and S17). Atomic force microscopy images further showed that OCCN nanoparticles had a thickness of about 32.9 nm, significantly greater than the 5.8 nm thickness of UCN nanosheets. This increase suggests that the incorporation of ether linkages enhanced the degree of polymerization and extended the *π*-conjugated system (Fig. S18). Moreover, the periodic framework of OCCN was clearly visible in the high-resolution transmission electron microscopy image (Fig. S19). Fourier-filtered image of a magnified region revealed an interplanar spacing of 1.01 nm for the (100) crystal plane (Fig. 3b), and elemental mapping analysis confirmed the homogeneous distribution of C, N, and O elements throughout the OCCN structure (Fig. S20).

The crystalline structure of OCCN was also characterized using PXRD, which exhibited two typical peaks at 27.8° and 7.8°, corresponding to interlayer stacking along the (002) plane and intra-planar unit stacking along the (100) plane, respectively. In addition, weaker peaks at 9.9° and 14.5° were assigned to the (110) and (200) planes, respectively (Fig. S3). The sharp and intense (100) peak further proved the high crystallinity of OCCN [17,28]. Pawley refinement (red curve) showed excellent agreement between the experimental and simulated PXRD patterns, with minimal differences (blue curve) (Fig. 3c).

FT-IR spectrum of OCCN displayed characteristic vibrational peaks of C–H (995 cm^−1^) and C–O–C (1155 cm^−1^), in addition to the peaks inherited from CCN (Fig. S4). These features indicate that the terminal oxygen atoms in U400 were successfully converted into C–O–C, bridging the heptazine unit [29,30]. The presence of ether bonds in OCCN was further confirmed by the O 1s XPS spectrum, which was deconvoluted into four peaks: 531.0 eV (C–O–C), 532.1 eV (−OH), 533.3 eV (adsorbed O_2_), and 535.9 eV (Na KLL) (Fig. 3d) [31]. The chemical structure of the OCCN unit cell was further elucidated by solid-state ^13^C NMR. A strong resonance at 163.7 ppm was attributed to the C(1) atom (terminal N_2_C–NH*_x_*), while signals at 125.0, 168.6, and 171.4 ppm corresponded to the C(3), C(4), and C(5) atoms, respectively (Fig. S21) [21]. Notably, the broad peak centered near 157 ppm (highlighted in the blue-shaded region) can be deconvoluted into distinct contributions at 157.1, 155.5, and 158.8 ppm, corresponding to the C(2), C(6), and C(7) atoms, respectively (Fig. 3e). Collectively, these FT-IR, XPS, and NMR results confirm that the bridged nitrogen atom present in the molecular structure of CCN has been replaced by an oxygen atom in the unit cell of OCCN.

Charge distribution calculations for OCCN revealed that the heptazine unit bearing the cyano group became a positively charged region with a net charge of 0.071 e, while the adjacent unit exhibited a negative charge of −0.069 e (Fig. 3f and Table S2 for detailed numerical values). Electrostatic potential mapping further demonstrated that the incorporation of ether linkages into the CCN lattice elevated the local potential at the cyano group (Fig. 3g). The calculated Mulliken charge differences between two adjacent heptazine units bridged by nitrogen or oxygen atoms in UCN, CCN, and OCCN were −0.002 e, −0.162 e, and 0.140 e, respectively, clearly confirming a reversal of the inductive effect upon ether bond introduction (Fig. 3h).

The impact of this reversed inductive effect on the photoelectric properties of the material was systematically investigated. First, the enhanced polymerization and incorporation of ether bonds broadened the light absorption range of OCCN (Fig. S12). KPFM measurement showed that the Δ*E* of OCCN increased to 35.6 mV, approximately twice that of CCN, indicating a significantly strengthened BIEF (Figs 3i and 2e). Similarly, OCCN exhibited a more negative Zeta potential (−27.5 mV) compared to CCN (Fig. S9), reflecting a stronger electrostatic driving force for efficient separation and transport of photogenerated charge carriers. A monotonic functional relationship exists between the BIEF and both the surface charge density and surface potential, as derived from KPFM and Zeta potential measurements (Supplementary Data). Based on the Kanata model [32], BIEF was quantitatively calculated based on the measured Zeta potential and KPFM surface potential. The results showed that the BIEF strength followed the order OCCN > CCN > UCN, with OCCN exhibiting values 2.6 and 1.6 times higher than those of UCN and CCN, respectively. These quantitative findings confirm that the introduction of C–O–C linkages effectively enhances the BIEF. The trend in BIEF was further supported by EPR, PL, EIS, and photocurrent measurements, all of which confirmed superior charge separation and mobility in OCCN compared to CCN (Fig. 2f and g, Figs S10 and S11).

Interestingly, the introduction of ether linkages modified the oxygen adsorption behavior. While OCCN exhibited weakened adsorption at the original strong binding sites, another weak adsorption site at 110°C emerged (Fig. 2j). This phenomenon can be attributed to the electron-withdrawing nature of the ether bond, which reduces the electron density on the cyano group, consistent with charge distribution data (Figs 2c and 3f, Table S2). Importantly, this modulation of electron density also altered the orbital distribution, relocating the LUMO to the cyano-bearing heptazine unit and increasing the contribution of cyano groups to the LUMO. This indicates that photogenerated electrons can be directly injected into the cyano sites (Fig. 3j).

The origin of this inductive effect reversal was further investigated through projected density of states (PDOS) analysis. The results reveal that both the ether bridge (C–O–C) in OCCN and the amine bridge (C–N–C) in CCN exhibit significant orbital overlap with adjacent cyano groups at comparable energy levels (Fig. S22). The well-aligned peak positions and resonance features indicate strong orbital hybridization among the bridging units, cyano groups, and the conjugated CN backbone, effectively extending the delocalization of *π* electrons.

In OCCN, the strong orbital hybridization among the ether linkage, cyano group, and triazine rings precisely modulated the local electron density distribution within the conjugated system. The electronegative oxygen atom in the ether linkage not only delocalized its lone pair electrons into the conjugated backbone via *p*-*π* conjugation but also modulated the electron affinity of the adjacent cyano group through σ inductive effect (electron-withdrawing). The synergistic interplay of these two electronic effects was identified as the intrinsic origin of the inductive effect reversal. Furthermore, PDOS analysis reveals that, compared with CCN, the cyano-derived LUMO states in OCCN shift to lower energy, rendering the cyano groups effective electron-accepting sites. This observation is consistent with the frontier orbital analysis and confirms that the reversal of the inductive effect arises from the combined modulation of orbital coupling and orbital distribution.

*In-situ* light irradiation XPS was again conducted to elucidate the role of ether bonds in charge transfer and accumulation. C 1s spectra showed a slight shift (−0.04 eV) in the N–C=N peak after illumination (Fig. 3k), while the N 1s peaks corresponding to C–N=C/C≡N and N–(C)_3_ shifted to lower binding energies by 0.03 eV and 0.06 eV, respectively (Fig. 3l). In contrast, O 1s peak corresponding to C–O–C bonds shifted to a higher binding energy by 0.10 eV under irradiation (Fig. 3m). These shifts suggest that photogenerated electrons accumulate at the nitrogen atoms of the cyano group, whereas photogenerated holes preferentially accumulate at the atoms near the ether bond. Collectively, these findings demonstrate that the reverse of inductive effect in OCCN further enhanced charge separation and importantly aligned the oxygen adsorption sites with the hot electron accumulation zones, thereby promoting the output of hot carriers into redox reactions (Fig. 3n).

### Performance evaluation of H_2_O_2_ photosynthesis

Numerous studies have demonstrated that CN and its derivatives generally exhibit indirect semiconductor characteristics [33,34]. Accordingly, Tauc plots derived from UV-Vis diffuse reflectance spectroscopy revealed the band gaps of UCN, CCN and OCCN to be 2.63, 2.60 and 2.57 eV, respectively (Fig. S23). Meanwhile, Mott-Schottky plots for all three materials exhibited positive slopes, confirming their n-type semiconductor characteristics [35]. The flat band potentials were estimated to be −1.50 V (UCN), −1.30 V (CCN), and −1.17 V (OCCN) (vs. Ag/AgCl) (Fig. S24). By combining these values with the corresponding bandgaps, the conduction band (CB) and valence band (VB) positions were calculated (Fig. S25). These results suggest that tailoring the inductive effect of CN framework maintains favorable thermodynamic driving forces for H_2_O_2_ production via the two-electron oxygen reduction reaction (ORR, *E*_NHE_ = 0.68 V) [36].

**Figure 4.**
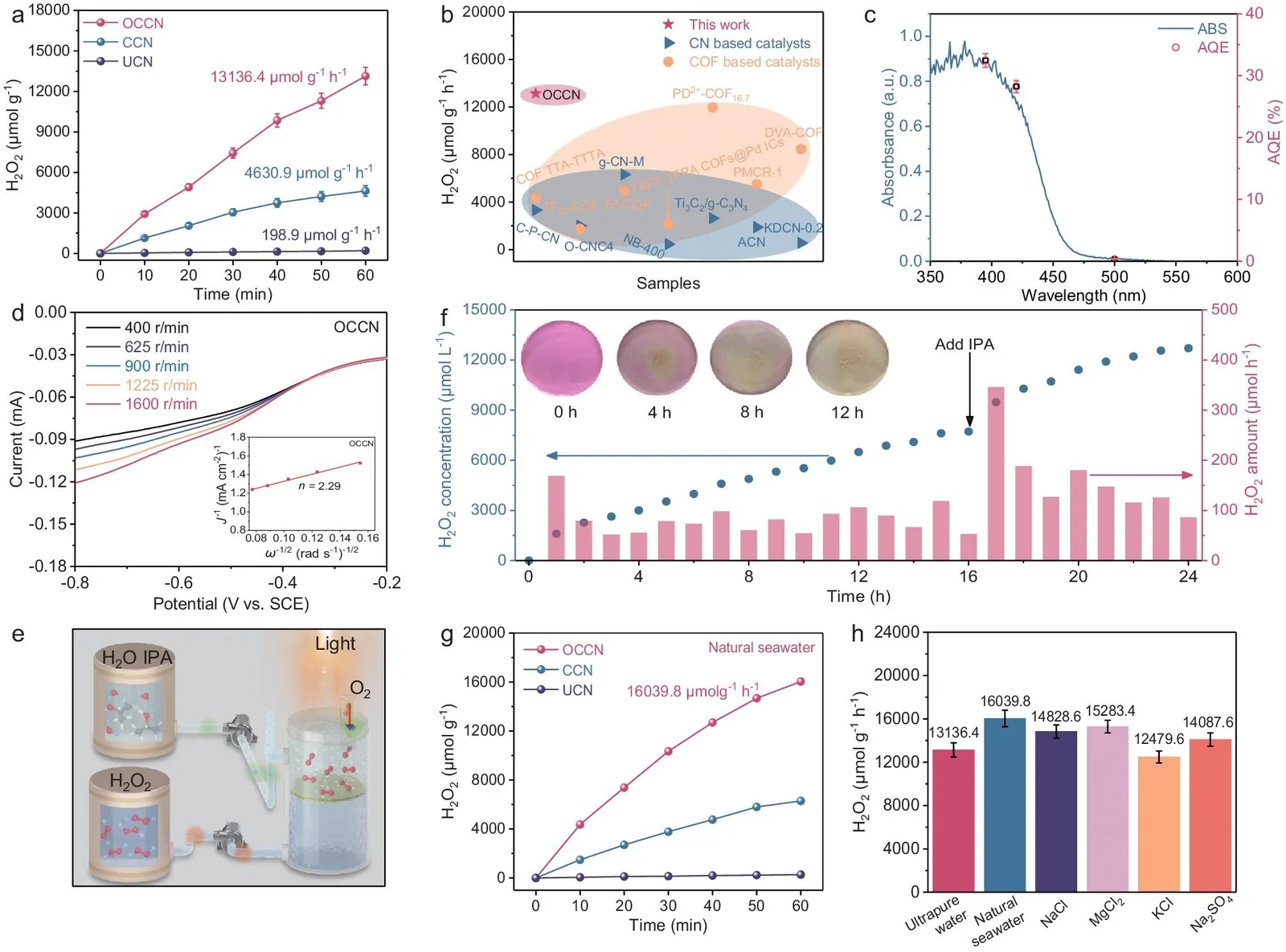
(a) Time-dependent H_2_O_2_ photogeneration. (b) Comparison of metal-free photocatalysts for H_2_O_2_ production. (c) Wavelength-dependent AQE of OCCN for H_2_O_2_ generation. (d) LSV curves of OCCN measured using a rotating disk electrode. The inset shows the fitted K-L plot. (e) Schematic diagram of the continuous photocatalytic system. (f) Concentration of H_2_O_2_ produced versus time and H_2_O_2_ production per hour in a continuous flow system. (g) Time-dependent H_2_O_2_ photogeneration in natural seawater. (h) H_2_O_2_ production performance of OCCN in ultrapure water, natural seawater, 2.7 wt% NaCl solution, 0.5 wt% MgCl_2_ solution, 0.07 wt% KCl solution, and 3.3 wt% Na_2_SO_4_ solution.

The photocatalytic performance of the samples was evaluated under visible light irradiation in an O_2_-saturated aqueous solution containing 10 vol% isopropanol (IPA). As displayed in Fig. 4a, H_2_O_2_ production increased steadily over time, with yields of 198.9 and 4630.9 μmol g^−1^ h^−1^ for UCN and CCN, respectively. The performance of CCN is more than 23 times higher than UCN, attributing to the enhanced charge separation and mass transfer efficiency induced by the negative inductive effect. Remarkably, OCCN, which features a reversed (positive) inductive effect due to the incorporation of ether bonds, achieved the highest H_2_O_2_ yield of 13 136.4 μmol g^−1^ h^−1^. This corresponds to enhancements of 66.0 and 2.8 times compared to UCN and CCN, respectively. Notably, this performance surpasses that of most previously reported metal-free photocatalysts (Fig. 4b and Table S4), underscoring the effectiveness of inductive effect engineering in optimizing solar energy utilization [37–50]. The AQE of OCCN were measured to be 32.5% and 28.2% under irradiation at 395 nm and 420 nm, respectively, in good agreement with its light absorption profile (Fig. 4c and Table S5).

The photocatalytic activity and selectivity of the samples toward ORR were further investigated using a rotating disk electrode (RDE) and a rotating ring disk electrode (RRDE) setup. The linear sweep voltammograms (LSVs) of the samples were recorded at different rotation speeds. Based on the current density at −0.67 V (vs. SCE), the Koutecky-Levich (K-L) curves were fitted, and the average electron-transfer number (*n*) values of UCN, CCN, and OCCN were determined to be 2.98, 2.56, and 2.29, respectively (Fig. 4d and Fig. S26) [50]. The lower *n* value for OCCN indicates a stronger preference for the two-electron transfer pathway (*n* ≈ 2), which is favorable for H_2_O_2_ production. Additionally, the potential was scanned from −0.6 to −0.1 V (vs Ag/AgCl) in O_2_-saturated 0.1 M KOH electrolyte at 1600 r/min. In this configuration, the disk current reflects the ORR occurring on the catalyst surface (via either a 2-electron or 4-electron pathway), while the ring current quantifies the H_2_O_2_ produced and subsequently oxidized [51]. As illustrated in Fig. S27, both disk and ring currents increased with the applied potential, with OCCN exhibiting significantly higher values than both UCN and CCN. Moreover, OCCN exhibited a lower onset potential, indicating more favorable kinetics for the selective 2-electron ORR pathway leading to H_2_O_2_ production. The average number of electron transfers (*n*) in the potential range of 0.56 to 0.68 V (vs RHE) was calculated to be 3.13 for UCN, 2.59 for CCN, and 2.34 for OCCN. Correspondingly, H_2_O_2_ selectivity was determined to be 43.4%, 70.5%, and 82.9%, respectively (Fig. S28). These results confirm that inductive effect engineering within polymeric frameworks significantly enhances both the selectivity and efficiency of the two-electron ORR process.

Stability experiments revealed that OCCN showed good stability by cycling tests over four consecutive runs (Fig. S29), without structural and crystalline changes (Fig. S30). More importantly, continuous H_2_O_2_ production was conducted in a custom-built flow system operating at a flow rate of 5 mL h^−1^ (Fig. 4e and Fig. S31). As shown in Fig. 4f, H_2_O_2_ concentration increased linearly over time and plateaued after 16 h due to the depletion of IPA. Upon replenishment with 10 mL IPA, H_2_O_2_ production resumed, achieving an average production rate of 110.4 μmol h^−1^ and a cumulative yield exceeding 2649.6 μmol over 24 h. To demonstrate the practical utility of the collected H_2_O_2_, the mixed solution containing H_2_O_2_ was employed in a Fenton reaction for wastewater treatment using Rhodamine B (20 mg L^−1^) as a model pollutant [52]. Complete degradation of Rhodamine B was achieved within just 60 seconds using the mixed solution containing H_2_O_2_ collected after 12 h of continuous operation (inset in Fig. 4f), highlighting its potential for coupling with environmental remediation plants.

Remarkably, OCCN achieved an even higher photocatalytic H_2_O_2_ yield of 16 039.8 μmol g^−1^ h^−1^ in natural seawater (Yellow Sea, Qingdao) compared to that in deionized water (Fig. 4g). To elucidate the origin of this enhanced performance, the H_2_O_2_ yield of OCCN was evaluated in single-component salt solutions, including 2.7 wt% NaCl, 0.5 wt% MgCl_2_, 0.07 wt% KCl, and 3.3 wt% Na_2_SO_4_ (Fig. 4h). The results indicated that NaCl significantly promoted H_2_O_2_ production, whereas replacing Cl^−^ with SO_4_^2−^ led to a pronounced decrease in yield, highlighting the critical role of Cl^−^ in enhancing the reaction. As an efficient hole scavenger, Cl^−^ can be oxidized by photogenerated holes, thereby suppressing the recombination of electron-hole pairs [53]. Concurrently, the generated Cl_2_ reacts with water to form HClO, which decomposes under light irradiation to produce O_2_ and H^+^, thereby facilitating the ORR pathway for H_2_O_2_ generation [54]. In contrast, the presence of K^+^ was found to inhibit H_2_O_2_ production. Notably, although the concentration of MgCl_2_ added was less than one-fifth of that of NaCl, its promoting effect on H_2_O_2_ generation was particularly pronounced.

Previous studies have shown that metal cations can modulate the surface charge distribution of catalysts, thereby influencing the separation and migration of photogenerated charge carriers and enhancing H_2_O_2_ production [55]. To further investigate this effect, the stable adsorption configurations of Na^+^, K^+^, Mg^2+^, and Cl^−^ ions on the OCCN surface were optimized using water as the solvent (dielectric constant = 78.54), and the corresponding adsorption energies were calculated. The results show that Cl^−^ can adsorb stably on the OCCN surface, while the adsorption of Na^+^, K^+^, and Mg^2+^ is relatively weak (Fig. S32). To further elucidate the electronic effects, Mulliken charge analysis was performed on OCCN after ion adsorption (Fig. S33). The results reveal that all ions can significantly modulate the surface charge distribution, thereby altering the Mulliken charge difference between adjacent heptazine units bridged by oxygen atoms and consequently tuning the inductive effect (Fig. S34). Notably, Mg^2+^ and Cl^−^ adsorbed near the cyano group sites exhibit the most pronounced enhancement of the inductive effect. Importantly, Mg^2+^ and Cl^−^ act synergistically to further amplify this effect.

### Mechanism study on the ORR of OCCN

**Figure 5.**
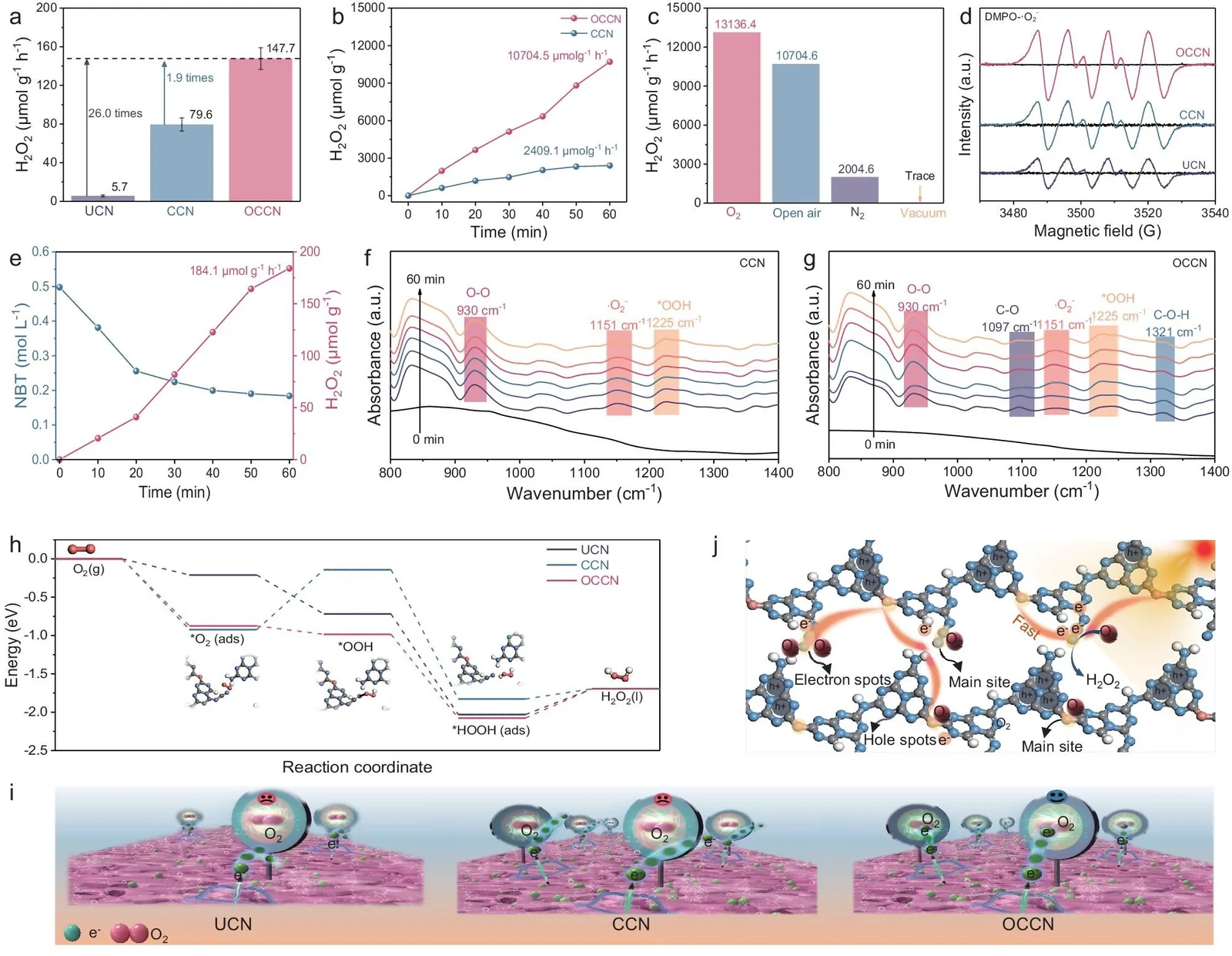
(a) Photocatalytic H_2_O_2_ production without IPA. (b) Time-dependent H_2_O_2_ photogeneration without aeration. (c) The H_2_O_2_ production of OCCN under different conditions (O_2_-saturated, open air, N_2_-saturated, vacuum). (d) EPR spectra for DMPO-⋅O_2_^−^ signal. (e) NBT consumption and H_2_O_2_ production of OCCN (O_2_-saturated, 0.5 M NBT). *In-situ* DRIFTS spectra acquired over different illumination times for (f) CCN and (g) OCCN photocatalytic system. (h) Free energy diagrams for O_2_ to H_2_O_2_ on the UCN, CCN, and OCCN through indirect two-electron ORR pathway. (i) Diagram of light-driven ORR on OCCN: the adsorption site aligned with the hot electron accumulation site. (j) Function of ether bonds in OCCN for photosynthesis of H_2_O_2_.

A series of control experiments were conducted to gain further insight into the reaction mechanism of H_2_O_2_ generation. Photocatalytic H_2_O_2_ production of the samples in the absence of IPA is presented in Fig. 5a. Without IPA, the H_2_O_2_ yields of UCN and CCN were 5.7 and 79.6 μmol g^−1^ h^−1^, respectively, whereas OCCN achieved a yield of 147.7 μmol g^−1^ h^−1^, corresponding to 26.0- and 1.9-fold enhancements compared to UCN and CCN. In pure water, OCCN exhibited the highest AQE of 5.9% at 420 nm, along with a solar-to-chemical conversion (SCC) efficiency of 0.69%, surpassing the performance levels of most previously reported photocatalytic systems (Table S6).

However, all samples exhibited a significant decrease in H_2_O_2_ yield in the absence of IPA. The H_2_O_2_ yields of UCN, CCN and OCCN were only 2.9%, 1.8% and 1.1% of those with IPA, respectively. As an efficient hole scavenger, IPA consumed photogenerated holes and suppressed electron–hole recombination, thereby promoting the participation of photogenerated electrons in ORR to produce H_2_O_2_. Notably, OCCN exhibits the most pronounced decrease in activity, indicating that it possesses the most efficient charge separation and transport properties, consistent with the EIS and transient photocurrent results (Fig. S11 and Fig. 2g). To further investigate the effect of hole scavengers on the H_2_O_2_ production performance over OCCN, several common hole scavengers [methanol (MeOH), ethanol (EtOH), and triethanolamine (TEOA), 10 vol%] were evaluated in control experiments. As shown in Fig. S35, all scavengers significantly enhanced the photocatalytic H_2_O_2_ generation rate of OCCN, with trends consistent with those observed for IPA. These results confirm that the enhancement in H_2_O_2_ yield primarily arises from efficient hole scavenging, representing a general effect rather than one specific to IPA.

Under nonaerated conditions, the H_2_O_2_ yields of CCN and OCCN dropped to 10704.5 and 2409.1 μmol g^−1^ h^−1^, respectively, corresponding to 52.0% and 81.5% of their respective yields under O_2_-saturation conditions (Fig. 5b). These results suggest that OCCN possesses a superior ability to adsorb and reduce O_2_ compared to CCN. To further evaluate the influence of atmospheric conditions, the photocatalytic performance of OCCN was examined under O_2_-saturated, open-air, N_2_-saturated, and vacuum environments (Fig. 5c). The H_2_O_2_ yields of OCCN under N_2_-saturated condition sharply decreased to 2004.6 μmol g^−1^ h^−1^, highlighting the crucial role of oxygen in the photocatalytic process. Almost no H_2_O_2_ was detected under vacuum conditions, confirming that the oxygen atoms in the produced H_2_O_2_ originate from adsorbed molecular oxygen. These findings collectively demonstrate that OCCN generates H_2_O_2_ through a two-electron ORR pathway:


(1) \begin{eqnarray*}
{{\mathrm{O}}}_2 + 2{{\mathrm{e}}}^ - + 2{{\mathrm{H}}}^ + \to {{\mathrm{H}}}_2{{\mathrm{O}}}_2,
\end{eqnarray*}

(2) \begin{eqnarray*}
{{\mathrm{O}}}_2 + {{\mathrm{e}}}^ - \to \cdot {{\mathrm{O}}}_2^ - ,
\end{eqnarray*}

(3) \begin{eqnarray*}
\cdot {{\mathrm{O}}_2^ -} + {{\mathrm{e}}}^ - + 2{{\mathrm{H}}}^ + \to {{\mathrm{H}}}_2{{\mathrm{O}}}_2.
\end{eqnarray*}

Considering that H_2_O_2_ production via the ORR mechanism can proceed through either a direct two-electron pathway [Equation (1)] or an indirect two-electron pathway involving reactive oxygen intermediates [Equations (2) and (3)], further investigations were carried out to elucidate the dominant reaction route. The intermediate superoxide radical (⋅O_2_^−^), a hallmark of the indirect two-electron ORR pathway, was detected using EPR spectroscopy with 5,5-dimethyl-1-pyrroline *N*-oxide (DMPO) as the spin-trapping agent [15]. As depicted in Fig. 5d, OCCN exhibited a characteristic six-line EPR signal corresponding to the DMPO-⋅O_2_^−^ adduct, with the highest peak intensity among the tested samples. This observation suggests that the reversed inductive effect in OCCN favors both oxygen adsorption and its subsequent activation by energetic hot electrons to form ⋅O_2_^−^. To further quantify the contribution of the indirect two-electron ORR to H_2_O_2_ production, nitroblue tetrazolium (NBT), a known ⋅O_2_^−^ scavenger, was introduced (Fig. 5e). Upon the addition of NBT, the H_2_O_2_ yield of OCCN dramatically decreased to 184.1 μmol g^−1^ h^−1^, only 1.4% of the yield observed without NBT. These results provide strong evidence that H_2_O_2_ production over OCCN predominantly follows an indirect two-electron ORR mechanism, with ⋅O_2_^−^ as the key intermediate.

*In-situ* diffuse reflectance infrared Fourier transform spectroscopy (DRIFTS) was employed to monitor real-time intermediate species during photocatalytic H_2_O_2_ generation over CCN and OCCN. New peaks appeared at 930, 1151 and 1225 cm^−1^ under illumination (Fig. 5f and g), corresponding to the vibration modes of O–O, ⋅O_2_^−^ and ⋅OOH, respectively [50,56,57]. These signals confirmed the involvement of key intermediates associated with the indirect two-electron ORR pathway. Interestingly, in the case of CCN, the intensities of the O–O and ⋅O_2_^−^ peaks remained relatively constant throughout illumination, suggesting that these intermediates are stable and not readily reduced or desorbed. In contrast, for OCCN, the ⋅O_2_^−^ peak intensity was significantly lower than that of ·OOH after illumination, indicating a more efficient conversion of ⋅O_2_^−^ to ⋅OOH. Additionally, a gradual increase in the ⋅O_2_^−^ peak over time reflected the accumulation of this intermediate on the catalyst surface. Notably, under light exposure, OCCN also exhibited vibrational peaks at 1097 cm^−1^ and 1321 cm^−1^, assigned to C–O and C–O–H stretching vibrations, with dynamic intensity changes. These features indicate that the ether bond functions as an active site for ORR, facilitating O_2_ adsorption and activation, and promoting H_2_O_2_ formation through a cascade of reactive intermediates.

Density functional theory calculations were performed to elucidate the role of inductive effect engineering, via ether bonds and cyano groups, on the ORR performance. Optimized adsorption configurations of O_2_ on various sites of UCN, CCN, and OCCN are shown in Figs S36–S38. On UCN, O_2_ exhibited weak and nearly uniform adsorption across different surface sites, attributed to the homogeneous *π*-electron distribution and the negligible inductive effect. In contrast, CCN and OCCN displayed pronounced differences in O_2_ adsorption energy (*E*_abs_) between Type I sites (adjacent to bridging nitrogen or oxygen atoms) and Type II sites (cyano groups), suggesting that cyano groups serve as preferential adsorption sites due to their strong electron-withdrawing nature. Notably, the incorporation of ether bonds in OCCN enhanced O_2_ adsorption at the bridging sites while slightly weakening it at the cyano-based sites. This trend aligns well with the O_2_-TPD results (Fig. 2j), and can be ascribed to the redistribution of local electron density induced by the combined inductive effects of cyano and ether functionalities.

To further assess the thermodynamic feasibility of the ORR process, indirect two-electron ORR pathways were simulated for UCN, CCN, and OCCN (Fig. 5h, Figs S39 and S40). The Gibbs free energy (Δ*G*) for *O_2_ formation on UCN was calculated to be −0.21 eV, while significantly lower values of −0.92 and −0.87 eV were found on the cyano sites of CCN and OCCN, respectively. These results confirm that cyano groups enhance O_2_ activation by modulating the local electron distribution through a strong inductive effect. However, the introduction of highly electronegative oxygen atoms via ether linkages in OCCN attenuates O_2_ adsorption at the cyano sites, fine-tuning the adsorption strength and reactivity. Interestingly, the Δ*G* for the formation of *OOH intermediates (*O_2_ + H^+^ + e^–^ → *OOH) on CCN was calculated to be 0.78 eV, indicating a substantial kinetic barrier. This suggests that, despite cyano groups enhancing O_2_ adsorption, they are inefficient in accepting hot electrons, making the *OOH formation step rate-limiting. In contrast, OCCN exhibited a much lower Δ*G* of −0.11 eV for *OOH formation. The ether linkages in OCCN facilitate electron redistribution, enhancing the capacity of cyano groups to accept hot electrons. This promotes efficient electron accumulation at the active sites and significantly accelerates the formation of key *OOH intermediates.

In addition, *in-situ* DRIFTS results (Fig. 5g) suggested that ether bonds may participate in the ORR process. Accordingly, the indirect two-electron ORR pathway at the ether bond sites of OCCN was simulated (Fig. S41). The calculated Δ*G* values for the formation of *O_2_ and *OOH near the ether bond were −0.24 and −0.55 eV, respectively. This thermodynamic advantage further confirms that the cyano group serves as the primary active site for ORR, while the ether bond plays only a secondary role. As such, cyano group acts as the dominant site for O_2_ adsorption and activation, whereas ether linkages regulate the inductive effect, but collectively facilitating the overall ORR process.

Besides, seawater ions can modulate the surface charge distribution of OCCN, thereby influencing O_2_ adsorption and the thermodynamics of ORR. We calculated the effects of Mg^2+^ and Cl^−^ on O_2_ adsorption under solvation conditions. As shown in Fig. S42, solvation slightly weakened O_2_ adsorption at the cyano sites. Cl^−^ promoted O_2_ adsorption at these sites, whereas Mg^2+^ exerts an inhibitory effect. However, when both Mg^2+^ and Cl^−^ are present, the O_2_ adsorption strength is enhanced. Furthermore, the thermodynamic effects of these ions on the ORR were investigated by simulating the indirect two-electron ORR pathway in the presence of both Mg^2+^ and Cl^−^ (Fig. S43). Under solvation conditions, the Δ*G* values for *O_2_ and *OOH formation were −0.87 and −0.21 eV, respectively. In the presence of Mg^2+^/Cl^−^, these values decrease to −0.90 and −0.26 eV, respectively, indicating that the synergistic effect of Mg^2+^ and Cl^−^ lowered the ORR energy barrier by optimizing the electronic structure, thereby promoting H_2_O_2_ generation.

Therefore, CCN benefits from enhanced charge dynamics and strong O_2_ adsorption due to the negative inductive effect, but its limited ability to accumulate hot electrons hinders ORR kinetics. The introduction of ether linkages in OCCN strategically modulates the electronic structure, strengthens BIEF, and improves photogenerated charge separation and transfer. More importantly, this structural modification enables cyano groups to function synergistically as both efficient O_2_ adsorption sites and effective hot electron acceptors (Fig. 5i and j), thereby improving both the selectivity and efficiency of the two-electron ORR to H_2_O_2_ and significantly enhancing overall energy utilization.

## CONCLUSIONS

In this study, we systematically uncovered the root causes of the suboptimal photocatalytic H_2_O_2_ production performance of CCN. Although the exposed cyano groups in CCN exert a negative inductive effect along the *π*-conjugated system, enhancing mass and energy transfer, they are ineffective at facilitating electron accumulation for O_2_ photocharging. By introducing ether bonds into the CCN framework, we not only improved photoexcited charge dynamics but also reversed the inductive effect in OCCN, enabling the cyano groups to act as efficient electron-accepting sites for the ORR. This tailored inductive effect also lowered the thermodynamic barrier for the formation of the key *OOH intermediate, further facilitating H_2_O_2_ generation. As a result, OCCN delivered significantly higher H_2_O_2_ yield and selectivity compared to CCN and CN, along with superior light-harvesting efficiency. Remarkably, OCCN exhibited an even greater H_2_O_2_ production rate of 16 039.8 μmol g^–1^ h^–1^ in natural seawater and maintained a stable yield of 110.4 μmol h^–1^ under continuous flow conditions. This work provides critical insights into identifying and modulating the hidden factors governing photocatalytic behavior in polymeric systems and advances practical strategies for solar-driven H_2_O_2_ synthesis, paving the way for the rational design of smart materials for solar fuels production.

## Supplementary Material

nwag284_Supplemental_File
