# Supplementary material for: Inductive effect reversal drives efficient photocharging of catalytic sites in polymeric catalysts for continuous-flow H2O2 synthesis
Source: Natl Sci Rev. 2026 May 14;13(15):nwag284. doi: 10.1093/nsr/nwag284 (PMC13563090; doi:10.1093/nsr/nwag284)
Supplement: Supplementary file 1 [file nwag284_supplemental_file.pdf]

## Supplementary Information

# Inductive effect reversal drives efficient photocharging of catalytic sites in polymeric catalysts for continuous-flow $H_2O_2$ synthesis

Yang Zhang[1], Ning Cao[2], Fengting He[3,*], Yuzhao Wu[4], Xili Shang[1,*], Chaocheng Zhao[2], Shaobin Wang[4], and Jinqiang Zhang[4,5,*]

[1]School of Chemical Engineering and Materials Science, Shandong University of Aeronautics, Binzhou 256600, P.R. China.

[2]State Key Laboratory of Petroleum Pollution Control, China University of Petroleum (East China), Qingdao 266580, P.R. China.

[3]Institute for Smart Materials & Engineering, Jinan University, Jinan 250022, P.R. China.

[4]School of Chemical Engineering, The University of Adelaide, Adelaide, SA 5005, Australia.

[5]School of Molecular Sciences, The University of Western Australia, WA 6009, Australia.

*Corresponding E-mails: fengtinghe1009@163.Com; xilishang@126.com; jinqiang.zhang@uwa.edu.au

## Experimental section

### Chemicals

All chemicals and solvents were purchased from Shanghai Aladdin Biochemical Technology Co. Ltd. and used without further purification. Deionized water with a resistivity of 18.25 MΩ·cm was used in all experiments. Natural seawater was collected from the Yellow Sea, Qingdao, China, and filtered through 0.22 μm membranes prior to use.

### Synthesis of U400 and UCN

10 g of urea was placed in a covered crucible and heated in a muffle furnace at 400 °C for 2 h, with a heating rate of 5 °C $min^{-1}$. After natural cooling to room temperature, the resulting product was collected and denoted as U400. The synthesis of UCN followed the same procedure, except that the heating temperature was increased to 550 °C.

### Synthesis of OCCN and CCN

OCCN and CCN photocatalysts were synthesized via a molten salt calcination method. Specifically, 0.6 g of U400 was thoroughly mixed with 3.6 g of NaCl and 2.6 g of KCl, and the mixture was placed into a lidded square porcelain boat. The system was then heated to 550 °C for 3 hours under a nitrogen atmosphere, with a heating rate of 5 °C $min^{-1}$. After naturally cooling to ambient temperature, the solid product was repeatedly washed with boiling deionized water, and subsequently dried at 60 °C for 2 hours to yield OCCN. The synthesis of CCN was carried out using the same protocol, with UCN used in place of U400.

### Performance evaluation in photosynthesis of $H_2O_2$

Photocatalytic reaction was performed by dispersing 50 mg of catalyst in 100 mL of an aqueous solution containing 10 vol% isopropanol in a double-jacketed cylindrical reactor, maintained at 25 °C using a circulating thermostat. Illumination was provided by a 300 W xenon lamp equipped with a 420 nm cut-off filter, positioned 15 cm above the liquid surface. Prior to irradiation, the solution was purged with oxygen at a flow rate of 300 mL·$min^{-1}$ in the dark. At 10-minute intervals, 3 mL aliquots were withdrawn from the reaction mixture, filtered through a 0.22 μm membrane, and analyzed. For experiments involving natural seawater, deionized water was replaced with

untreated seawater under otherwise identical conditions. The concentration of $H_2O_2$ was quantified using iodometric titration and UV–vis spectrophotometry[1]. 3 mL of filtered reaction solution was mixed with 1 mL of potassium hydrogen phthalate (0.1 M), 1 mL of potassium iodide (0.4 M), and reacted for 30 min in the absence of light irradiation. The concentration of $H_2O_2$ was determined by monitoring the absorbance at 350 nm using a UV-vis spectrometer.

**Apparent quantum efficiency (AQE) measurement**

AQE for photocatalytic $H_2O_2$ production was evaluated using 50 mg of catalyst dispersed in 100 mL of aqueous solution containing 10 vol% isopropanol (IPA). Monochromatic light sources at wavelengths of 395 nm and 420 nm were used for irradiation over 60 min, and their intensities were measured using an optical power meter (CEL-NP2000-2). For AQE measurements in pure water, 100 mg of catalyst was dispersed in 100 mL of deionized water under otherwise identical conditions.

AQE was calculated according to Supplementary equation (1):

$$\text{AQE} = \frac{2 \times \text{Number of produced } H_2O_2 \text{ molecules}}{\text{Number of incident photons}} \times 100\% \quad (1)$$

The number of incident photons was determined using Supplementary equation (2):

$$N_{\text{incident}} = \frac{ISt\lambda}{hc} \quad (2)$$

Where $I$ is the light intensity (W $m^{-2}$), $S$ represents the irradiation area ($m^2$), $t$ is the irradiation time (s), $\lambda$ represents the wavelength of the incident light (nm), $h$ signifies Planck constant ($6.63 \times 10^{-34}$ J s), and $c$ is the speed of light ($3\times10^8$ m $s^{-1}$).

**Solar-to-chemical conversion (SCC) Efficiency measurements**

SCC efficiency was measured using 100 mg of catalyst dispersed in 100 mL of $O_2$-saturation pure water under AM 1.5G light irradiation (100 mW $cm^{-2}$). The reaction system was continuously stirred at 400 rpm throughout the experiment, and the temperature was maintained at 25 ºC using a circulating water bath. The SCC efficiency was calculated via the following equation:

$$\text{SCC} = \frac{\Delta G_{H_2O_2} \times H_2O_2 \text{formed}}{I \times S \times T} \times 100\% \quad (3)$$

Where $\Delta G_{H_2O_2}$, I, S, and T is the free energy for $H_2O_2$ generation (117 kJ $mol^{-1}$), light intensity, irradiated area (1 $cm^2$), and irradiation time (s), respectively.

**$\cdot O_2^-$ Measurements**

The contribution of $\cdot O_2^-$ to $H_2O_2$ production was examined using nitroblue tetrazolium (NBT) as a probe. NBT exhibits a maximum absorption peak at 259 nm and reacts with $\cdot O_2^-$ to form an insoluble blue-violet formazan. For the test, 50 mg of catalyst was dispersed in a 0.5 mM NBT solution containing 10 vol% IPA. The system was saturated with oxygen under dark conditions for 30 min, followed by visible light irradiation using a 300 W xenon lamp equipped with a 420 nm cut-off filter. During the reaction, 1 mL aliquots were taken every 10 min, filtered through a 0.22 μm membrane, and analyzed using a UV–Vis spectrophotometer to monitor the NBT transformation.

**Photocatalytic $H_2O_2$ continuous production**

100 mg of photocatalyst (OCCN) was ultrasonically dispersed and immobilized on the bottom of a glass reactor with an area of approximately 28.3 $cm^2$. Subsequently, 100 mL of an aqueous IPA solution (10 vol%) was added. A dual-channel peristaltic pump system was used to introduce fresh $H_2O$/IPA (10 vol% IPA) into the reactor and simultaneously transfer the reaction mixture to a separate $H_2O_2$ collection vessel at a flow rate of about 5 mL $h^{-1}$. Prior to illumination, both the reaction and supplemental solutions were bubbled with oxygen for 30 min. A 300 W xenon lamp equipped with a 420 nm cutoff filter was used as the light source, while oxygen was continuously supplied at 100 mL $min^{-1}$. The concentration of $H_2O_2$ in the collection vessel was measured hourly to evaluate long-term performance.

**Characterization**

Scanning electron microscopy (SEM, FEI S4800) and transmission electron microscopy (TEM, JEOL 2100) were employed to investigate the morphology of the samples. Powder X-ray diffraction (PXRD) patterns were collected using a Bruker D8 Advance diffractometer with Cu-Kα radiation (λ = 1.5406 Å). Fourier transform infrared spectra (FT-IR) spectra were recorded on a Nicolet Nexus 670 spectrometer. X-ray photoelectron spectroscopy (XPS) was performed on an ESCALAB 250Xi instrument to analyze surface chemical states. Solid-state $^{13}C$ nuclear magnetic resonance (NMR) spectroscopy was conducted using a Bruker AVANCE NEO 600 MHz system. Steady-state photoluminescence (PL) spectra were acquired on a Hitachi F-4600 spectrofluorometer with an excitation wavelength of 320 nm. UV–Vis diffuse reflectance spectra

(DRS) were measured on a TU-1901 spectrophotometer using $BaSO_4$ as the reference. Electron paramagnetic resonance (EPR) spectra were recorded on a Bruker A300 spectrometer. Atomic force microscopy (AFM) and surface potential measurements were carried out using a Bruker Dimension Icon system equipped with a Kelvin probe. Zeta potential measurements were performed using a Malvern Zetasizer Nano ZS. Oxygen temperature-programmed desorption ($O_2$-TPD) test was performed on a BELCAT-B analyzer (Japan).

**Built-in electric field measurement**

Built-in electric field of materials was estimated using the Kanata model equation as referenced[2].

$$E = \left(\frac{-2V_S\,\rho}{\varepsilon\varepsilon_0}\right)^{\frac{1}{2}} \tag{4}$$

E – Value of built-in electric field

$V_S$ – Surface potential, V

ρ – Surface charge density, $C{\cdot}m^{-2}$

ε – Low-frequency dielectric constant

$\varepsilon_0$ – Vacuum dielectric constant, $8.854\times10^{-12}$ $F{\cdot}m^{-1}$

Considering that the polymeric materials have similar structures and compositions, ε can be approximated as constant, and $\varepsilon_0$ is also a constant. As a result, the equation (5) indicates that built-in electric field is mainly determined by the surface voltage and the surface charge density. $V_S$ can be detected by AFM with KPFM model. According to the Gouy-Chapman model, the ρ can be tested and calculated by the zeta potential.

$$\rho = \sqrt{8kT\varepsilon\varepsilon_0 n}\sinh\left(\frac{ze_0V_i}{2kT}\right) \tag{5}$$

$V_i$ – Zeta potential, V

k – Boltzmann constant, $1.381\times10^{-23}$ $J{\cdot}K^{-1}$

T – Absolute temperature, K

n – Number of electrolytes per unit volume, $m^{-3}$

$e_0$ – Electron charge, $1.602\times10^{-19}$ C

z – Electrolyte valence

Based on these relationships, the built-in electric field can be approximated as a function of the surface potential $V_S$ and Zeta potential.

$$E = A\left(V_S\sinh\left(\frac{ze_0V_i}{2kT}\right)\right)^{\frac{1}{2}} \tag{6}$$

A – A combined constant

**In-situ DRIFTS measurement**

In-situ diffuse reflectance infrared Fourier transform spectroscopy (DRIFTS) was conducted using a Bruker IFS 125HR instrument. A 300 W xenon lamp equipped with a 420 nm cutoff filter served as the light source. Prior to measurement, the sample was dried at 90 °C for 60 mins and then loaded into an in-situ IR cell. $O_2$, IPA vapor and $H_2O$ vapor were introduced into the cell. After purging with saturated vapor in the dark for 30 mins to establish a baseline, spectra were recorded at 10-min intervals.

**Electrochemical rotating disk electrode (RDE) measurement**

RDE measurements were carried out on a CHI760e electrochemical workstation using a standard three-electrode configuration. A rotating disk electrode (glassy carbon electrode with an area of 0.1256 $cm^2$), a saturated calomel electrode (SCE), and a platinum wire were used as the working, reference, and counter electrodes, respectively. The working electrode was prepared as follows: 2 mg of catalyst was dispersed in a mixture containing 700 μL ethanol and 100 μL Nafion solution (5 wt%), followed by sonication for 60 min to form a homogeneous ink. Subsequently, 30 μL of the suspension was drop-cast onto the glassy carbon electrode and dried at room temperature. Linear sweep voltammograms (LSVs) were measured in $O_2$-saturated 0.1 M KOH aqueous solution. The scan rate was set to 5 mV/s, and the rotating speed of the working electrode was 400, 625, 900, 1225 and 1600 rpm, respectively. The average transfer number of electrons (n) was calculated based on the following Koutecky-Levich equation:

$$\frac{1}{J}=\frac{1}{J_L}+\frac{1}{J_K}=\frac{1}{B\omega^{1/2}}+\frac{1}{J_K} \tag{7}$$

$$B=0.62nFC_0D_0^{2/3}V^{-1/6} \tag{8}$$

Where J is the measured current density, $J_K$ refers to the kinetic current density, $J_L$ is the limiting diffusion current density, n is the transfer electron number, F represents the Faraday constant (96485 C $mol^{-1}$), and $C_0$ is saturated oxygen concentration in electrolyte ($1.26 \times 10^{-3}$ M). $D_0$ is the diffusion coefficient of $O_2$ ($1.93 \times 10^{-5}$ $cm^2$ $s^{-1}$), V is the kinetic viscosity of the electrolyte (0.0109 $cm^2$ $s^{-1}$), and ω is the rotation rate (rad·$s^{-1}$) of the electrode.

**Electrochemical rotating ring-disk electrode (RRDE) measurement**

The number of transferred electrons (n) and $H_2O_2$ selectivity evaluated using a RRDE system in a standard three-electrode configuration. A glassy carbon disk (area of 0.1256 $cm^2$) served as the working electrode, with an Ag/AgCl reference electrode and a platinum counter electrode. The electrolyte was an $O_2$-saturated 0.1 M KOH aqueous solution. The working electrode was prepared by dispersing 2 mg of catalyst in 800 μL of ethanol and 5 μL of Nafion solution, followed by sonication for 30 mins. A 15 μL aliquot of the suspension was drop-cast onto the RRDE and dried at room temperature. Linear sweep voltammetry (LSV) was performed at a scan rate of 5 mV/s with the electrode rotating at 1600 rpm.

The number of transferred electrons (*n*) was calculated based on the Supplementary equation (9):

$$n = \frac{4I_d}{I_d + I_r/N} \tag{9}$$

The selectivity of $H_2O_2$ was calculated according to the Supplementary equation (10):

$$\text{Selectivity (\%)} = 200 \times \frac{I_r/N}{I_d + I_r/N} \tag{10}$$

Where $I_r$ is the ring current, $I_d$ is the disc current, and $N$ is the collection efficiency ($N = 0.4$).

**Photoelectrochemical measurements**

Photoelectrochemical measurements, including photocurrent response, Mott-Schottky plots, and electrochemical impedance spectra, were carried out using an electrochemical workstation (CHI-660 B) within a three-electrode cell configuration. A 0.1 M $Na_2SO_4$ aqueous solution served as the electrolyte, with a Pt plate as the counter electrode, an Ag/AgCl electrode as the reference electrode, and the sample/fluorine-doped tin oxide glass with an active area of 1 $cm^2$ prepared as the working electrode. Photocurrent was recorded under a 300 W xenon lamp illumination, and electrochemical impedance spectra was obtained with the frequency ranging from 0.01 Hz to 1000 kHz. Mott-Schottky plots were acquired at a frequency of 1000 Hz.

**Theoretical-calculation details**

Density functional theory (DFT) calculations were performed using the DMol3 package within the Materials Studio framework[3,4]. Generalized gradient approximation (GGA) with the Perdew-Burke-Ernzerhof (PBE) was employed for the main calculations, with the Grimme method applied for DFT-D correction. The Monkhorst-Pack grid with a grid number set at 3×3×1 was used for k-point sampling. Total energy calculations were performed using the self-consistent iteration (SCF)

method, and a 0.005 Ha smearing was applied. Convergence criteria for structural optimization and energy calculations were set as: a) $1\times10^{-5}$ Hartree energy tolerance; b) $1\times10^{-6}$ Hartree SCF tolerance; c) $5\times10^{-3}$ Å maximum displacement tolerance; and d) $2\times10^{-3}$ Hartree/Å maximum force tolerance. A substantial vacuum space of 15 Å was introduced to eliminate interlayer interactions. Water, with a dielectric constant of 78.54, was used as the solvent environment. The adsorption energy ($E_{ads}$) was calculated to evaluate the stability of $K^+$, $Na^+$, $Mg^{2+}$, $Cl^-$ or $O_2$ adsorbed on the OCCN surface according to the following equation:

$$E_{ads} = E_{total} - E_{sub} - E_n \tag{11}$$

The change of Gibbs free energy ($\Delta G$) was defined as the Supplementary equation (5):

$$\Delta G = \Delta E + \Delta E_{ZPE} - T\Delta S \tag{12}$$

Where $\Delta E$, $\Delta E_{ZPE}$ and $\Delta S$ represent the changes in total energy, zero-point energy, and entropy, respectively. The zero-point energy and entropy can be obtained from vibrational frequency computation at $T = 298.15$ K.

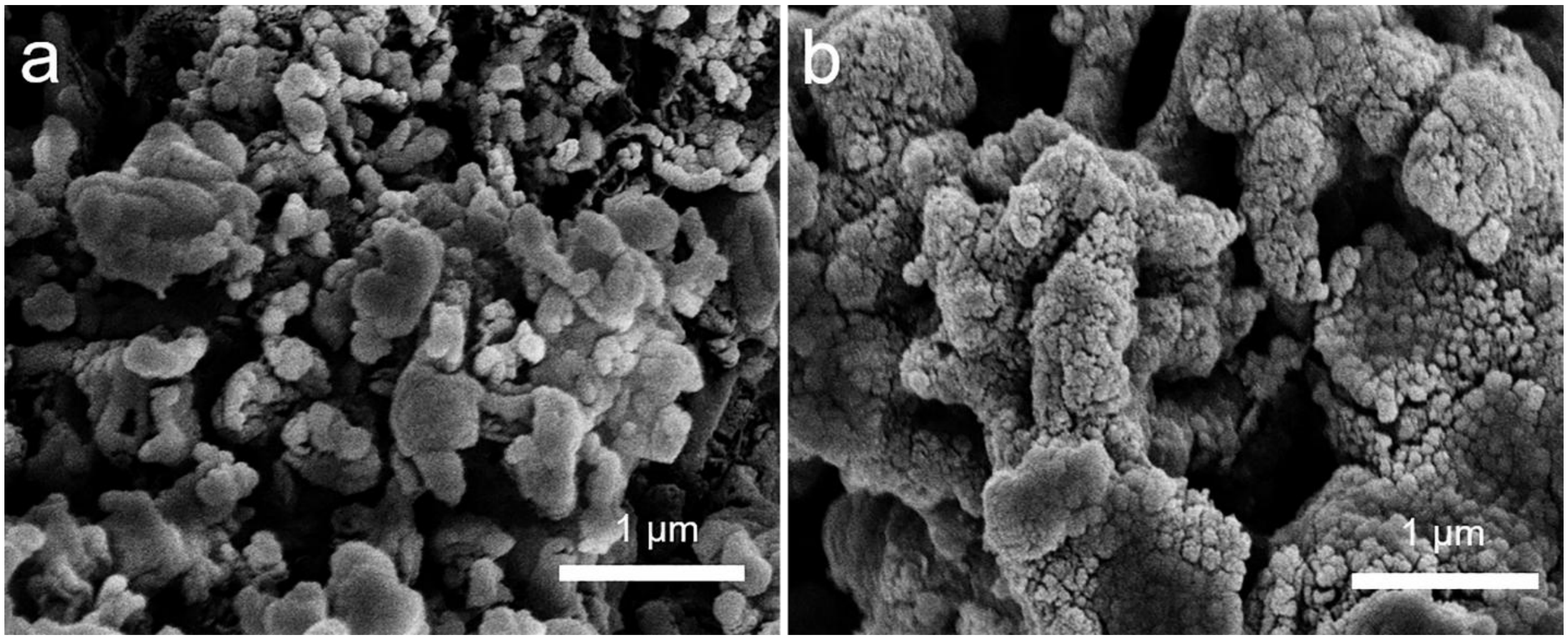


**Figure S1.** SEM images of (a) UCN and (b) CCN.

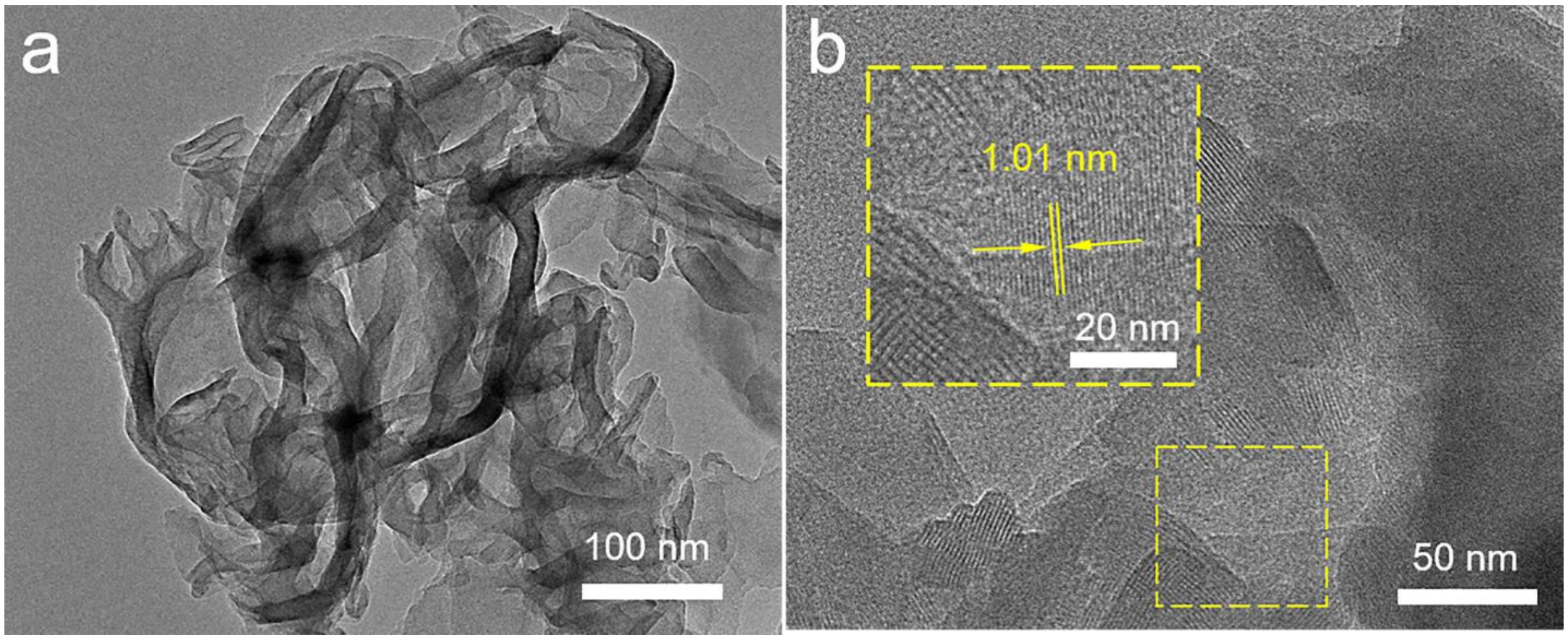


**Figure 2.** TEM images of (a) UCN and (b) CCN.

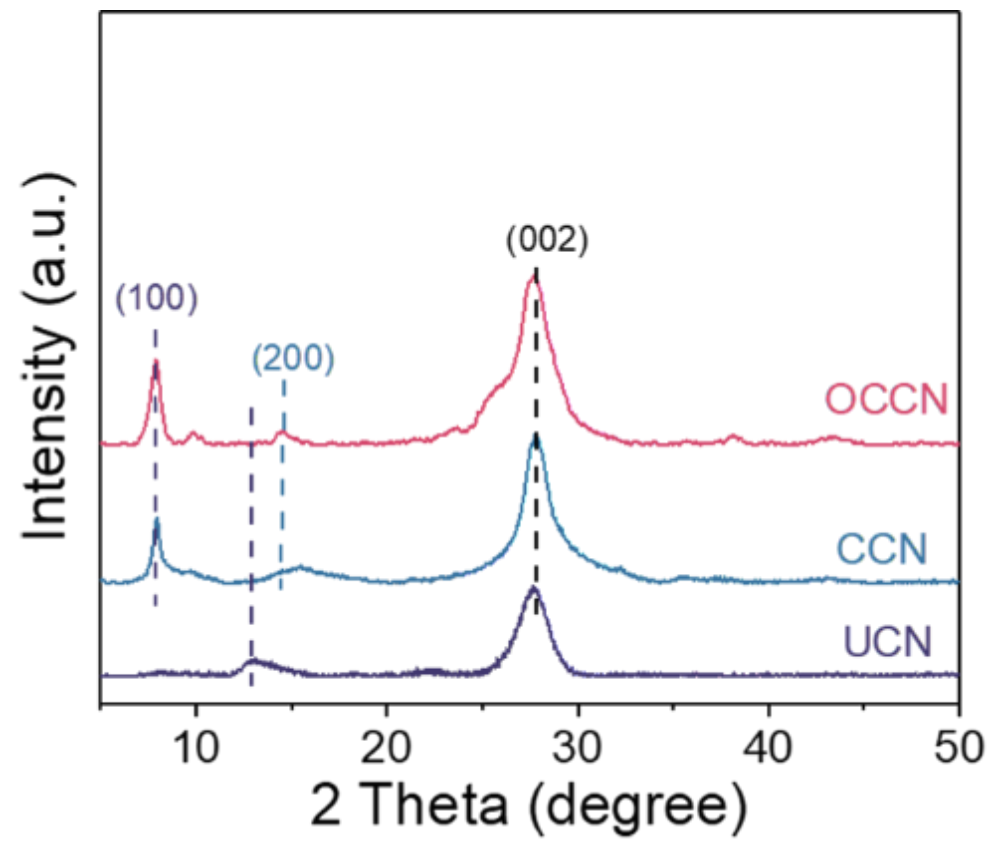


**Figure S3.** PXRD patterns of UCN, CCN, and OCCN.

The crystalline structure of the materials was characterized using powder X-ray diffraction (PXRD). UCN displayed two characteristic diffraction peaks at 27.8° and 13.1°, corresponding to the (002) interlayer stacking and the in-plane structural periodicity of the (100) plane, respectively. In contrast, the (100) peak of CCN shifted down to 8.0°, suggesting that the molten salt treatment effectively restructured the UCN into a more ordered crystalline phase[5].

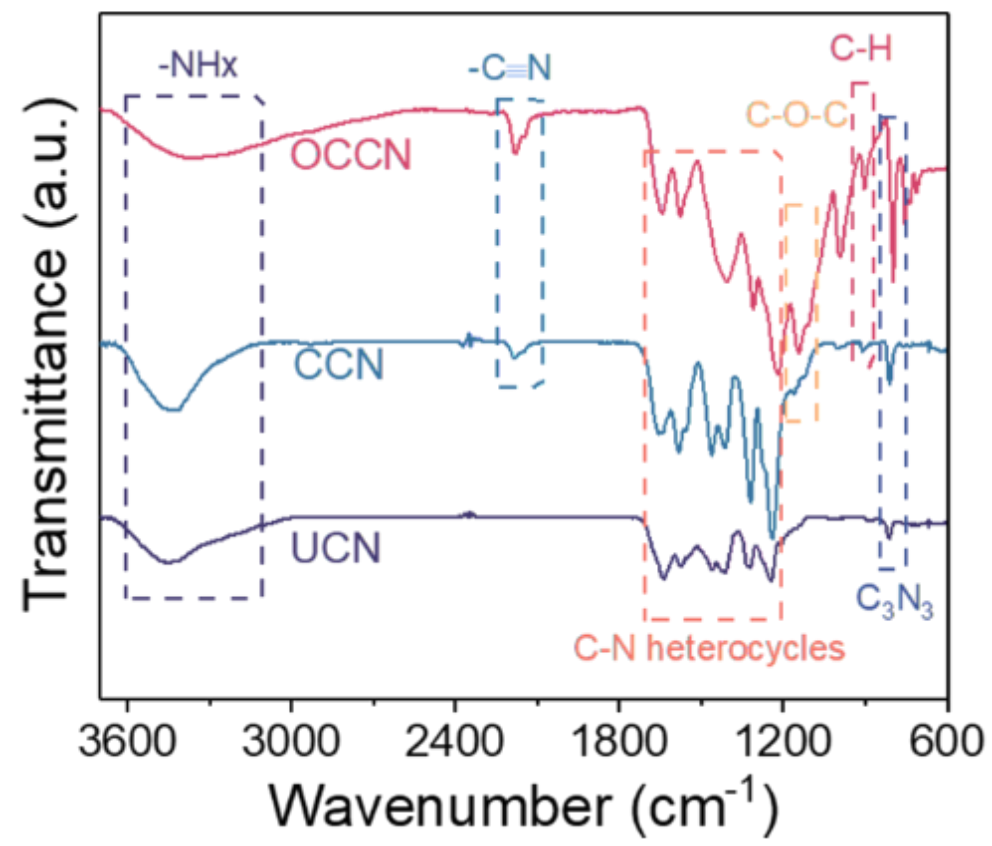


**Figure S4.** FT-IR spectra of UCN, CCN, and OCCN.

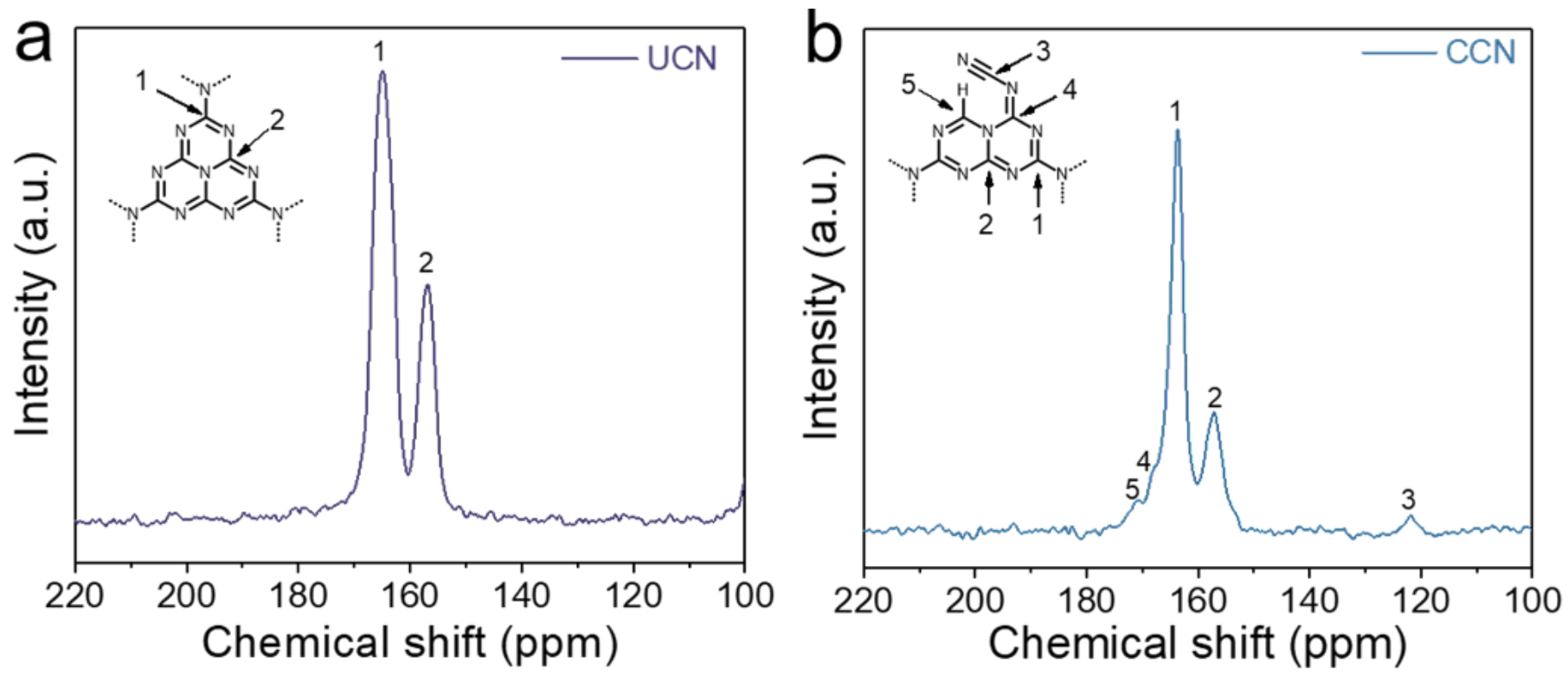


**Figure S5.** $^{13}C$ NMR spectra of (a) UCN and (b) CCN.

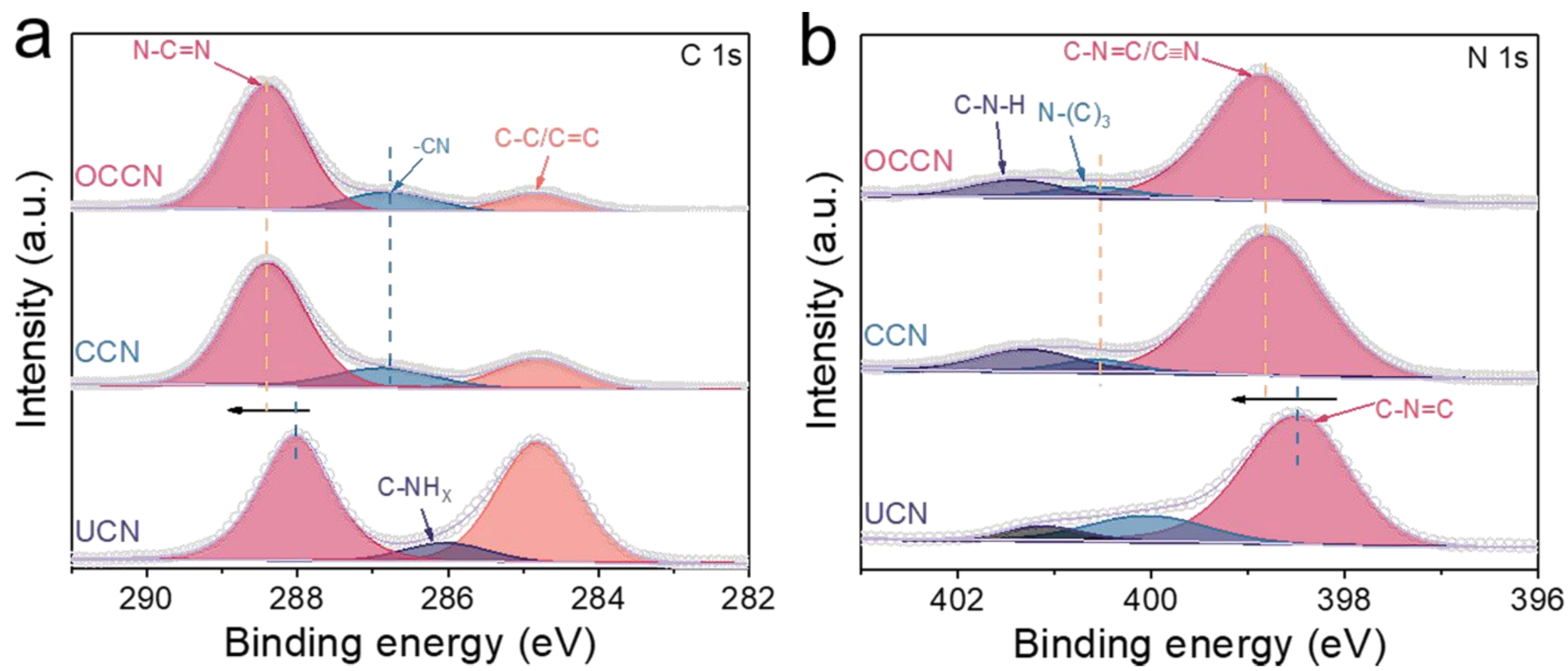


**Figure S6.** XPS spectra for (a) C 1s and (b) N 1s.

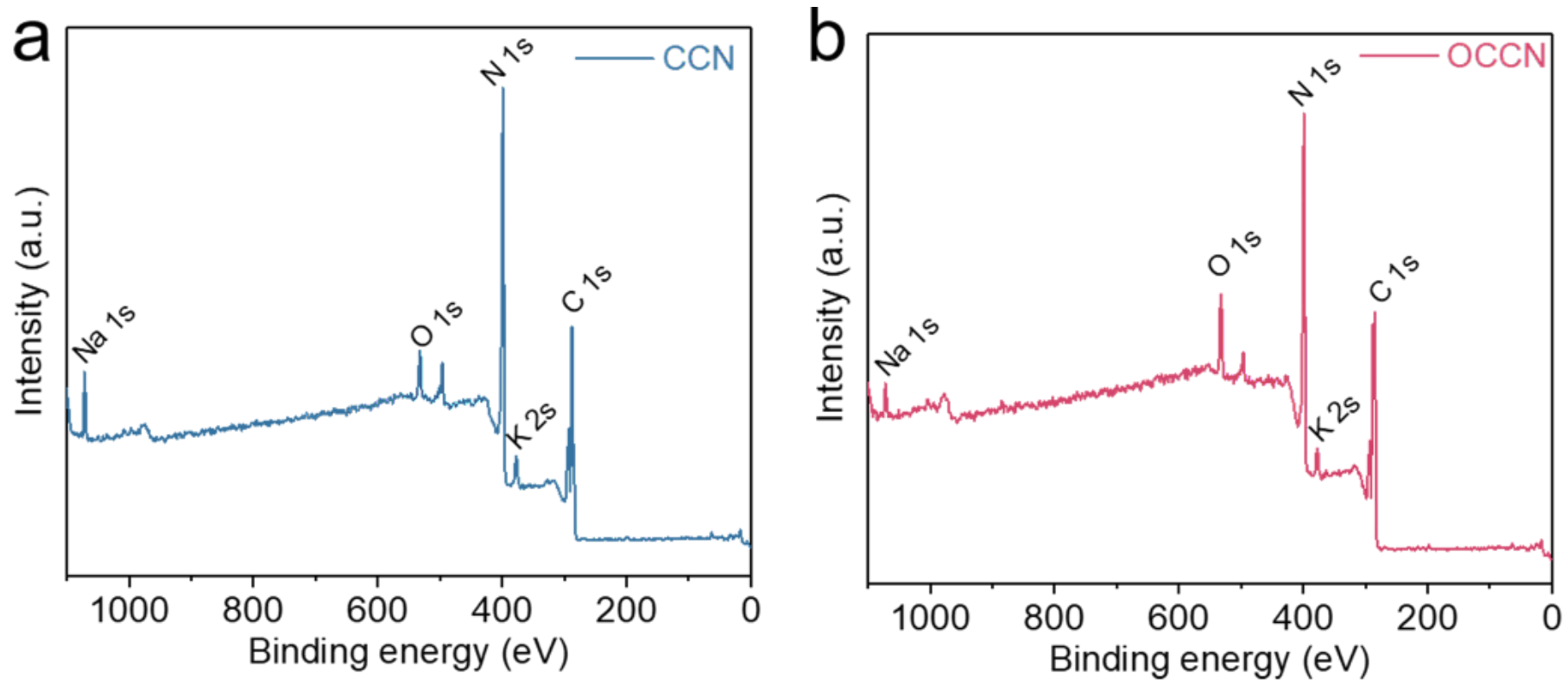


**Figure S7.** XPS Survey spectra of (a) CCN and (b) OCCN.

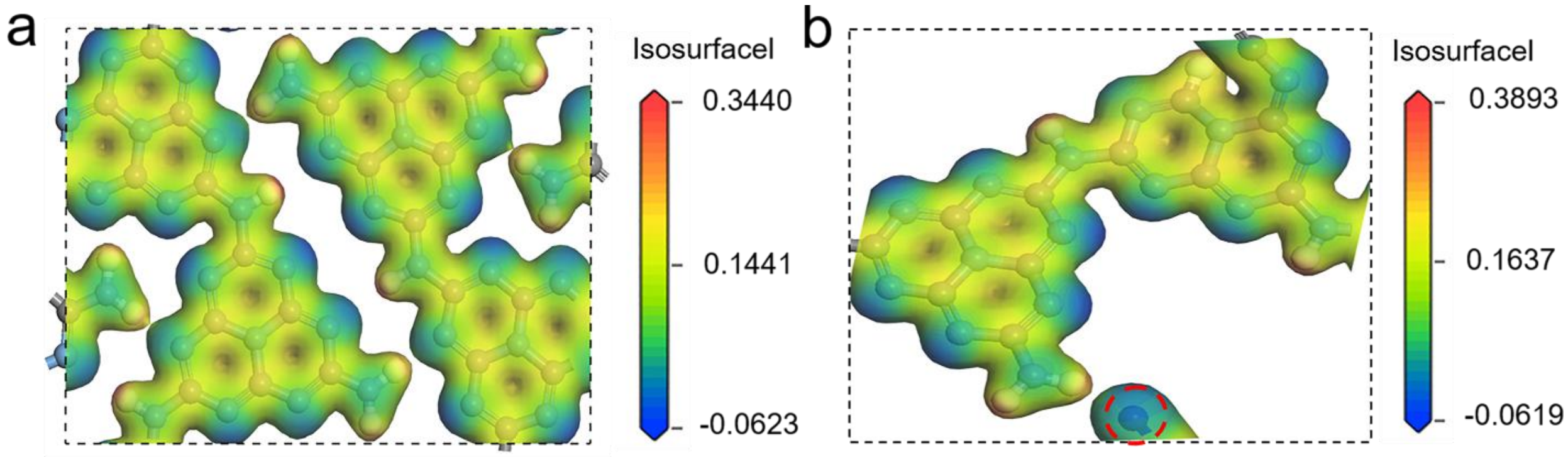


**Figure S8.** Electrostatic potential distribution of (a) UCN and (b) CCN.

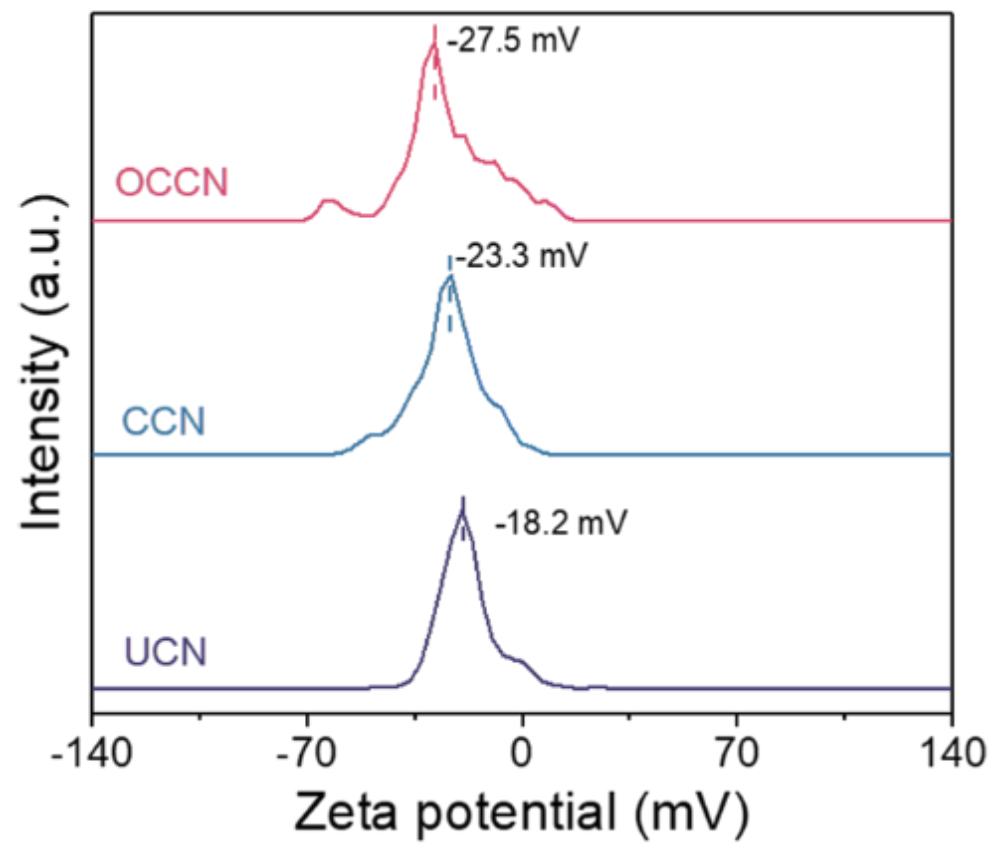


**Figure S9.** Zeta potentials of UCN, CCN and OCCN in deionized water.

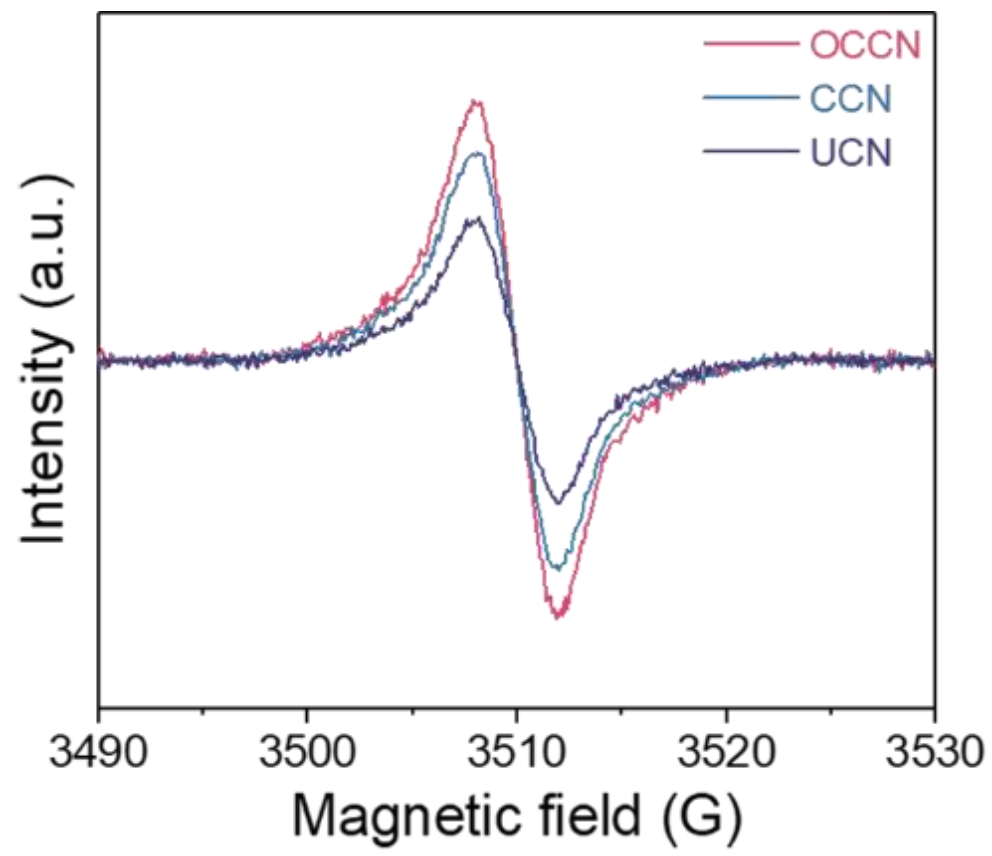


**Figure S10.** EPR spectra of UCN, CCN, and OCCN.

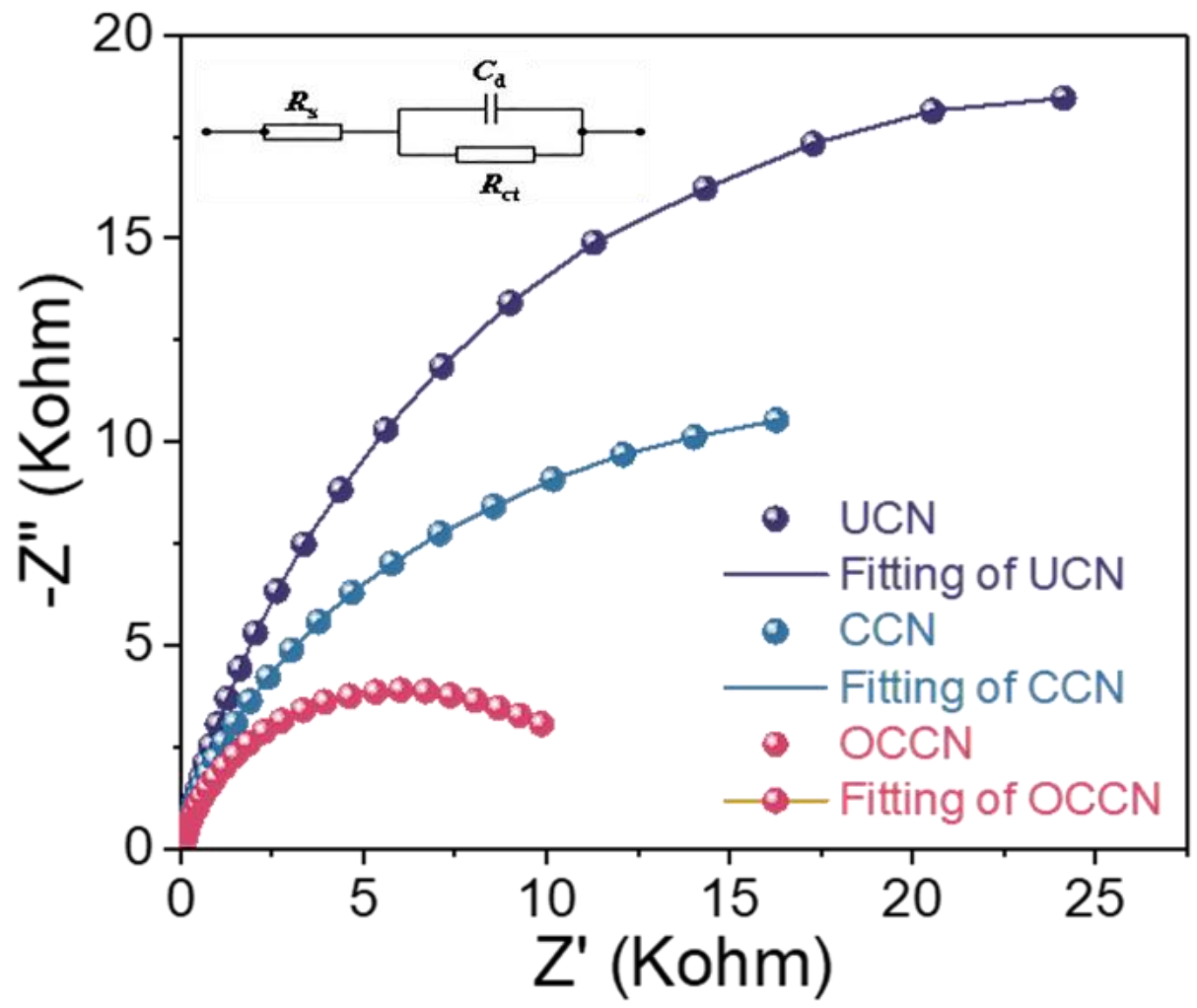


**Figure S11.** EIS spectra of UCN, CCN, and OCCN.

The high and low frequency semicircles observed in the Nyquist diagram are attributed to the solution resistance ($R_s$) and charge transfer resistance ($R_{ct}$) of the working electrode, respectively (Fig. S11). To better understand the results, the EIS spectra were fitted to the corresponding equivalent circuit and the results clearly showed that the OCCN had a significantly lower $R_{ct}$, reflecting a lower photocarrier transfer resistance (Table S3).

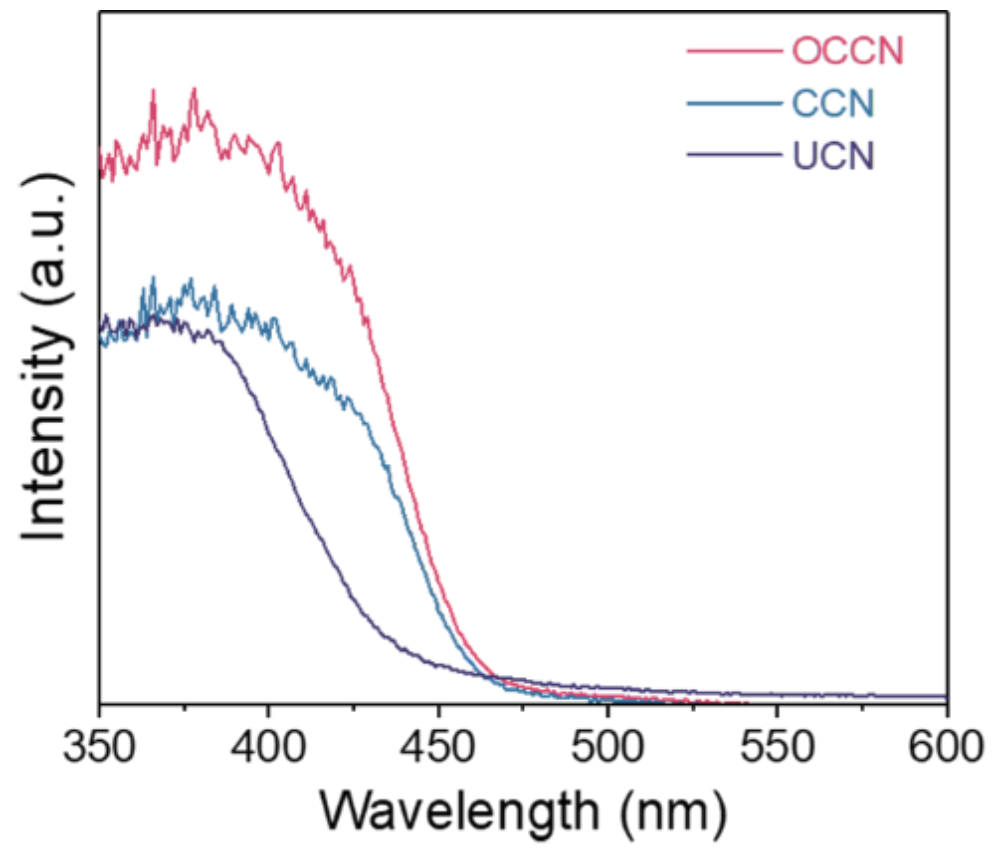


**Figure S12.** UV-Vis DRS of UCN, CCN and OCCN.

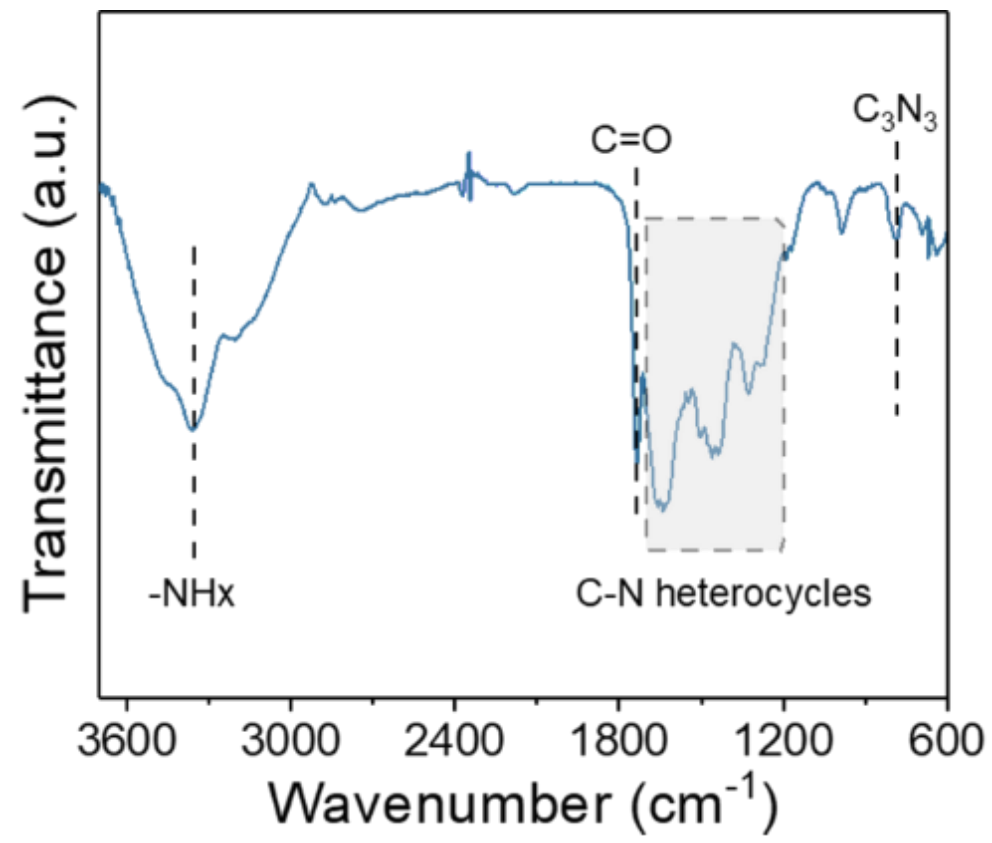


**Figure S13.** FT-IR spectra of U400.

FT-IR spectra of U400 exhibited characteristic stretching vibrations of $–NH_x$ (3000-3600 $cm^{-1}$) and carbonyl group (C=O, 1750 $cm^{-1}$), along with distinct stretching and bending vibrations associated with conjugated C–N heterocycles. Additionally, a strong band at 810 $cm^{-1}$ was observed, corresponding to the breathing mode of the triazine ring ($C_3N_3$)[6].

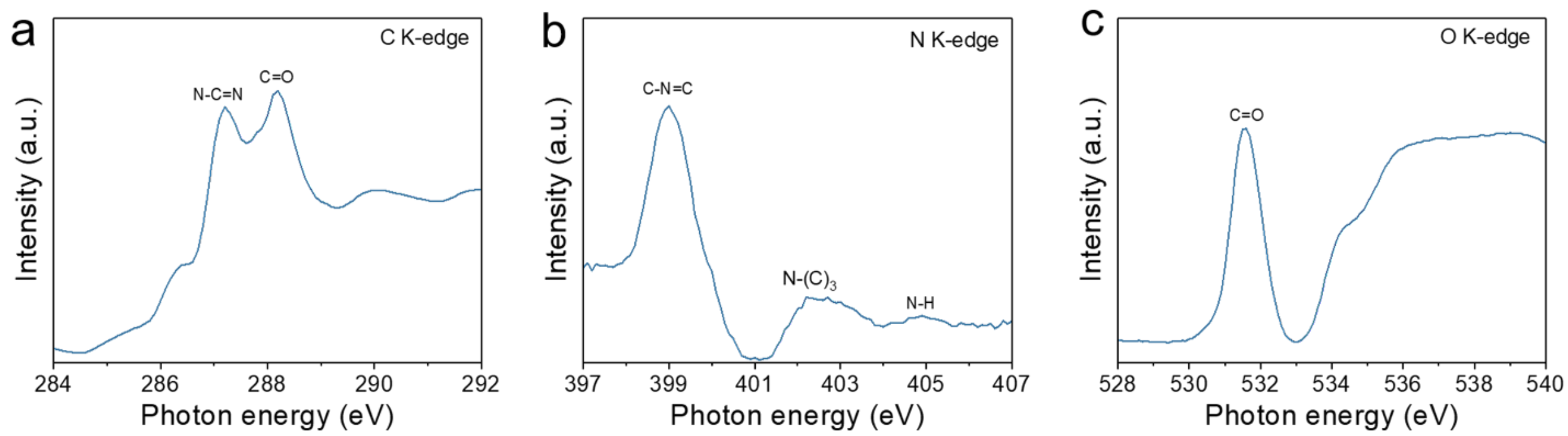


**Figure S14.** NEXAFS spectra including (a) C K-edge, (b) N K-edge, and (c) O K-edge of U400.

NEXAFS was conducted to investigate the local electronic and atomic structure of U400. In the C K-edge NEXAFS spectra (Fig. S14a), two distinct peaks at 287.2 and 288.2 eV were observed, corresponding to electron transitions from the C 1s to 2p $\pi^*$ orbitals, associated with N–C=N and C=O groups in $sp^2$ hybridized states[7,8]. N K-edge spectra (Fig. S14b) exhibited peaks at 399.0, 402.3, and 404.9 eV, which are attributed to C–N=C, N-$(C)_3$, and N-H bonding configurations, respectively. Additionally, a peak at 532.5 eV in the O K-edge spectra (Fig. S14c) was assigned to carbonyl (C=O) structures. These spectral features collectively reveal the chemical structure of U400, as schematically illustrated in Fig. S15[9].

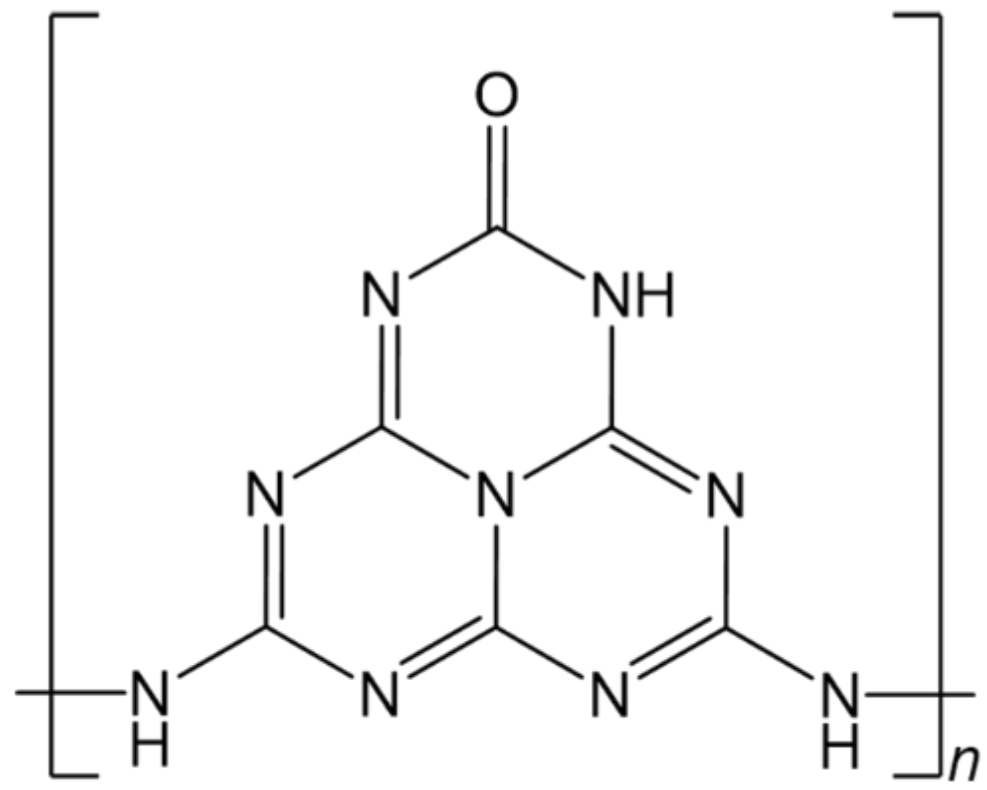

**Figure S15.** Chemical structure of U-400.

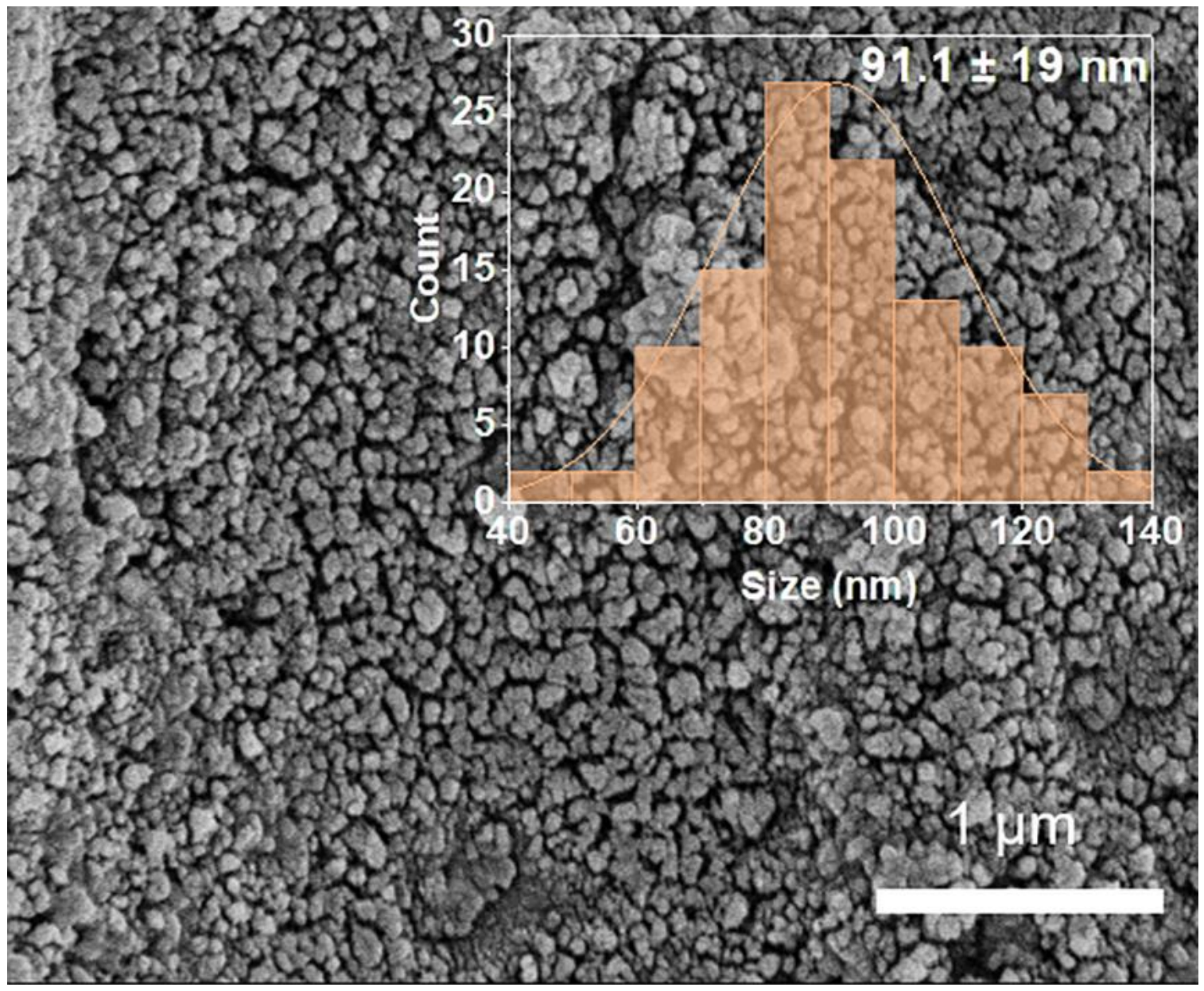


**Figure S16.** SEM image of OCCN, with an inset showing the particle size distribution histogram for OCCN.

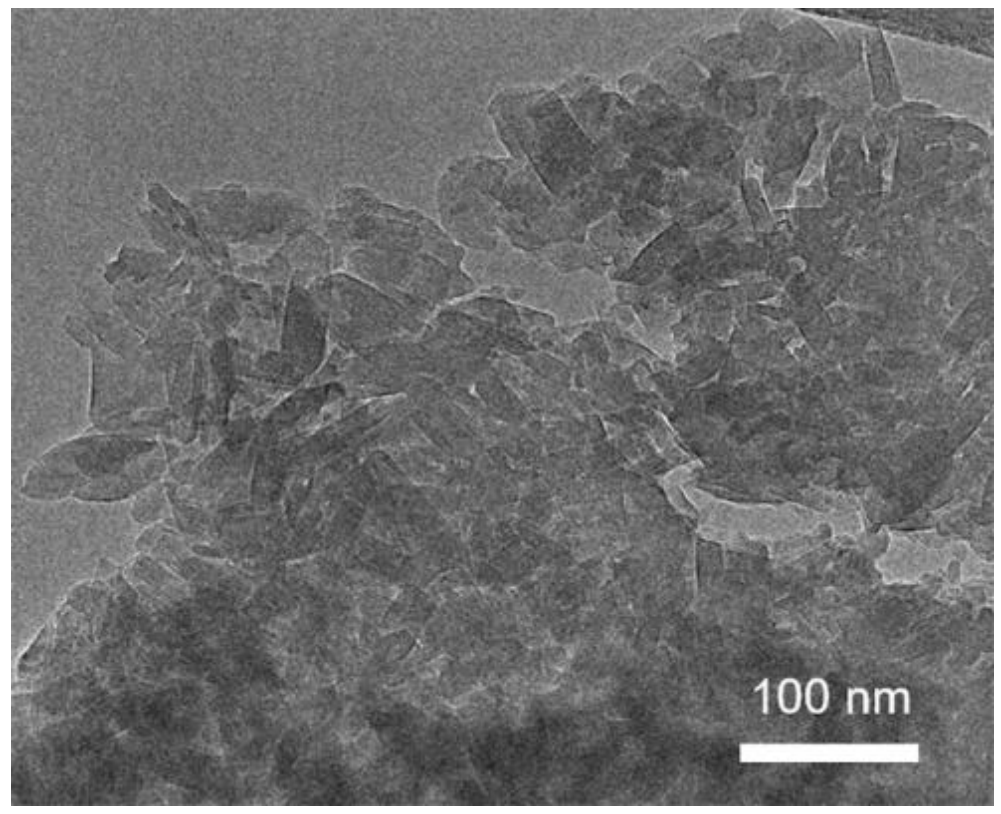


**Figure S17.** TEM image of OCCN.

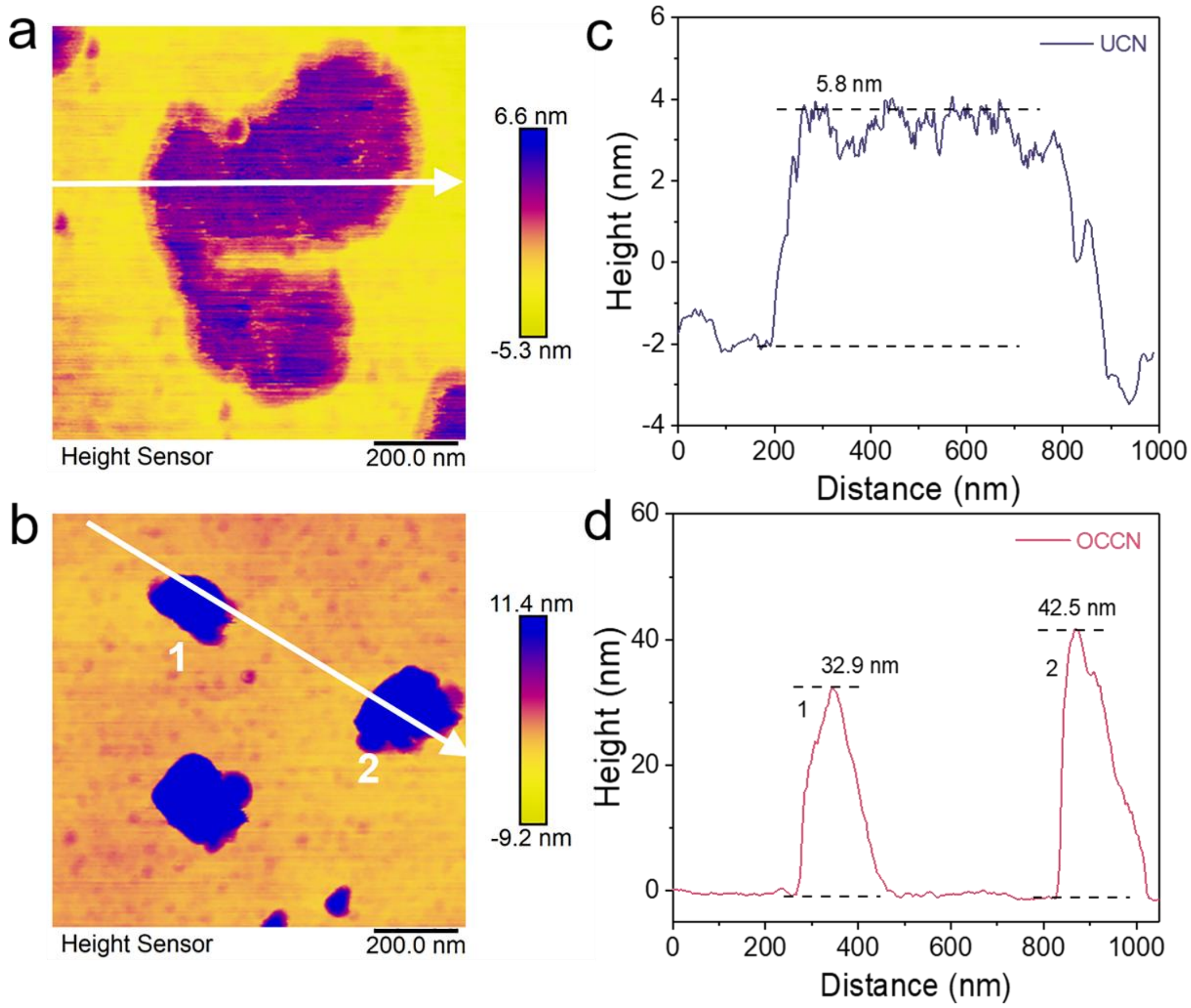


**Figure S18.** AFM images of UCN (a) and OCCN (b), along with their corresponding cross-sectional height profiles (c, d), extracted from the marked lines in the images.

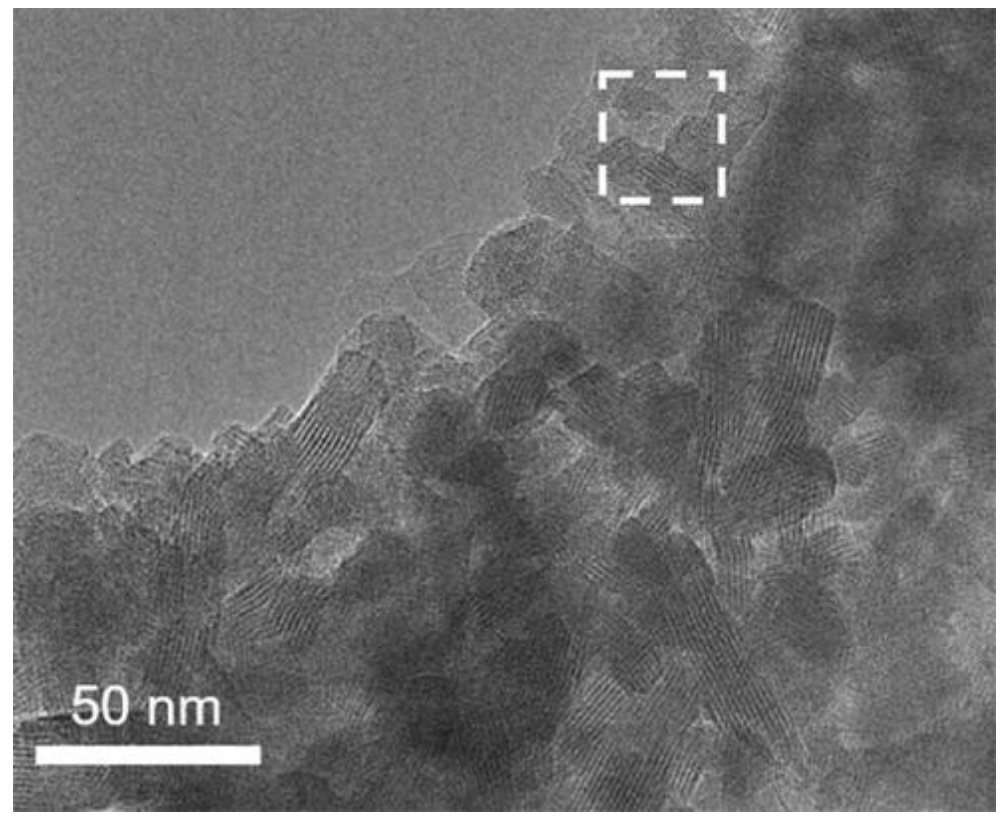


**Figure S19.** HRTEM image of OCCN.

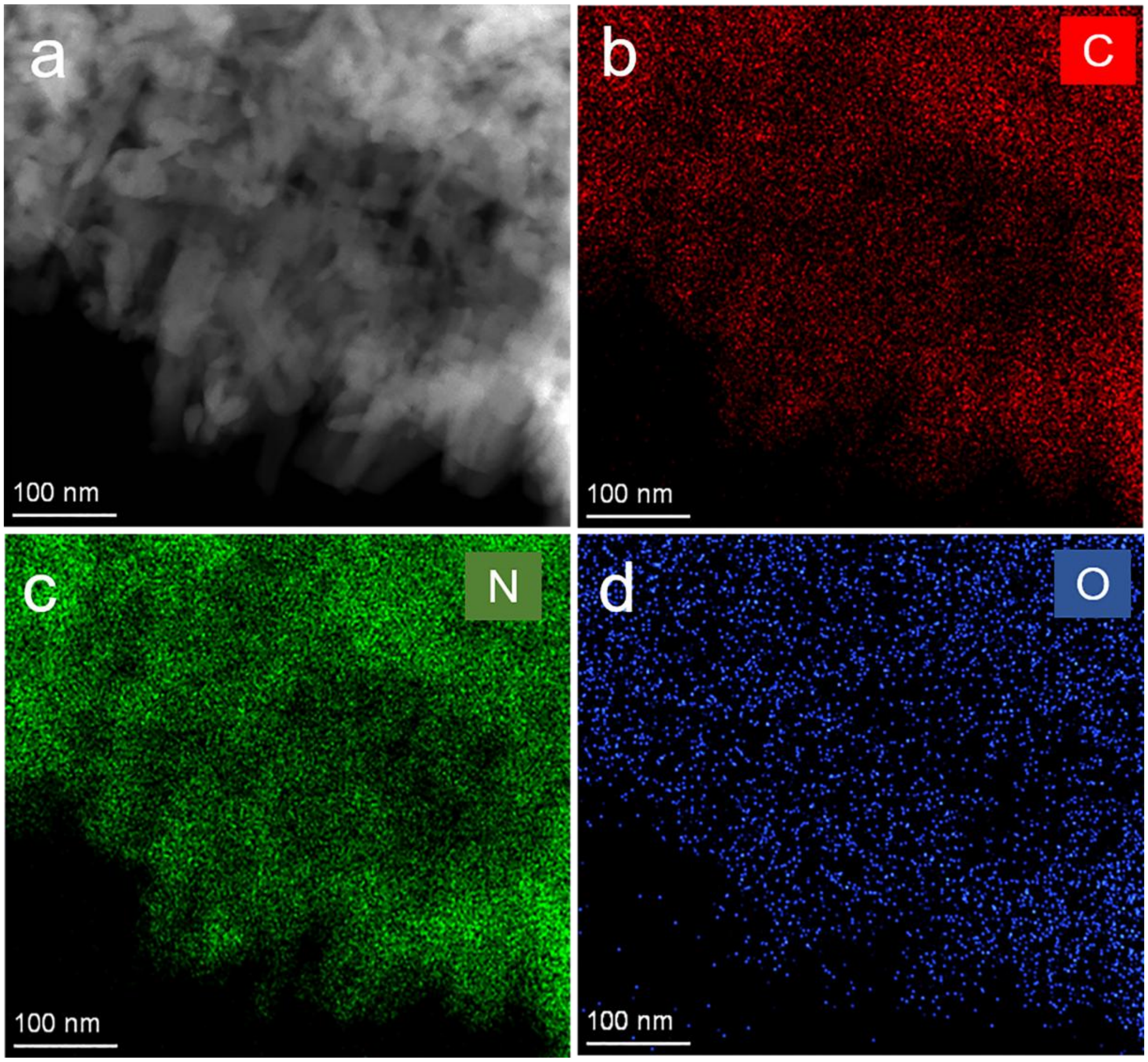


**Figure S20.** HAADF-STEM (a) and EDX mapping images of C (b), N (c), and O (d) for OCCN.

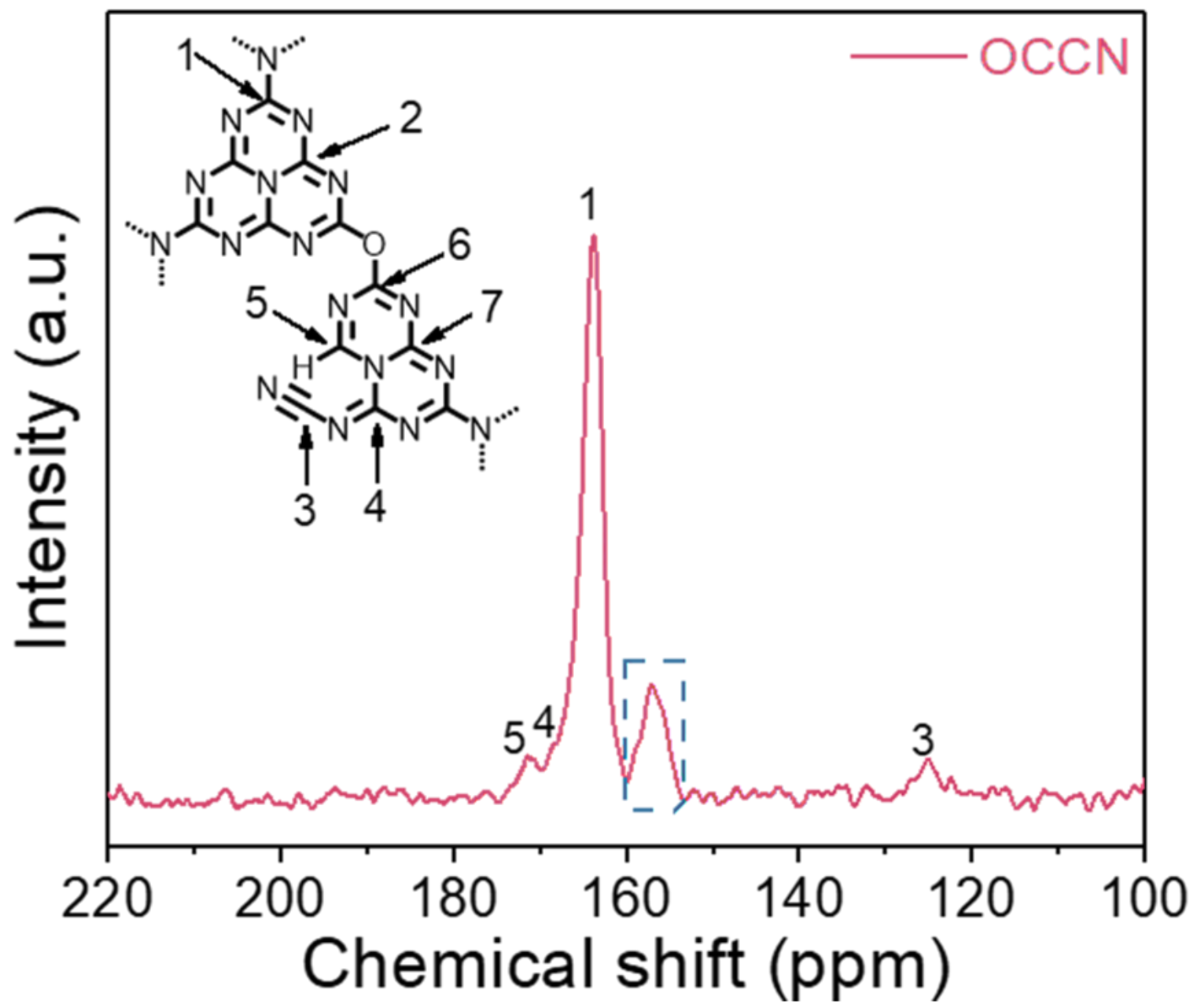


**Figure S21.** $^{13}C$ NMR spectra of OCCN.

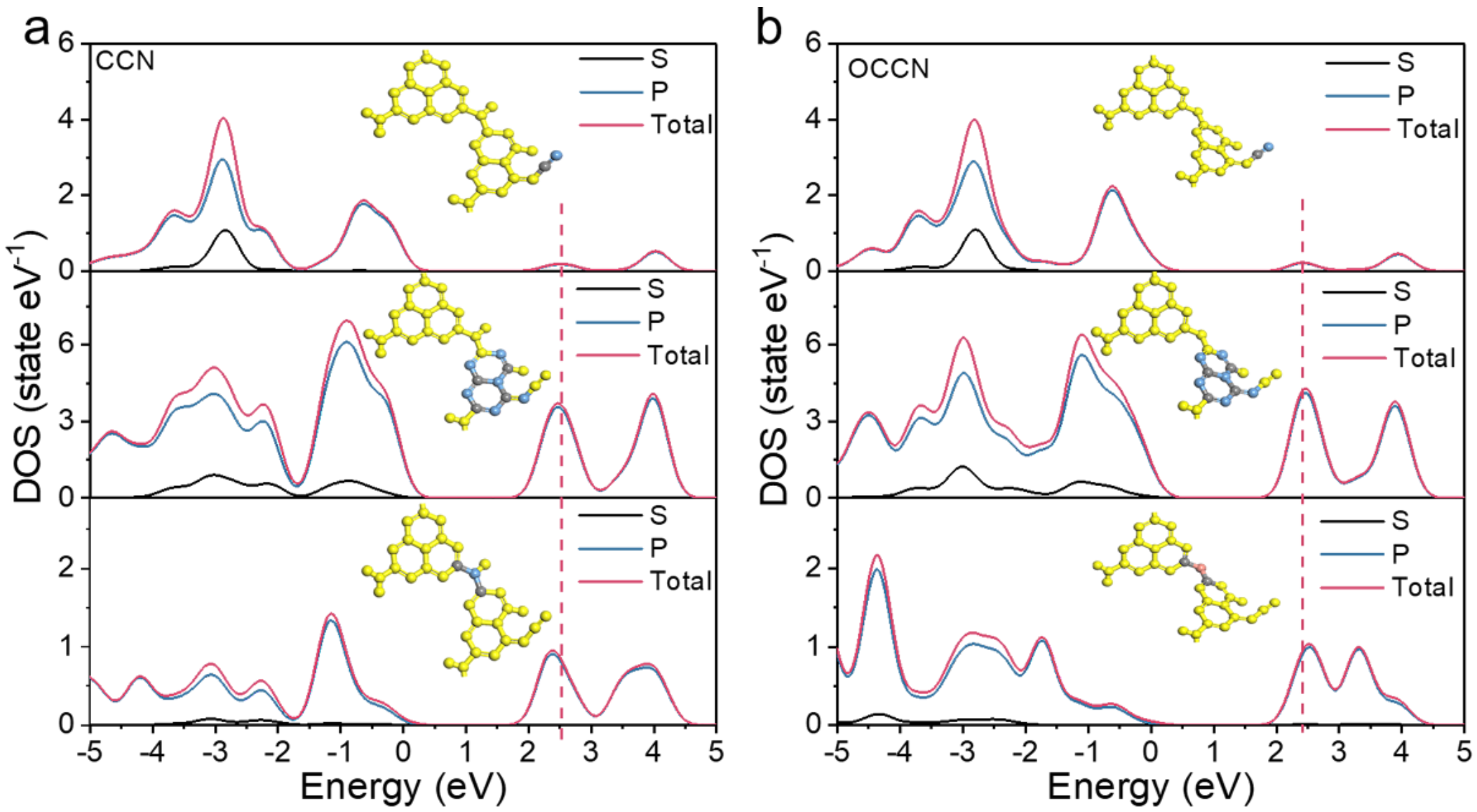


**Figure S22.** PDOS of (a) CCN and (b) OCCN; Inset: atomic structures for PDOS calculation.

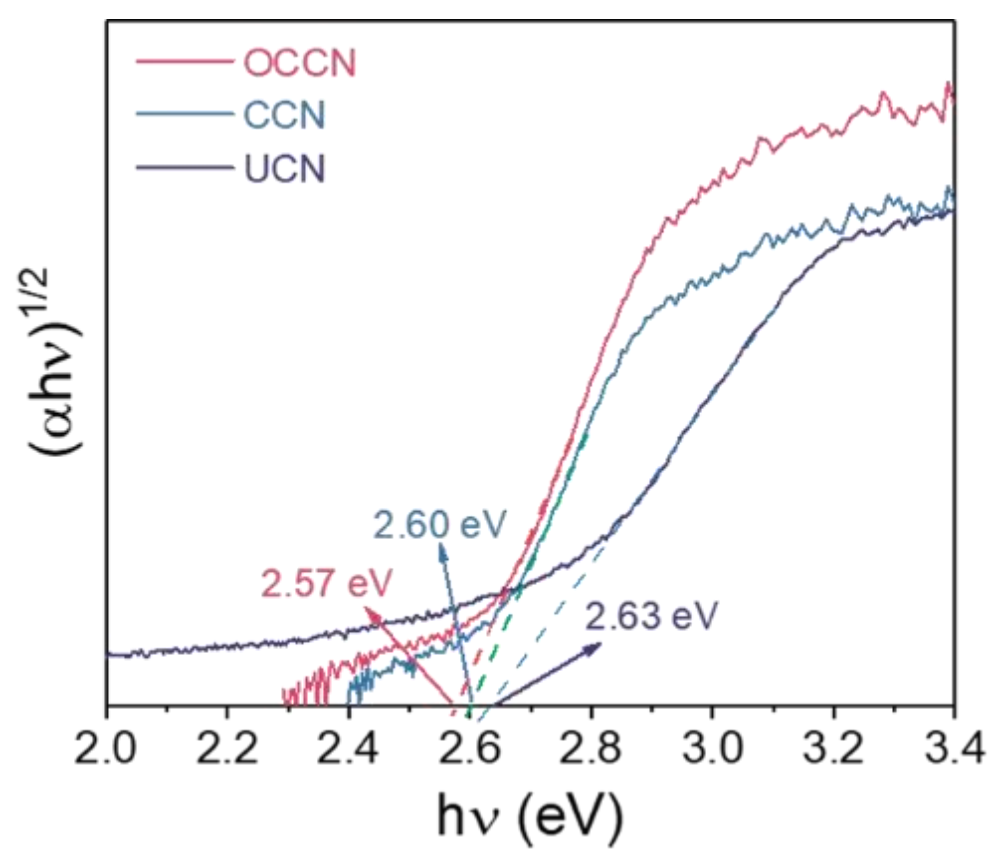


**Figure S23.** Tauc plots for estimating the bandgaps of prepared samples.

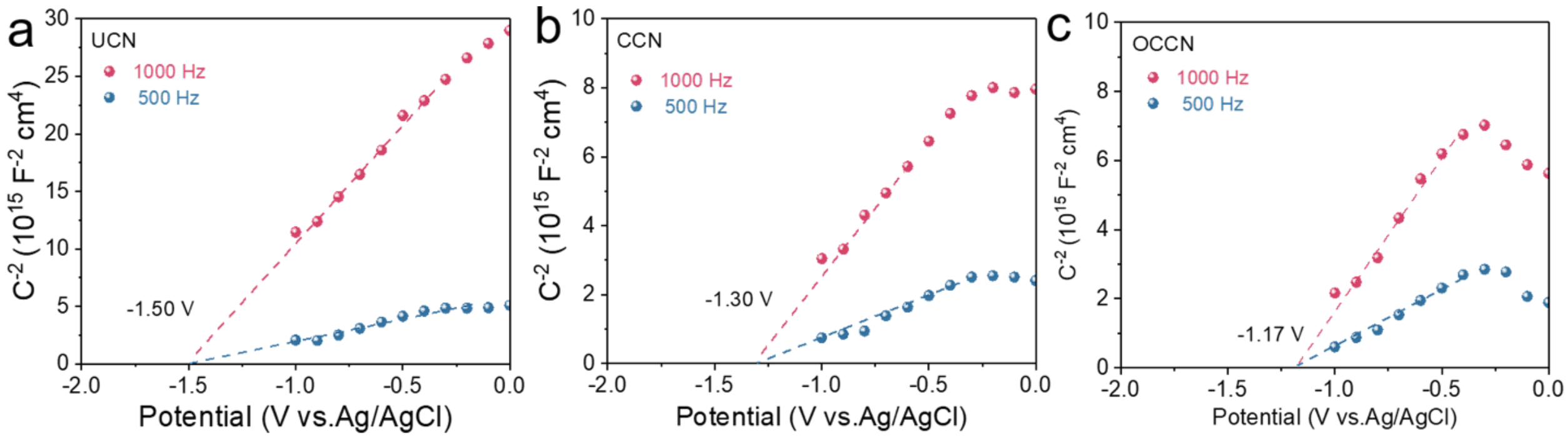


**Figure S24.** Mott-Schottky plots of (a) UCN, (b) CCN, and (c) OCCN.

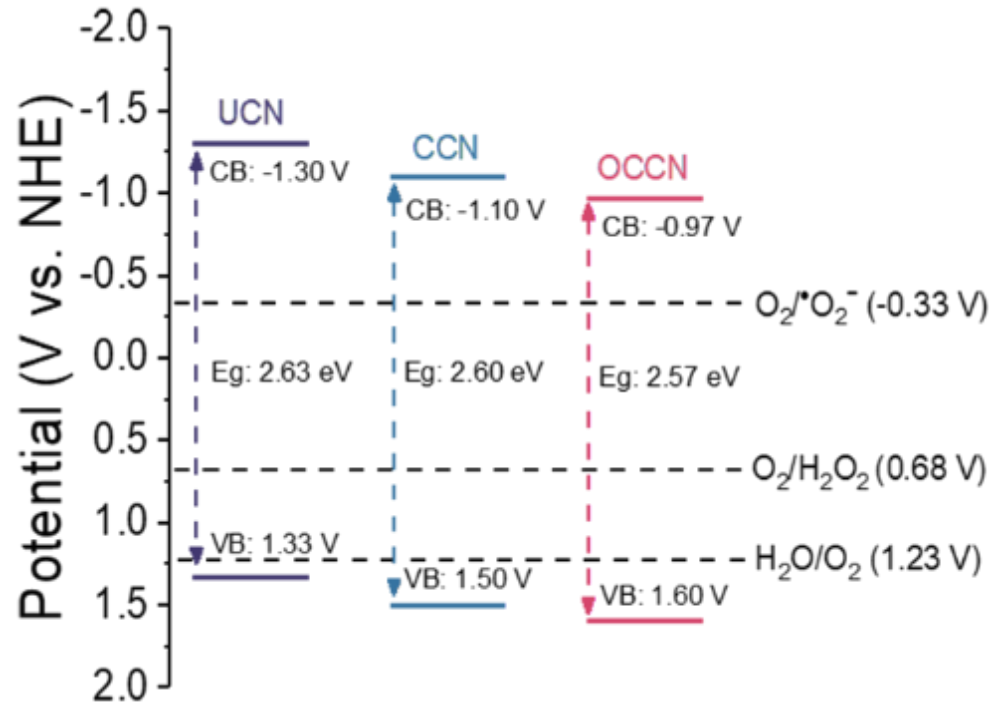


**Figure S25.** Band structures of UCN, CCN, and OCCN.

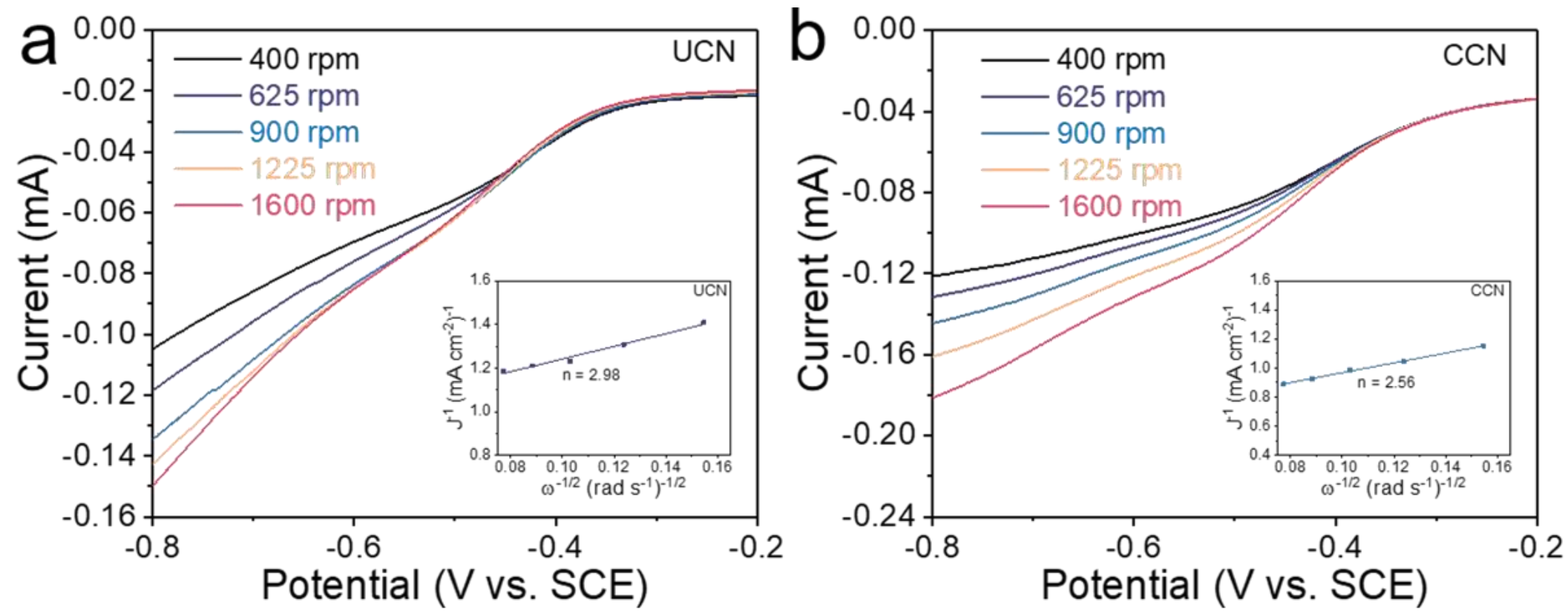


**Figure S26.** LSV curves of (a) UCN and (b) CCN measured using RDE. The insets show the fitted K–L plot.

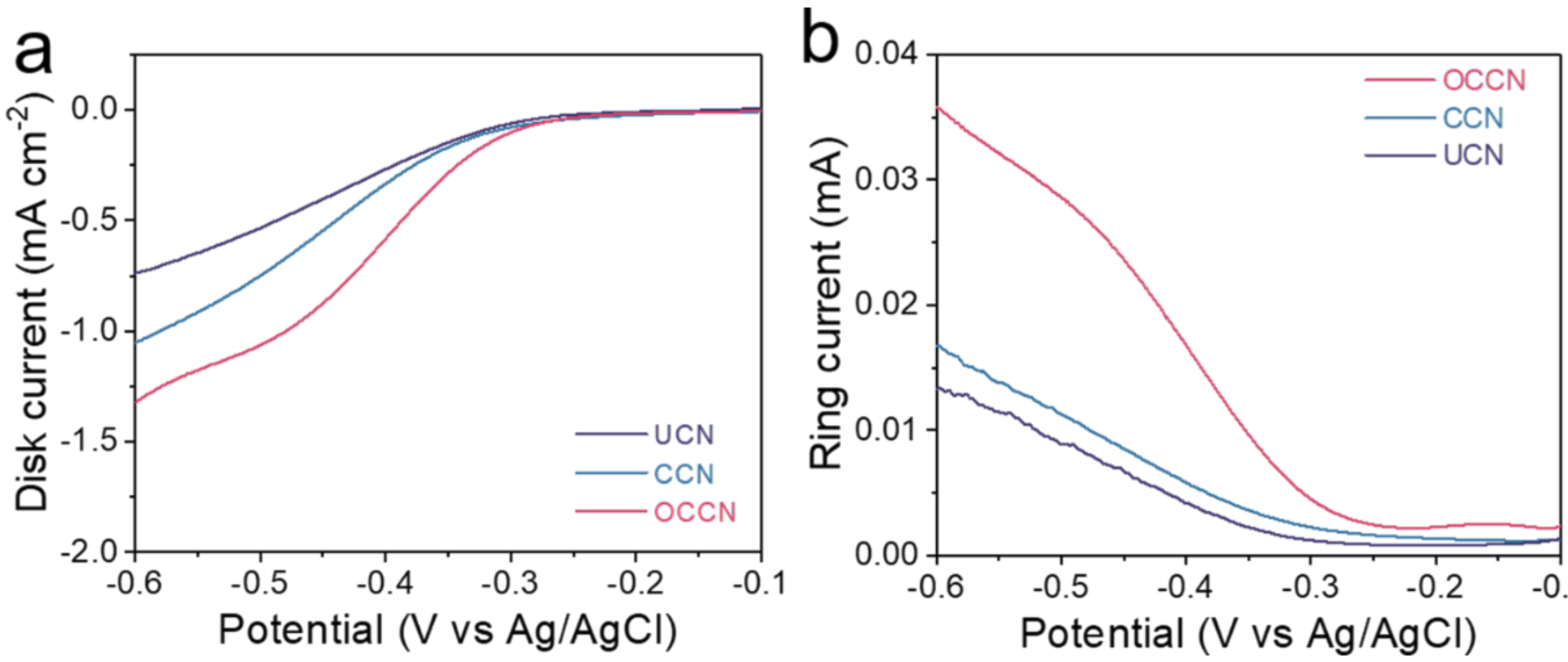


**Figure S27.** RRDE curves of (a) disk current and (b) ring current on UCN, CCN, and OCCN.

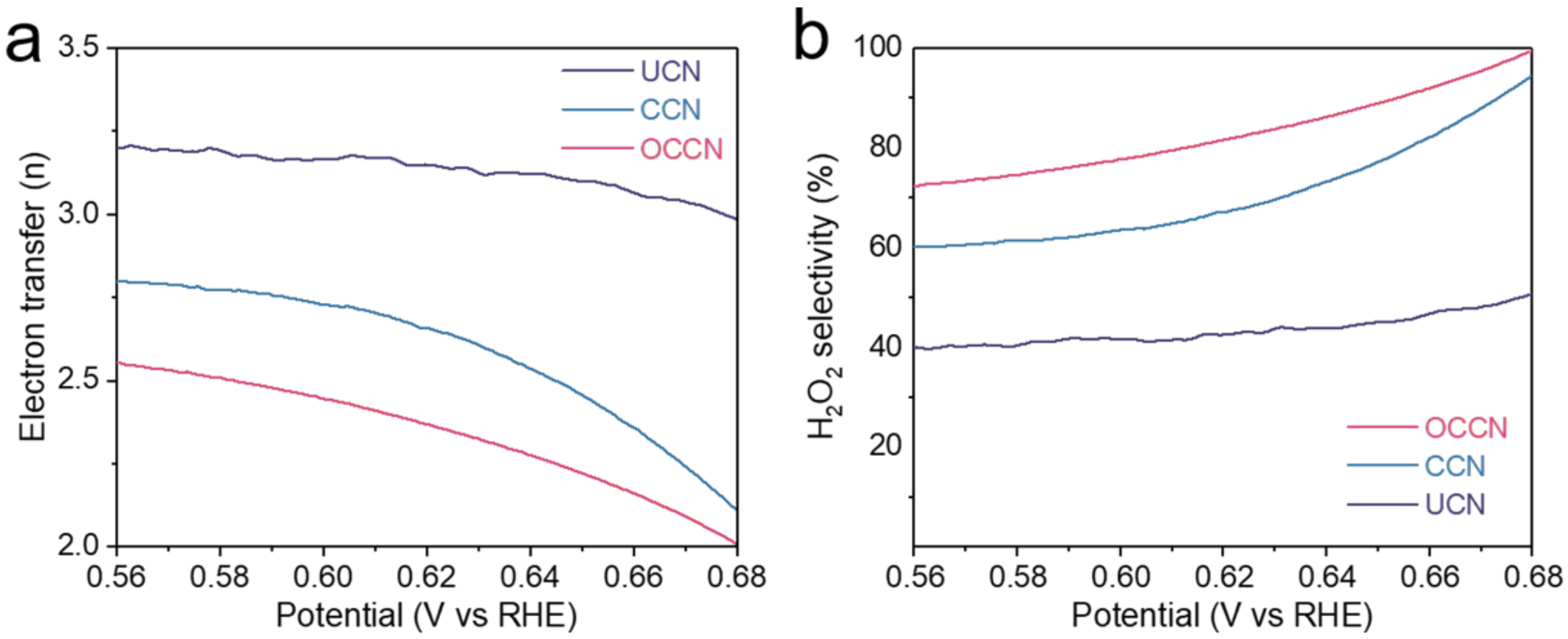


**Figure S28.** (a) Electron transfer numbers and (b) $H_2O_2$ selectivity at different potentials for UCN, CCN, and OCCN.

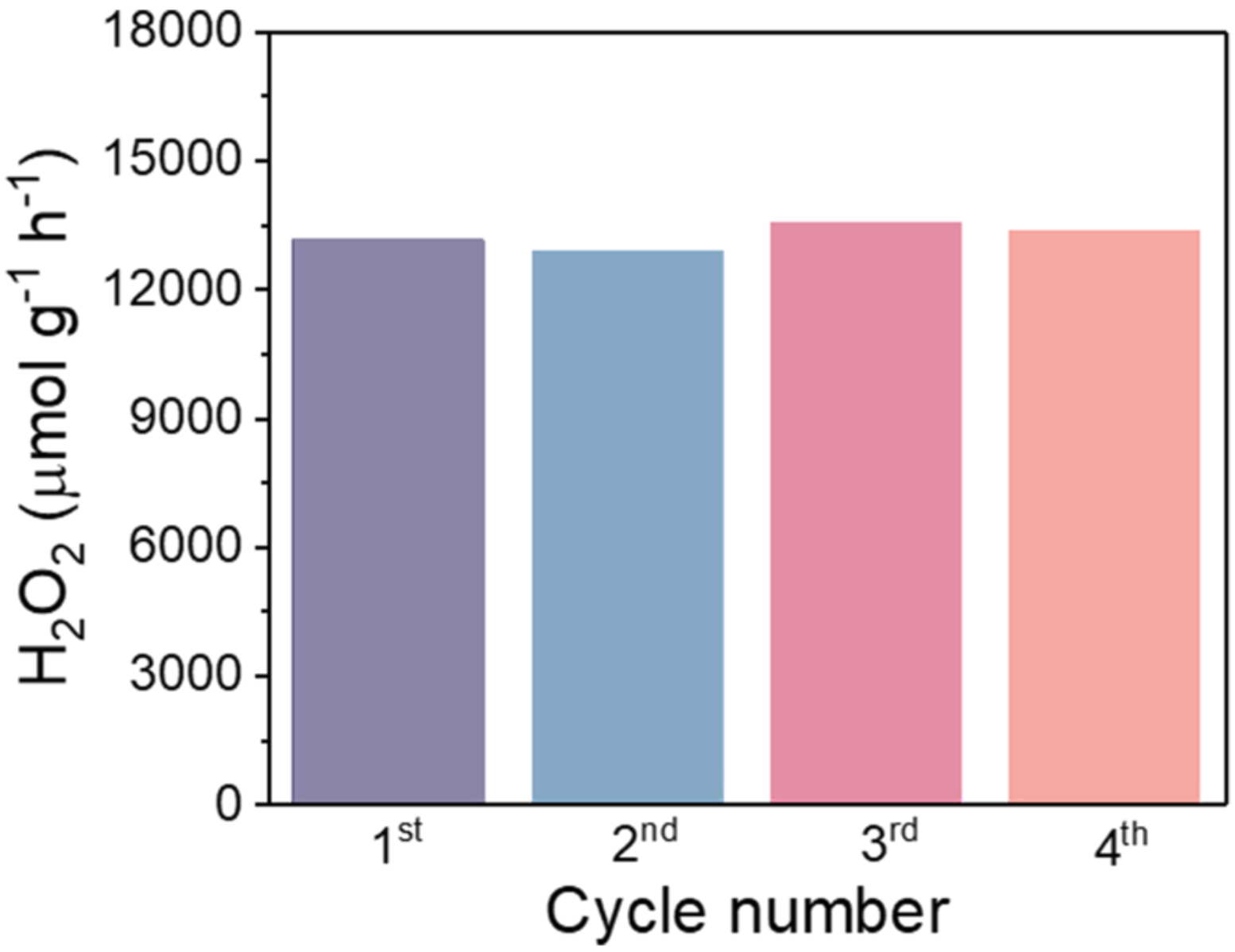


**Figure S29.** Stability tests of photocatalytic $H_2O_2$ generation using OCCN.

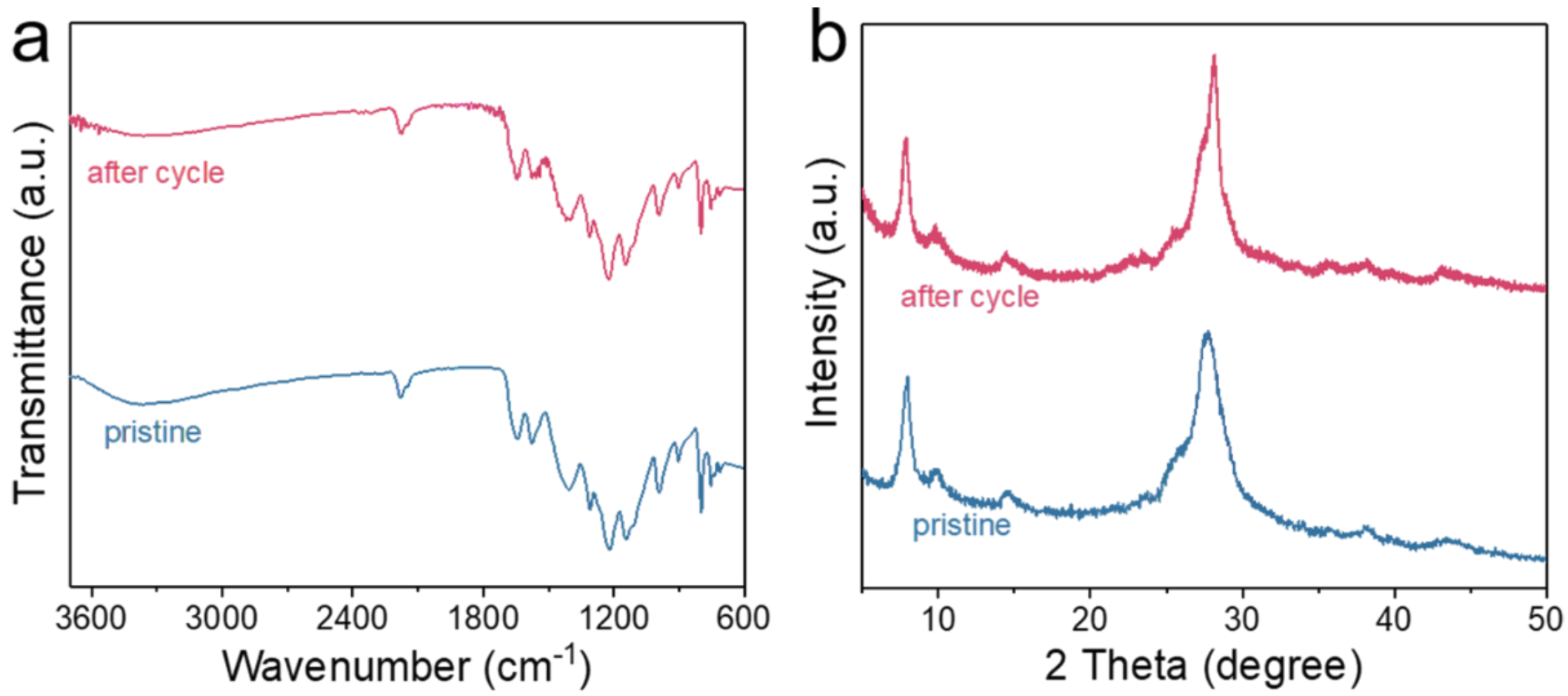


**Figure S30.** (a) FT-IR spectra and (b) PXRD patterns of OCCN before and after the stability evaluation.

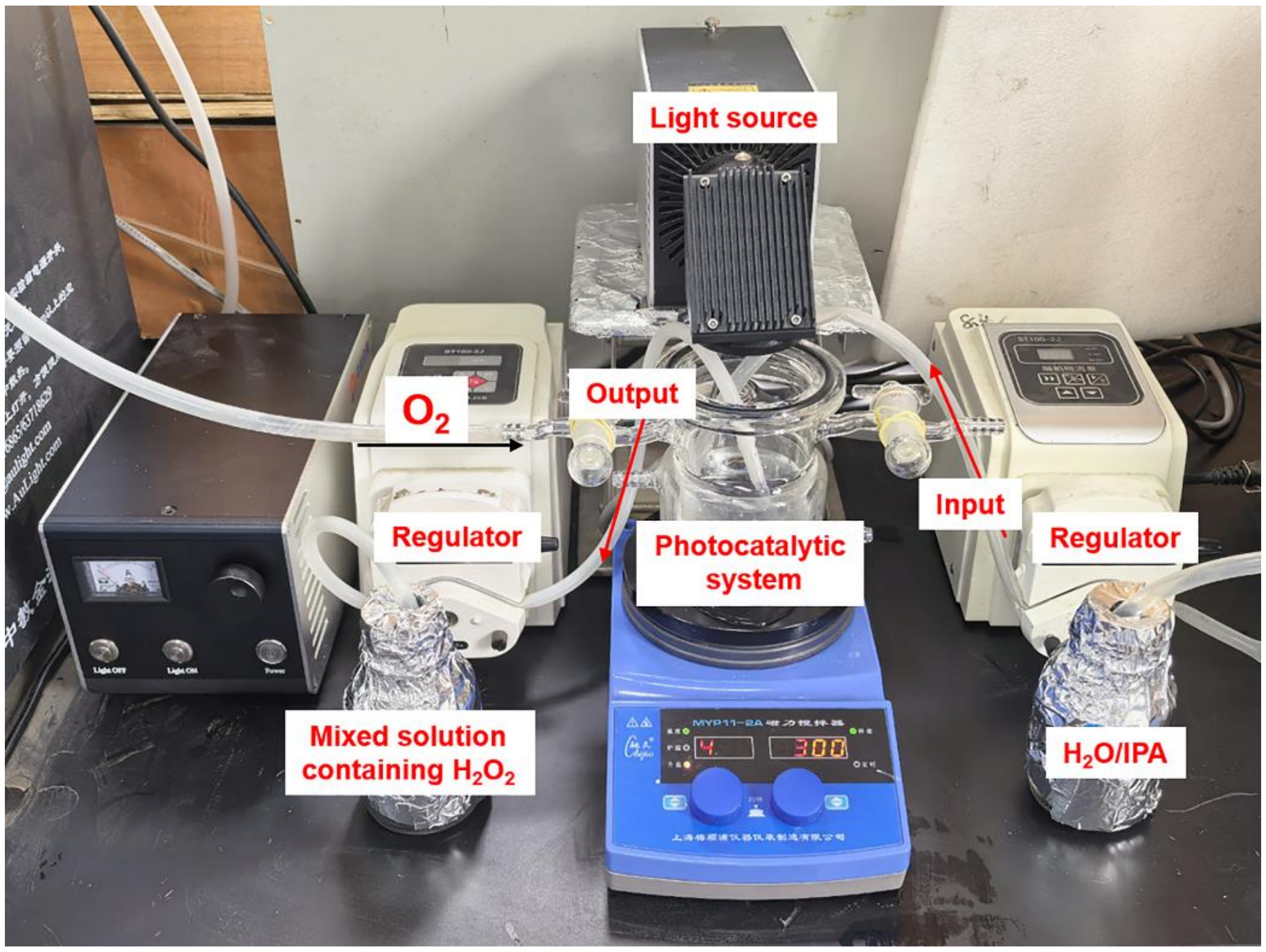


**Figure S31.** Photo for continuous photocatalytic $H_2O_2$ production system.

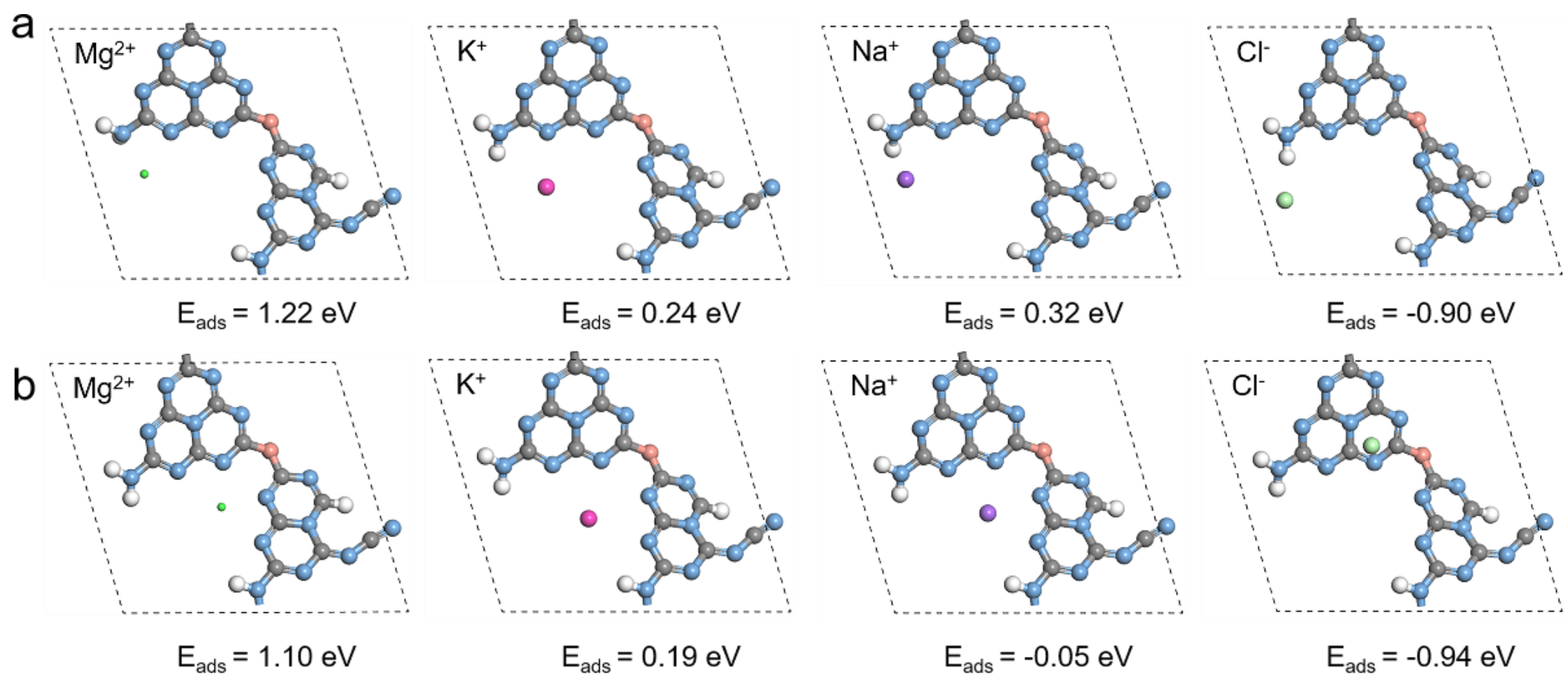


**Figure S32.** Adsorption configurations of $Na^+$, $K^+$, $Mg^{2+}$, and $Cl^-$ ions at cyano group sites (a) and ether bond sites (b) of OCCN.

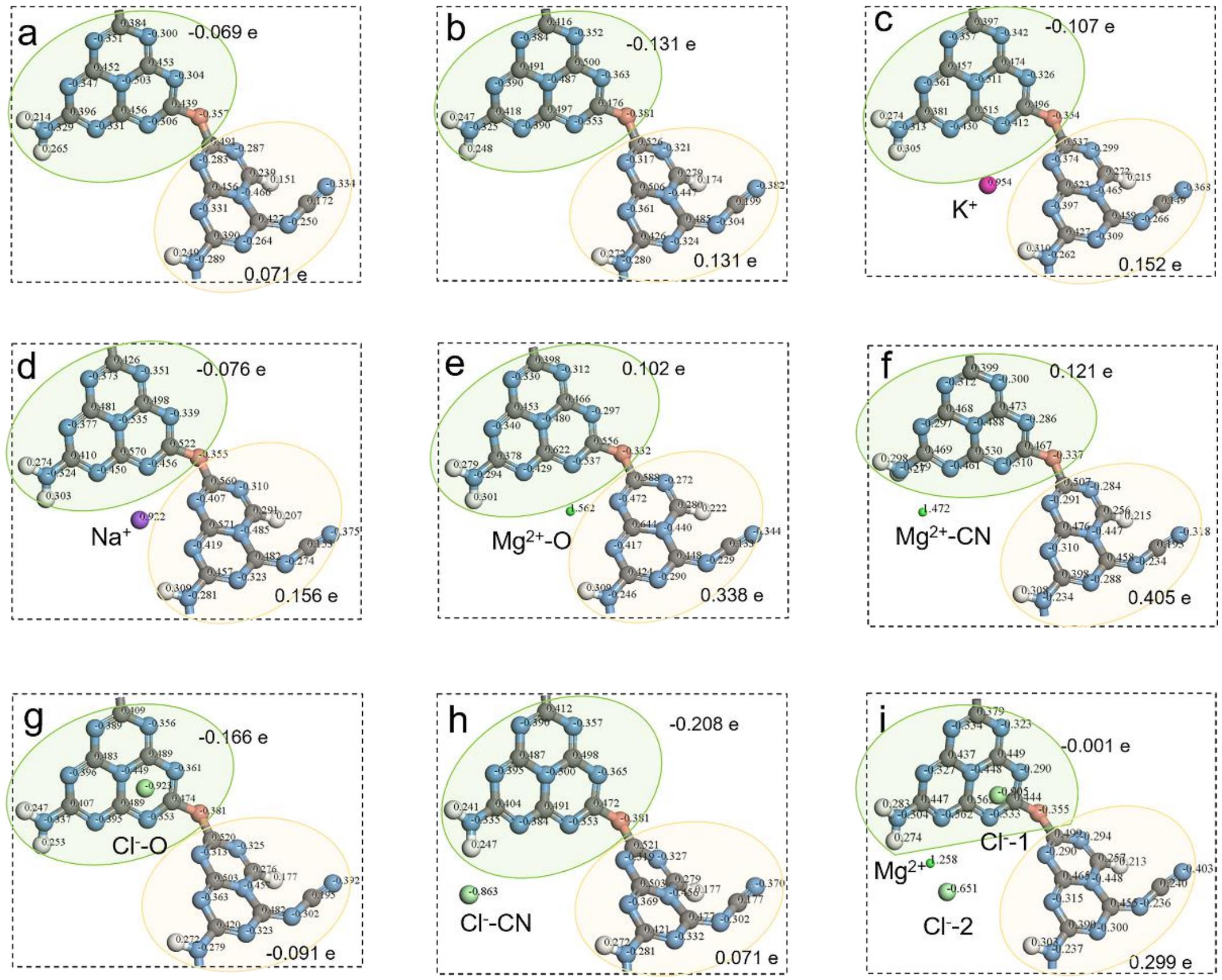


**Figure S33.** Mulliken charge distribution of OCCN: (a) pristine OCCN; (b) OCCN in water solvent (dielectric constant = 78.54); (c–i) OCCN in water solvent with the addition of (c) $K^+$, (d) $Na^+$, (e,f) $Mg^{2+}$, (g,h) $Cl^-$, and (i) $Mg^{2+}$ and $Cl^-$ co-adsorbed, respectively.

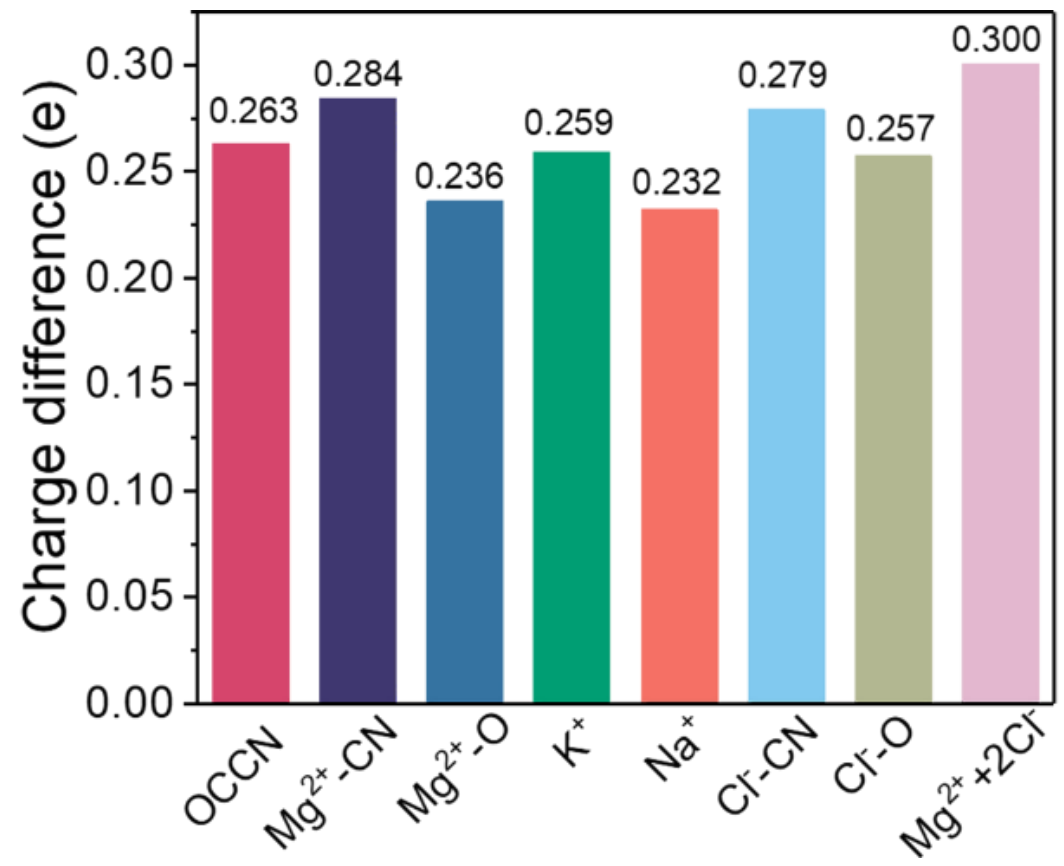


**Figure S34.** Mulliken charge distribution difference of OCCN: in water solvent, and that with the addition of $K^+$, $Na^+$, $Mg^{2+}$, $Cl^-$, and $Mg^{2+}/Cl^-$ co-adsorption, respectively.

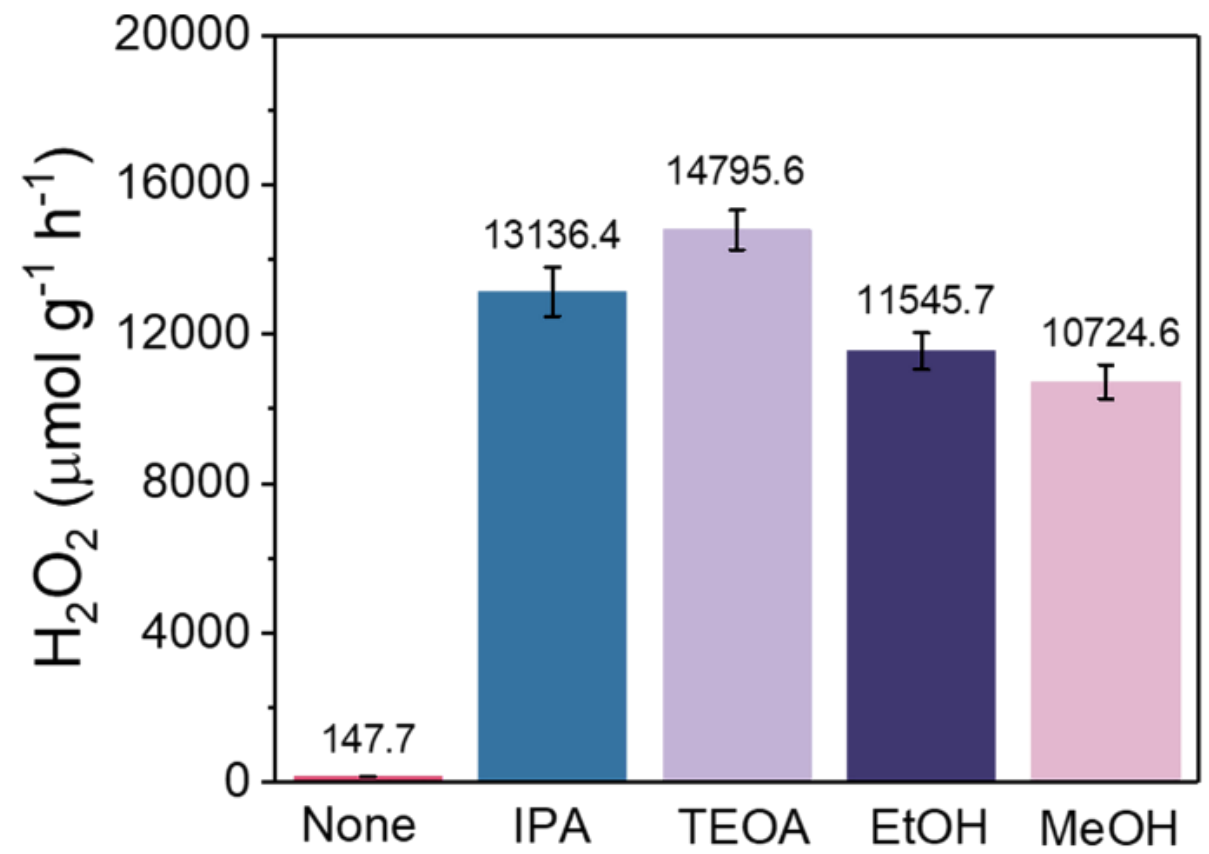


**Figure S35.** Influence of different hole scavengers on the $H_2O_2$ production over OCCN.

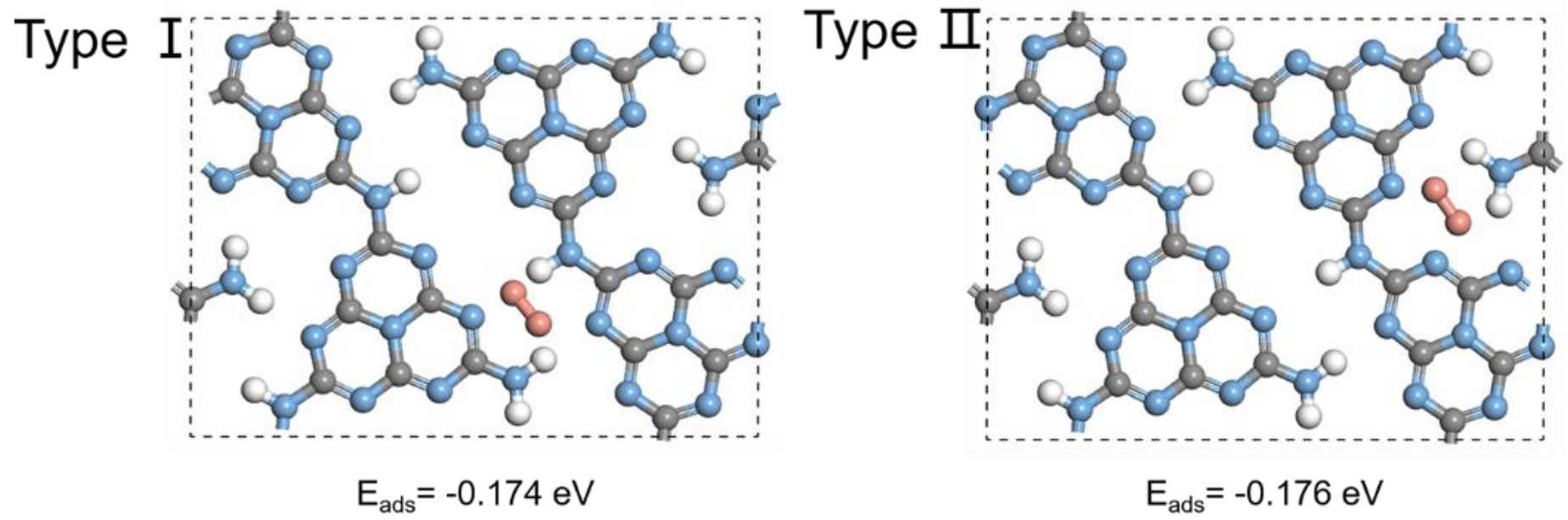


**Figure S36.** Different $O_2$ adsorption configurations and adsorption energy for UCN.

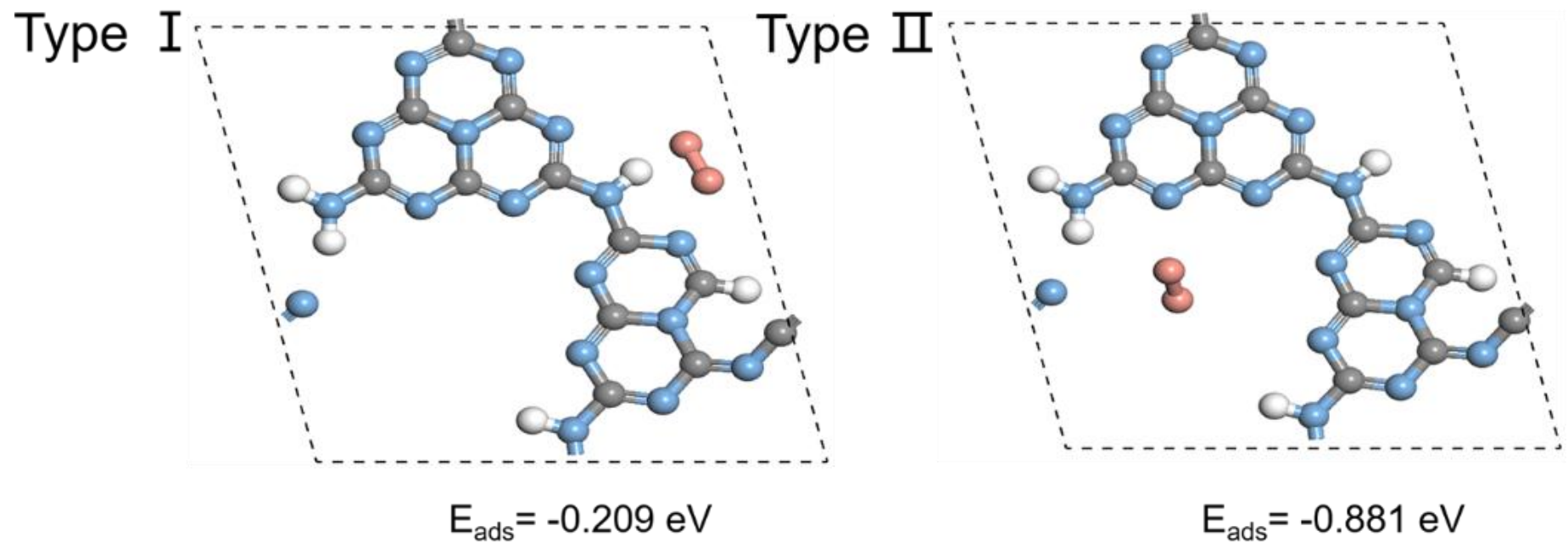


**Figure S37.** Different $O_2$ adsorption configurations and adsorption energy for CCN.

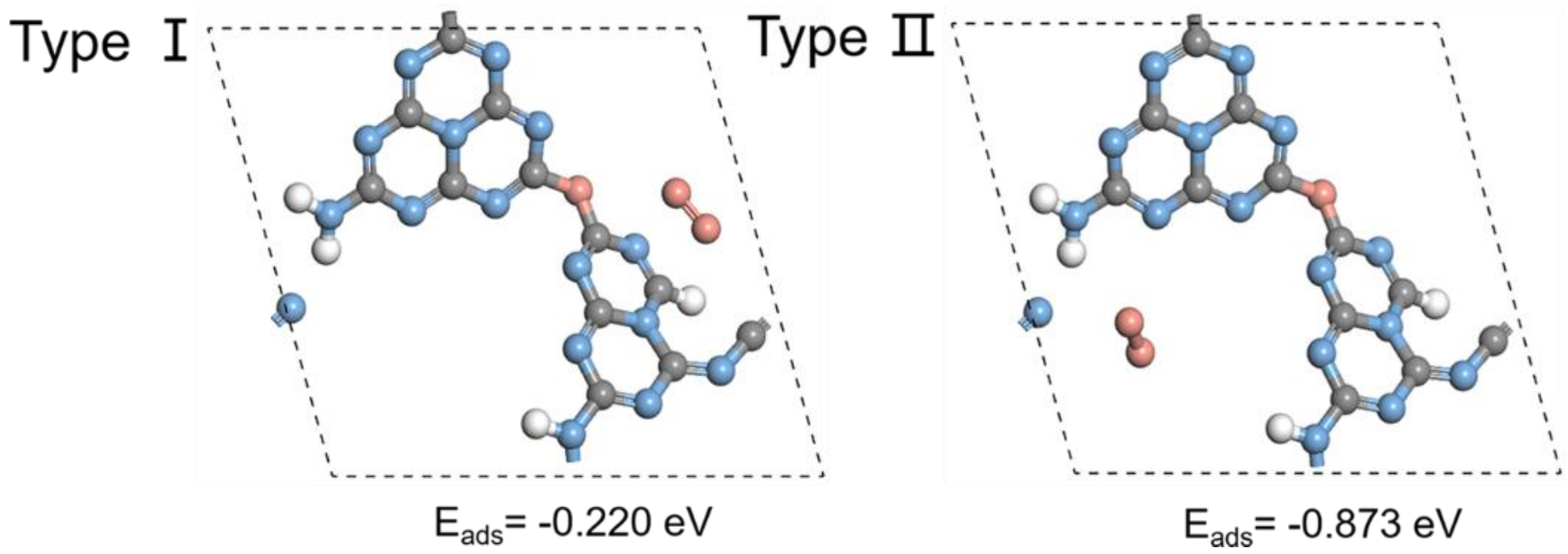


**Figure S38.** Different $O_2$ adsorption configurations and adsorption energy for OCCN.

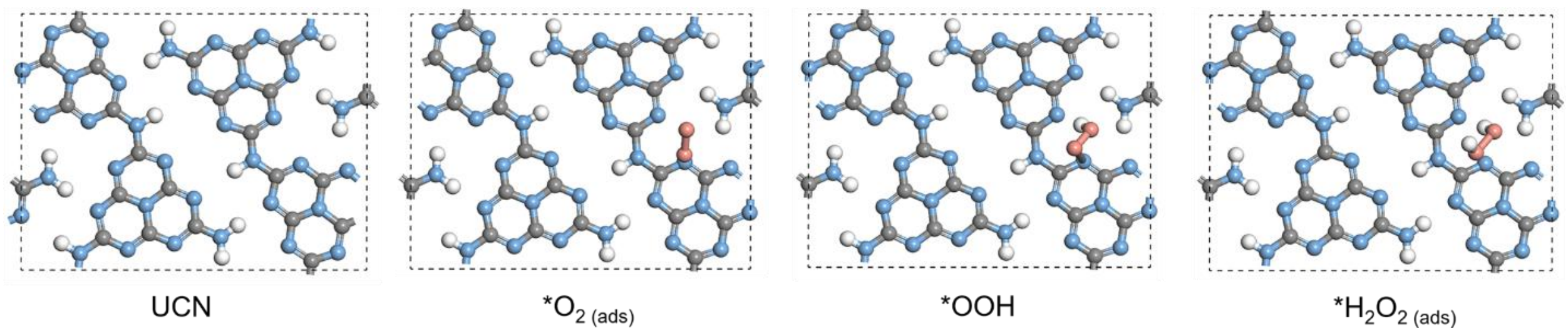


**Figure S39.** Optimized modeling for reduction of $O_2$ into $H_2O_2$ via an indirect two-step single-electron ORR process on UCN.

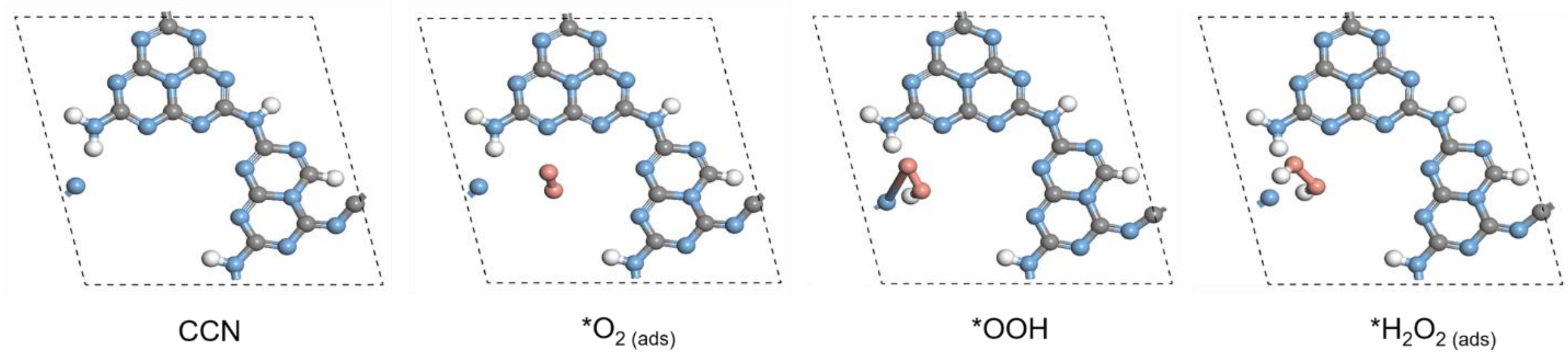


**Figure S40.** Optimized modeling for reduction of $O_2$ into $H_2O_2$ via an indirect two-step single-electron ORR process on CCN.

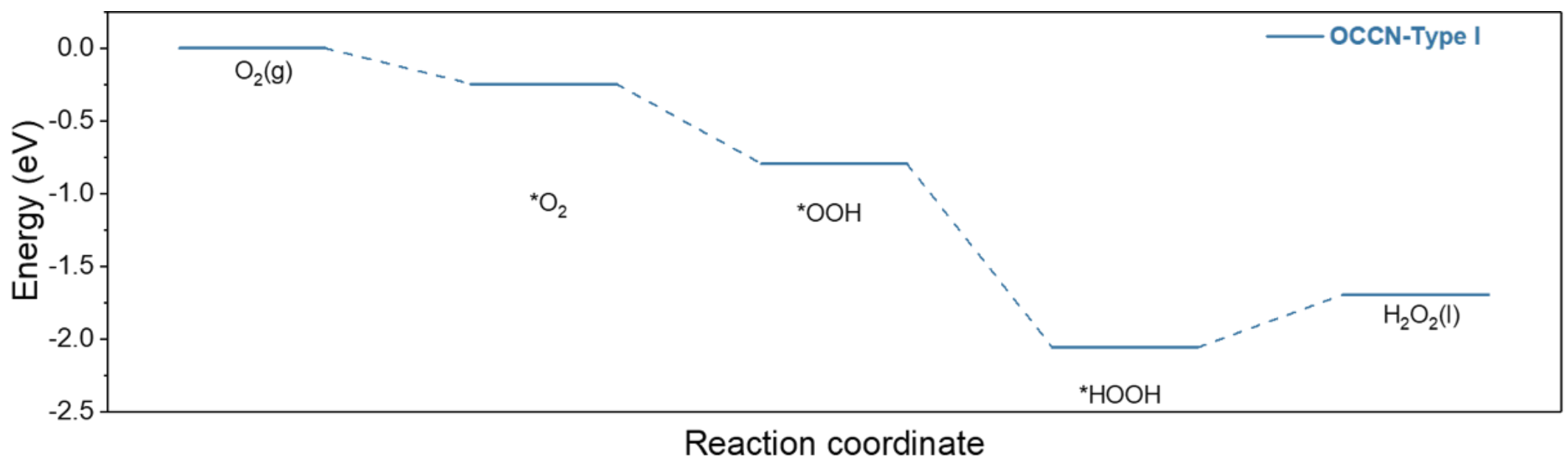


**Figure S41.** Free energy diagrams for $O_2$ to $H_2O_2$ at the ether bond site of OCCN through indirect two-electron ORR pathway.

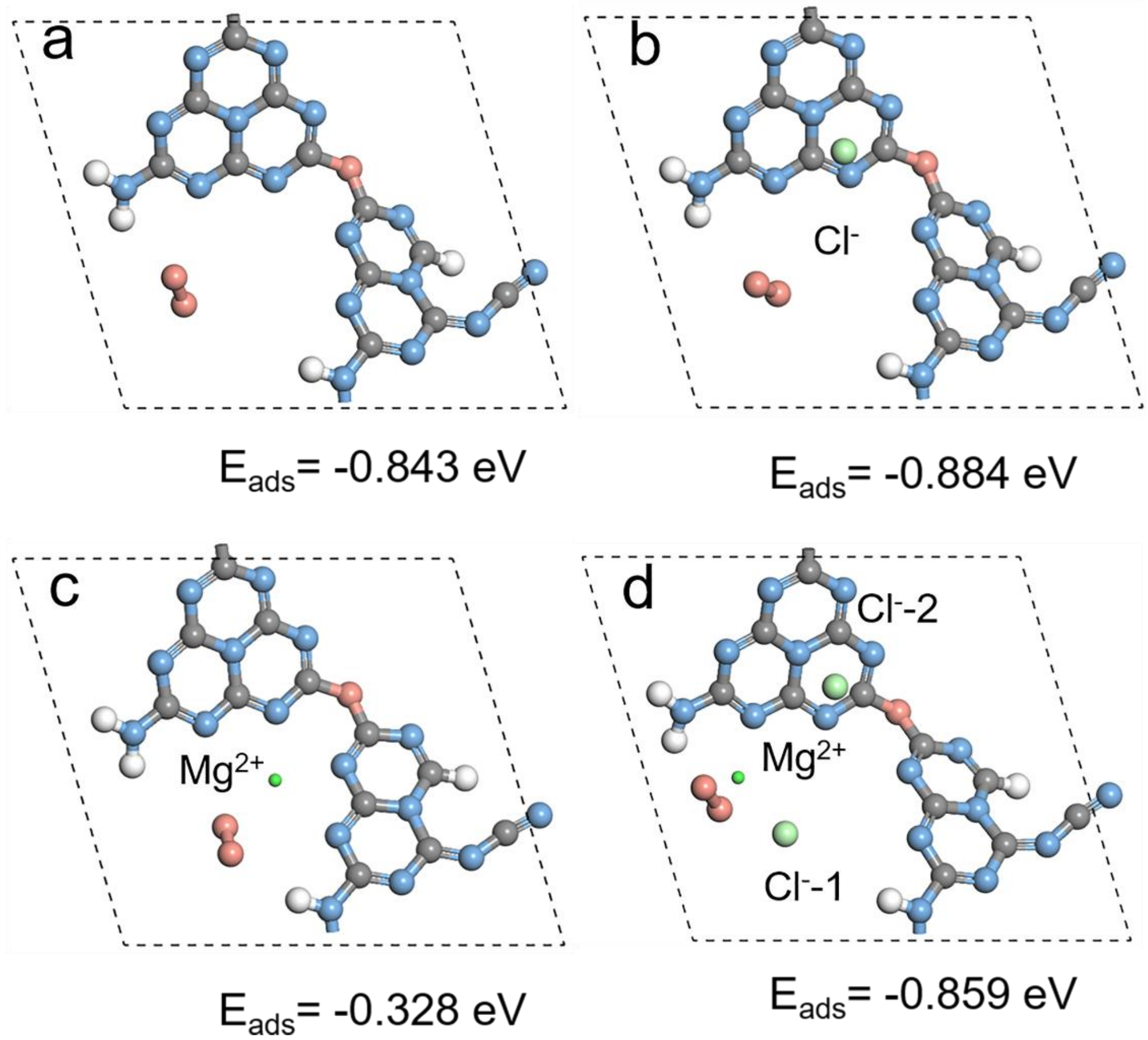


**Figure S42.** Under solvation conditions: (a) adsorption mode and adsorption energy of $O_2$ at cyano site of OCCN; (b) in the presence of $Cl^-$, (c) in the presence of $Mg^{2+}$, and (d) in the presence of co-existing $Cl^-/Mg^{2+}$ for $O_2$ adsorption on OCCN.

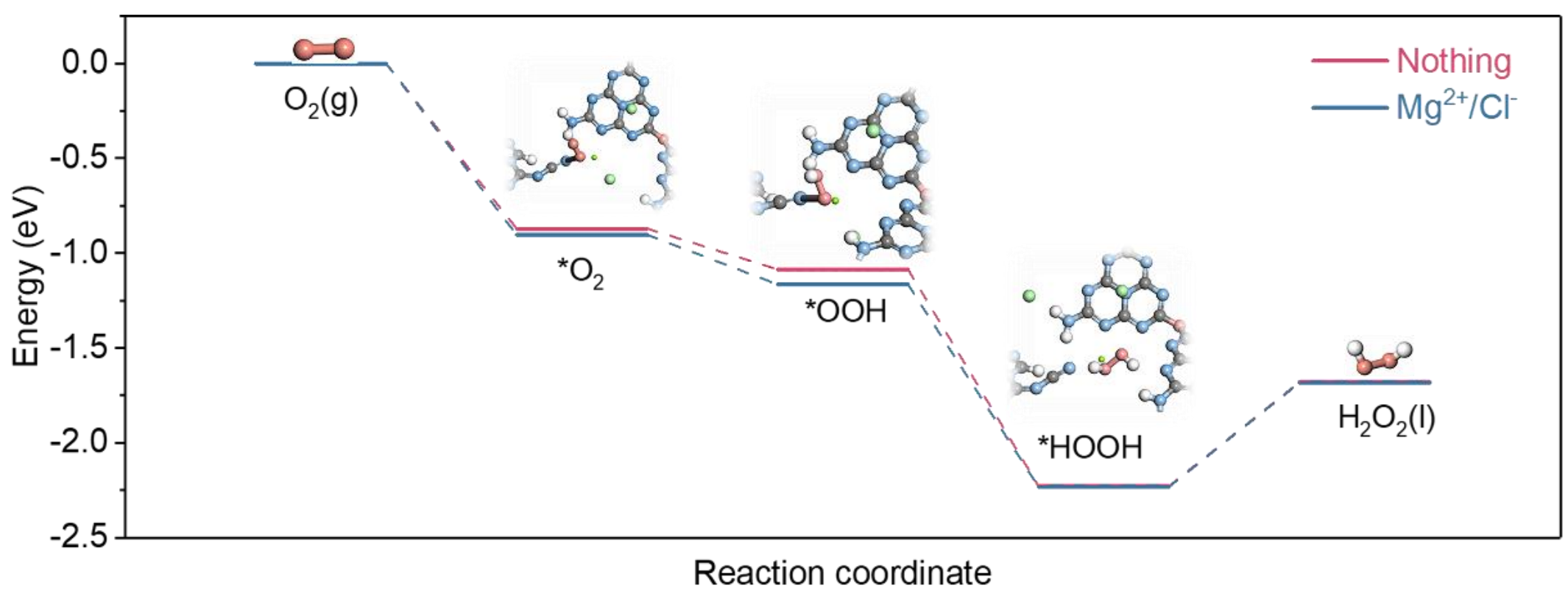


**Figure S43.** Free energy diagrams for $O_2$ to $H_2O_2$ via the indirect two-electron ORR pathway at cyano group site of OCCN in the presence of solvation and $Mg^{2+}/Cl^{-}$ ions.

**Table S1.** Surface atomic ratios of all elements determined by XPS.

| Sample | %C (at) | %N (at) | %O (at) | %Na (at) | %K (at) | %Cl (at) |
|---|---|---|---|---|---|---|
| CCN | 29.87 | 58.85 | 4.87 | 3.86 | 2.65 | / |
| OCCN | 37.64 | 46.15 | 12.08 | 2.35 | 1.88 | / |

**Table S2.** Mulliken charge distribution of UCN, CCN, and OCCN.

| UCN | Charge (e) | CCN | Charge (e) | OCCN | Charge (e) |
|---|---|---|---|---|---|
| H0 | 0.279 | H1 | 0.252 | H1 | 0.249 |
| H1 | 0.270 | H2 | 0.146 | H2 | 0.214 |
| H2 | 0.290 | H3 | 0.212 | H3 | 0.265 |
| H3 | 0.278 | H4 | 0.262 | H4 | 0.151 |
| H4 | 0.272 | H5 | 0.249 | C1 | 0.390 |
| H5 | 0.291 | C1 | 0.388 | C2 | 0.427 |
| C1 | 0.428 | C2 | 0.433 | C3 | 0.172 |
| C2 | 0.475 | C3 | 0.149 | C4 | 0.456 |
| C3 | 0.425 | C4 | 0.449 | C5 | 0.239 |
| C4 | 0.458 | C5 | 0.239 | C6 | 0.491 |
| C5 | 0.490 | C6 | 0.399 | C7 | 0.396 |
| C6 | 0.386 | C7 | 0.393 | C8 | 0.456 |
| C7 | 0.422 | C8 | 0.444 | C9 | 0.439 |
| C8 | 0.473 | C9 | 0.378 | C10 | 0.452 |
| C9 | 0.488 | C10 | 0.451 | C11 | 0.453 |
| C10 | 0.460 | C11 | 0.456 | C12 | 0.384 |
| C11 | 0.386 | C12 | 0.382 | N1 | -0.289 |
| C12 | 0.429 | N1 | -0.284 | N2 | -0.264 |
| N1 | -0.363 | N2 | -0.333 | N3 | -0.250 |
| N2 | -0.422 | N3 | -0.288 | N4 | -0.334 |
| N3 | -0.336 | N4 | -0.267 | N5 | -0.331 |
| N4 | -0.340 | N5 | -0.467 | N6 | -0.466 |
| N5 | -0.413 | N6 | -0.307 | N7 | -0.283 |
| N6 | -0.501 | N7 | -0.262 | N8 | -0.287 |
| N7 | -0.460 | N8 | -0.328 | N9 | -0.329 |
| N8 | -0.335 | N9 | -0.339 | N10 | -0.331 |
| N9 | -0.333 | N10 | -0.329 | N11 | -0.306 |
| N10 | -0.338 | N11 | -0.305 | N12 | -0.347 |
| N11 | -0.335 | N12 | -0.283 | N13 | -0.503 |
| N12 | -0.418 | N13 | -0.354 | N14 | -0.304 |
| N13 | -0.456 | N14 | -0.504 | N15 | -0.351 |
| N14 | -0.502 | N15 | -0.360 | N16 | -0.300 |
| N15 | -0.333 | N16 | -0.356 | O1 | -0.357 |
| N16 | -0.334 | N17 | -0.316 | | |
| N17 | -0.417 | | | | |
| N18 | -0.364 | | | | |

**Table S3.** Fitting results of equivalent circuit to the impedance data of UCN, CCN, and OCCN.

| Sample | $R_s$ (Ω cm$^2$) | $R_{ct}$ (Ω cm$^2$) | $C_d$ |
|---|---|---|---|
| UCN | 42.0 | 49051 | 0.83 |
| CCN | 44.5 | 25707 | 0.81 |
| OCCN | 24.2 | 11126 | 0.80 |

**Table S4.** Comparison of $H_2O_2$ production rate of OCCN with reported catalysts.

| Catalyst | Reactant solution | Light Source | $H_2O_2$ yield (μmol $g^{-1}$ $h^{-1}$) | AQE (%) | Ref. |
|---|---|---|---|---|---|
| OCCN | $H_2O$: IPA (9/1, 100 mL) | 300 W Xe lamp, λ>420 nm | 13136.4 | 27.20 (420 nm) | This wok |
| C-P-CN | $H_2O$: EtOH (9/1, 50 mL) | 300 W Xe lamp, λ>400 nm | 3320 | / | [10] |
| O-CNC4 | $H_2O$: IPA (9/1, 50 mL) | 300 W Xe lamp | 2008.4 | / | [11] |
| g-CN-M | $H_2O$: IPA (9/1, 30 mL) | 300 W Xe lamp | 6320 | / | [12] |
| NB-400 | $H_2O$: IPA (9/1, 100 mL) | 300 W Xe lamp, λ>420 nm | 455 | / | [13] |
| $Ti_3C_2$/g-$C_3N_4$ | $H_2O$: IPA (9/1, 50 mL) | 300 W Xe lamp, λ>420 nm | 2634.2 | / | [14] |
| ACN | $H_2O$: IPA (9/1, 50 mL) | 300 W Xe lamp | 1874 | 4.4 (420 nm) | [15] |
| KDCN-0.2 | $H_2O$: IPA (9/1, 100 mL) | 300 W Xe lamp, λ>420 nm | 557.8 | / | [16] |
| COF TTA-TTTA | $H_2O$: EtOH (9/1, 50 mL) | 420 nm LED | 4347 | / | [17] |
| $TF_{50}$-COF | $H_2O$: EtOH (9/1, 50 mL) | 350 W Xe lamp, λ>420 nm | 1739 | / | [18] |
| TZ-COF | $H_2O$: BA (1/1, 30 mL) | Xe lamp, λ>420 nm | 4951 | 0.6 (475 nm) | [19] |
| TAPT-TFPA COFs@Pd ICs | $H_2O$: EtOH (9/1, 20 mL) | 300 W Xe lamp, λ>400 nm | 2143 | 6.5 (400 nm) | [20] |
| $PD^{2+}$-$COF_{16.7}$ | $H_2O$: EtOH (9/1, 50 mL) | 300 W Xe lamp, λ=400-700 nm | 11965 | / | [21] |
| PMCR-1 | $H_2O$: BA (10/1, 22 mL) | 300 W Xe lamp, λ>420 nm | 5500 | / | [22] |
| DVA-COF | $H_2O$: BA (9/1, 20 mL) | 420 nm LED | 8450 | 2.84 (420 nm) | [23] |

**Table S5.** Calculated AQE of OCCN at different light wavelengths.

| λ (nm) | S ($cm^2$) | I (mW $cm^{-2}$) | t (s) | n ($H_2O_2$-μmol $h^{-1}$) | AQE (%) |
|---|---|---|---|---|---|
| | | | | 89.64 | 32.5 |
| 395 | 12.57 | 3.70 | 3600 | 92.42 | 33.5 |
| | | | | 86.33 | 31.3 |
| | | | | 116.34 | 27.2 |
| 420 | 15.90 | 4.27 | 3600 | 121.26 | 28.4 |
| | | | | 124.72 | 29.2 |
| | | | | 0.21 | 0.2 |
| 500 | 12.57 | 1.10 | 3600 | 0.37 | 0.4 |
| | | | | 0.37 | 0.4 |

**Table S6.** Photocatalytic performance of reported systems for $H_2O_2$ production from $H_2O$ and $O_2$.

| Photocatalysts | Cat./g $L^{-1}$ | Temprature (K) | Light Source | SCC | Ref. |
|---|---|---|---|---|---|
| COF-TfpBpy | 1.5 | 298 | Xe lamp, λ>420 nm | 0.57% | [24] |
| TpDz | 0.167 | / | 300 W Xe lamp, λ>420 nm | 0.62% | [25] |
| Bpy-TAPT | 5 | / | 300 W Xe lamp, λ>420 nm | 0.65% | [26] |
| RF-CN-bm | 1.67 | / | 300 W Xe lamp, AM 1.5G | ~0.5% | [27] |
| LCAP-2 | 10 | / | 300 W Xe lamp, AM 1.5G | 0.59% | [28] |
| CoOx/Mo:$BiVO_4$/ Pd | 2 | 285 | AM 1.5G | 0.29% | [29] |
| ZBTO-Pt | 0.34 | / | λ>380 nm | 0.10% | [30] |
| ZBTO-FeOx | 0.34 | / | λ>380 nm | 0.06% | [30] |
| OCCN | 1 | 298 | AM 1.5G | 0.69% | This work |

## REFERENCES


1. Wei Z, Liu M, Zhang Z *et al.* Efficient visible-light-driven selective oxygen reduction to hydrogen peroxide by oxygen-enriched graphitic carbon nitride polymers. *Energy Environ. Sci.* 2018; **11**: 2581-2589.
2. Li J, Zhan G, Yu Y *et al.* Superior visible light hydrogen evolution of Janus bilayer junctions via atomic-level charge flow steering. *Nat. Commun.* 2016; **7**: 11480.
3. Delley B A scattering theoretic approach to scalar relativistic corrections on bonding. *Int. J. Quantum Chem.* 1998; **69**: 423-433.
4. Delley B An all-electron numerical method for solving the local density functional for polyatomic molecules. *J. Chem. Phys.* 1990; **92**: 508-517.
5. Zhang G, Li G, Lan Z *et al.* Optimizing optical absorption, exciton dissociation, and charge transfer of a polymeric carbon nitride with ultrahigh solar hydrogen production activity. *Angew. Chem. Int. Ed.* 2017; **56**: 13445-13449.
6. Pan R L, Lv W, Ge X *et al.* Electron-deficient engineering in large-conjugate-heptazine framework to effectively shuttle hot electrons for efficient photocatalytic $H_2O_2$ production. *Adv. Funct. Mater.* 2024; **35**: 2414193.
7. Chen J, Dong C L, Zhao D M *et al.* Molecular design of polymer heterojunctions for efficient solar-hydrogen conversion. *Adv. Mater.* 2017; **29**: 1606198.
8. Nagasaka M, Yuzawa H and Kosugi N Microheterogeneity in aqueous acetonitrile solution probed by soft X-ray absorption spectroscopy. *J. Phys. Chem. B* 2020; **124**: 1259-1265.
9. He T W, Tang H C, Wu J *et al.* A metal-free cascaded process for efficient $H_2O_2$ photoproduction using conjugated carbonyl sites. *Nat. Commun.* 2024; **15**: 1-12.
10. Wei W, Zou L, Li J *et al.* Dual molecules engineered carbon nitride for achieving outstanding photocatalytic $H_2O_2$ production. *J. Colloid. Interf. Sci.* 2023; **636**: 537-548.
11. Xie H, Zheng Y, Guo X *et al.* Rapid microwave synthesis of mesoporous oxygen-doped g-$C_3N_4$ with carbon vacancies for efficient photocatalytic $H_2O_2$ production. *ACS Sustain. Chem. Eng.* 2021; **9**: 6788-6798.
12. Zhang H, Wu P, He J *et al.* Highly dispersible graphitic carbon nitride: Synthesis and its 2-electron photocatalytic reduction activity of $O_2$. *J. Environ. Chem. Eng.* 2021; **9**: 106430.

13. Luo J, Fan C, Tang L *et al.* Reveal bronsted-evans-polanyi relation and attack mechanisms of reactive oxygen species for photocatalytic $H_2O_2$ production. *Appl. Catal. B-Environ.* 2022; **301**: 120757.

14. Yang Y, Zeng Z, Zeng G *et al.* $Ti_3C_2$ Mxene/porous g-$C_3N_4$ interfacial Schottky junction for boosting spatial charge separation in photocatalytic $H_2O_2$ production. *Appl. Catal. B-Environ.* 2019; **258**: 117956.

15. Zheng Y, Luo Y, Ruan Q *et al.* Plasma-induced hierarchical amorphous carbon nitride nanostructure with two $N_{2C}$-site vacancies for photocatalytic $H_2O_2$ production. *Appl. Catal. B-Environ.* 2022; **311**: 121372.

16. Pan Y, Liu X J, Zhang W *et al.* Bifunctional template-mediated synthesis of porous ordered g-$C_3N_4$ decorated with potassium and cyano groups for effective photocatalytic $H_2O_2$ evolution from dual-electron $O_2$ reduction. *Chem Eng J* 2022; **427**: 132032.

17. Tan F, Zheng Y, Zhou Z *et al.* Aqueous synthesis of covalent organic frameworks as photocatalysts for hydrogen peroxide production. *CCS Chem.* 2022; **4**: 3751-3761.

18. Wang H, Yang C, Chen F *et al.* A crystalline partially fluorinated triazine covalent organic framework for efficient photosynthesis of hydrogen peroxide. *Angew. Chem. Int. Ed.* 2022; **61**: e202202328.

19. Mou Y, Wu X, Qin C *et al.* Linkage microenvironment of azoles-related covalent organic frameworks precisely regulates photocatalytic generation of hydrogen peroxide. *Angew. Chem. Int. Ed.* 2023; **62**: e202309480.

20. Liu Y, Li L, Tan H *et al.* Fluorination of covalent organic framework reinforcing the confinement of Pd nanoclusters enhances hydrogen peroxide photosynthesis. *J. Am. Chem. Soc.* 2023; **145**: 19877-19884.

21. Zhang Y, Cao N, Liu X *et al.* Enhanced electron density of the π-conjugated structure and in-plane charge transport to boost photocatalytic $H_2$ evolution of g-$C_3N_4$. *Sci. China Mater.* 2023; **66**: 2274-2282.

22. Yue J, Song L, Fan Y *et al.* Thiophene-containing covalent organic frameworks for overall photocatalytic $H_2O_2$ synthesis in water and seawater. *Angew. Chem. Int. Ed.* 2023; **62**: e202309624.

23. Yu H, Zhang F, Chen Q *et al.* Vinyl-group-anchored covalent organic framework for promoting the photocatalytic generation of hydrogen peroxide. *Angew. Chem. Int. Ed.* 2024; **63**: e202402297.
24. Kou M P, Wang Y Y, Xu Y X *et al.* Molecularly engineered covalent organic frameworks for hydrogen peroxide photosynthesis. *Angew. Chem. Int. Ed.* 2022; **61**: e202200413.
25. Liao Q B, Sun Q N, Xu H C *et al.* Regulating relative nitrogen locations of diazine functionalized covalent organic frameworks for overall $H_2O_2$ photosynthesis. *Angew. Chem. Int. Ed.* 2023; **62**: e202310556.
26. Liu Y, Han W K, Chi W W *et al.* Substoichiometric covalent organic frameworks with uncondensed aldehyde for highly efficient hydrogen peroxide photosynthesis in pure water. *Appl Catal B-Environ* 2023; **331**: 122691.
27. Su P D, Zhang J K, Zhou Y H *et al.* Efficient photocatalytic production of hydrogen peroxide by Z-scheme resorcinol-formaldehyde resin/g-$C_3N_4$ heterostructure under visible light. *Chem. Eng. J.* 2023; **454**: 140504.
28. Jin Z Q, Jin S J, Tang X F *et al.* Rational design of conjugated acetylenic polymers enables a two-electron water oxidation pathway for enhanced photosynthetic hydrogen peroxide generation. *Small* 2024; **20**: 2305004.
29. Liu T, Pan Z H, Vequizo J J M *et al.* Overall photosynthesis of $H_2O_2$ by an inorganic semiconductor. *Nat. Commun.* 2022; **13**: 1034.
30. Zhou X F, Yan F, Shen B *et al.* Enhanced sunlight-driven reactive species generation via polarization field in nanopiezoelectric heterostructures. *Acs Appl Mater Inter* 2021; **13**: 29691-29707.
